# 5-Aminopyrazole Dimerization: Cu-Promoted Switchable Synthesis of Pyrazole-Fused Pyridazines and Pyrazines via Direct Coupling of C-H/N-H, C-H/C-H, and N-H/N-H Bonds

**DOI:** 10.3390/molecules30020381

**Published:** 2025-01-17

**Authors:** Yi-Xin Chai, Jun-Jie Ren, Yi-Ming Li, Yi-Cheng Bai, Qing-Qing Zhang, Yi-Zhen Zhao, Xue Yang, Xiao-Han Zhang, Xin-Shuang Zhang, An-Xin Wu, Yan-Ping Zhu, Yuan-Yuan Sun

**Affiliations:** 1 Key Laboratory of Molecular Pharmacology and Drug Evaluation, Ministry of Education, School of Pharmacy, Yantai University, Yantai 264005, China; ckfsj03@163.com (Y.-X.C.); jjren@yic.ac.cn (J.-J.R.); lym19861109129@163.com (Y.-M.L.); drugbaiyc@163.com (Y.-C.B.); 19712028152@163.com (Q.-Q.Z.); 13573588751@163.com (Y.-Z.Z.); 19558517729@139.com (X.Y.); 18963556591@163.com (X.-H.Z.); 13816123757@163.com (X.-S.Z.); 2 International Innovation Center for Forest Chemicals and Materials, Nanjing Forestry University, Nanjing 210037, China; 3 National Key Laboratory of Green Pesticide, International Joint Research Center for Intelligent Biosensor Technology and Health, College of Chemistry, Central China Normal University, Wuhan 430079, China

**Keywords:** Cu-promoted chemoselective dimerization, C-H/N-H, C-H/C-H, and N-H/N-H direct coupling, switchable products, fused pyridazines and pyrazines

## Abstract

A Cu-promoted highly chemoselective dimerization of 5-aminopyrazoles to produce pyrazole-fused pyridazines and pyrazines is reported. The protocol generates switchable products via the direct coupling of C-H/N-H, C-H/C-H and N-H/N-H bonds, with the merits of broad substrate scope and high functional group compatibility. Gram-scale experiments demonstrated the potential applications of this reaction. Moreover, the preliminary fluorescence results uncovered that dipyrazole-fused pyridazines and pyrazines may have some potential applications in materials chemistry.

## 1. Introduction

**Figure 1:**
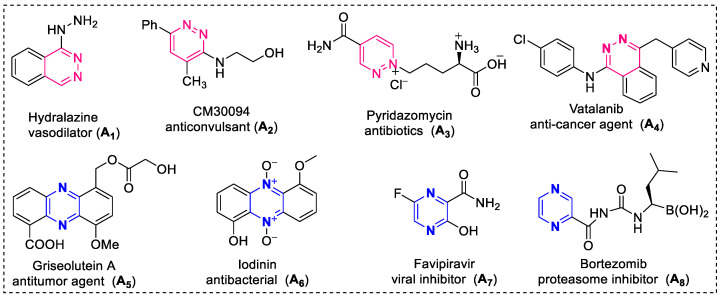
Selected examples of bioactive pyridazines (A_1_–A_4_) and pyrazines (A_5_–A_8_) containing molecules.

Nitrogen-containing heterocyclic molecules have received a great deal of attention from the synthetic chemistry community due to the large-scale biological activities they possess [1]. Pyridazines and pyrazines, and important subsets thereof, are widely used in synthetic pharmaceuticals, chemicals, dyes, materials, etc., which have an important place in academia and industry. These compounds have been shown to be vasodilators (**A_1_**) [2], anticonvulsant (**A_2_**) [3], antibiotics (**A_3_**) [4], anticancer agents (**A_4_**–**A_5_**) [5,6], antimicrobial agents (**A_6_**) [7], virus inhibitors (**A_7_**) [8], and proteasome inhibitors (**A_8_**) [9] (Figure 1). Thus, pyridazine and pyrazine scaffolds have become hot research topics and have triggered continuous synthetic innovations.

**Scheme 1:**
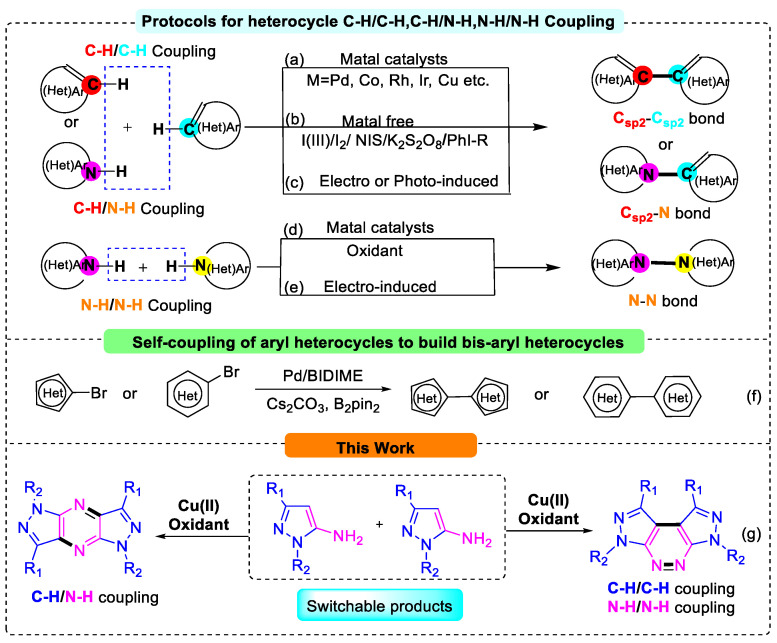
The reported strategies for the synthesis of C(sp^2^)-C(sp^2^)/C(sp^2^)-N/N-N bonds via cross dehydrogenative coupling (**a**–**f**) and this work (**g**).

The construction of C-C, C-N, and N=N bonds has been a hot topic in the research of medicinal chemistry over past decades, in which the direct functionalization of C-H bond applied to dehydrogenation coupling reactions has made great progress [10,11,12,13,14,15]. Among them, Csp^2^-Csp^2^ bond is essential for the synthesis of *π*-conjugated molecules, and Csp^2^-N bond is prevalent in pharmaceutically active molecules. Various methods exist for constructing Csp^2^-Csp^2^ and Csp^2^-N bonds, with the coupling of functionalized dehydrogenation from Csp^2^-H being one of the most powerful and reliable approaches. The classical method is transition metal catalysis, such as palladium, iridium, copper, silver, manganese, etc. (Scheme 1a) [16,17,18,19,20,21,22,23]. Metal-free synthetic approaches to construct Csp^2^-Csp^2^ bond and Csp^2^-N bond that utilize inorganic catalysts, such as I_2_, I (III), K_2_S_2_O_8_, NIS, etc., have also been reported via the dehydrogenation coupling reaction (Scheme 1b) [24,25,26,27]. For instance, Liang and co-workers developed a facile iodine-promoted regioselective C-H/C-H and C-H/N-H coupling of indoles with indoles or amines to give 2,3′-biindoles and 4-(1*H*-indol-2-yl)morpholines [28]. As the concept of green synthesis is becoming more and more popular, photocatalysis and electrochemistry-induced Csp^2^-H functionalized dehydrogenation coupling were developed (Scheme 1c) [29,30,31,32,33]. For instance, Li and co-workers developed a cobalt-promoted electrochemical 1,2-diarylation of alkenes with electron-rich aromatic hydrocarbons via direct dual C-H functionalization to generate polyaryl-functionalized alkanes [34]. Lei’s group has successively reported the synthesis of imidazopyridines, triarylamine derivatives, and polycyclic indoline derivatives via electro-oxidation dehydrogenation [35,36,37].

Hydrazine derivatives have diverse applications in fields such as pharmaceuticals and dyes. In recent years, compared to conventional metal catalysts (Scheme 1d) [38,39], the electrochemical dehydrogenation of N-H/N-H coupling has become a powerful tool for the construction of N-N bond (Scheme 1e) [40,41]. For example, Chen’s group reported an electrochemically induced N-H/N-H dehydrogenation strategy of *N*-phenylpyridin-2-amine to construct N-N bond for the synthesis of hydrazine analogs [42]. Additionally, the self-coupling of brominated arylheterocycles to build bis-arylheterocyclic fragments has also received much attention in this field. For example, Tang’s group developed an effective Pd-catalyzed homocoupling of heteroaryl bromides for the formation of a series of symmetrical biheteroaryls, featuring a tandem Miyaura borylation, known as the Suzuki coupling sequence (Scheme 1f) [43].

Despite the abovementioned good achievements, approaches for synthesizing pyrazole-fused pyridazines or pyrazines are rarely reported. Recently, Baruah’s group developed a Cu(I)-catalyzed annulation of aminopyrazoles to synthesize pyridazine in the presence of Ag_2_CO_3_ and benzoic acid; furthermore, in the absence of benzoic acid, intermediate *E*-diazo compounds are generated [44]. However, the method for synthesizing pyrazole-fused pyrazines has not yet been reported. As shown in Scheme 1g, herein we describe the example of the Cu-catalyzed highly chemoselective dimerization of 5-aminopyrazoles to produce dipyrazole-fused pyridazines and pyrazines. The protocol generates switchable products via the direct coupling of C-H/N-H, C-H/C-H, and N-H/N-H bonds.

## 2. Results and Discussion

3-Methyl-1-phenyl-1*H*-pyrazol-5-amine **1a** was selected as the model substrate for the screening and optimization of the reaction conditions. Under the initial conditions shown in entry 1 of Table S1 for **1a** (0.2 mmol) using Cu(OAc)_2_ as a catalyst, the desired product **2a** was obtained with a yield of 50%. Subsequently, we investigated the effect of the type of oxidants on the reaction; it was found that when benzoyl peroxide was used as an oxidant, a yield of 58% was achieved (Table 1, entries 2–5). Then, the different catalyst types were studied, and it was found that the yields were all reduced (Table 1, entries 6–12). The effect of temperature on the method was also tested. These results showed that the optimal reaction temperature was 100 °C (Table 1, entries 13–16). When continuing the study of the equivalent of Cu(OAc)_2_, it was found that the yield was decreased when the amount of Cu(OAc)_2_ was decreased (Table 1, entries 17–18). Benzoyl peroxide equivalents were also tested, and the best yield was found at 0.5 equivalent (Table 1, entries 19–20). The solvents were also investigated; the yields did not improve well (Table 1, entries 21–25). Finally, the effect of adding three different additives was tested, and it was found that the addition of K_2_S_2_O_8_ resulted in a significant increase in yield (Table 1, entries 26–28). Following this, the equivalent of K_2_S_2_O_8_ was investigated and it was found that the highest yield was achieved when the equivalent of K_2_S_2_O_8_ was 2.5 (Table 1, entries 29–30). Finally, we found the following optimal conditions to synthesize **2a** [45]: 3-methyl-1-phenyl-1*H*-pyrazol-5-amine (**1a**) (0.2 mmol), Cu(OAc)_2_ (3.0 equiv.), benzoyl peroxide (BPO) (0.5 equiv.), and K_2_S_2_O_8_ (2.5 equiv.), and these were heated in toluene (2 mL) at 100 °C for 10 h under air.

**Figure 2:**
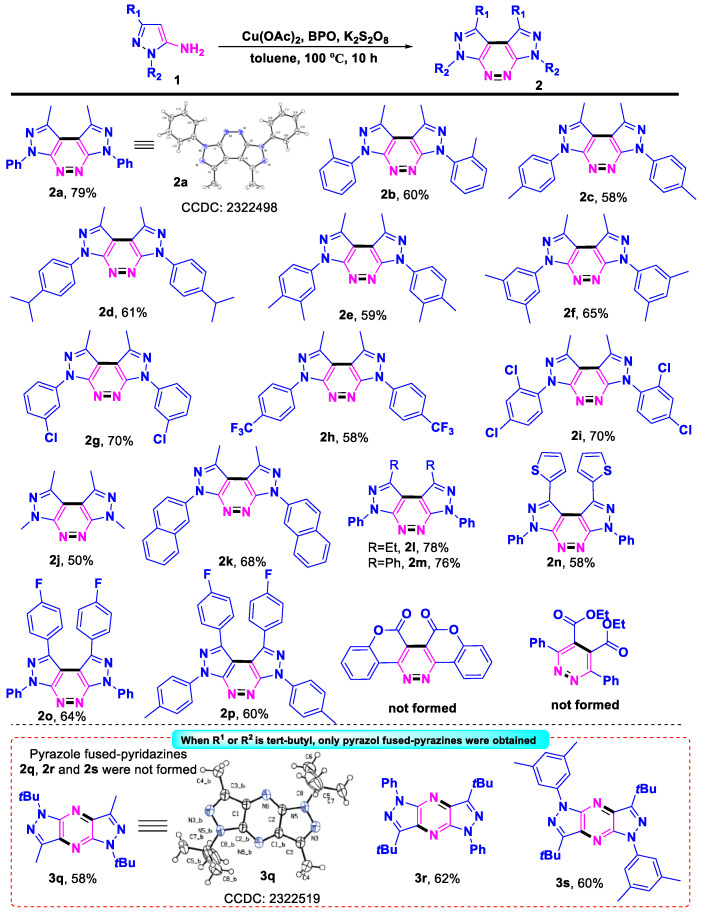
Scope of dipyrazole-fused pyridazines. Reaction conditions: **1a** (0.2 mmol), Cu(OAc)_2_ (3.0 equiv.), benzoyl peroxide (BPO) (0.5 equiv.), and K_2_S_2_O_8_ (2.5 equiv.) in toluene at 100 °C for 10 h. Isolated yields provided.

After the optimal reaction conditions were determined, the substrate scope of 5-aminopyrazoles was explored to synthesize dipyrazole-fused pyridazines (Figure 2). Firstly, when R_1_ is the methyl group, the reactions of different substituents on R_2_ were tested. To our delight, the protocol showed excellent tolerance to substituted phenyls containing electron-donating and electron-absorbing groups. All substrates proceeded smoothly to afford the desired products in moderate to good yields (**2a**–**2i**, 58–79%); when R_2_ is the methyl group, the yield of product **2j** is 50%. Moreover, when R_2_ is naphthalene, the target product can be obtained with a good yield (**2k**, 70%). When R_2_ is phenyl and R_1_ is ethyl, phenyl, thienyl, and 4-*F*-phenyl, respectively, all reactions gave the target products in moderate to good yields (**2l**–**2o**, 58–78%). Finally, when R_1_ is the 4-*F*-phenyl group and R_2_ is the 4-methylphenyl group, **2p** can still be obtained in 60% yield. It should be mentioned that when either R_1_ or R_2_ is the tert-butyl group, perhaps because of the site resistance, dipyrazole-fused pyridazines **2q**, **2r**, and **2s** were not formed; only new isomer dipyrazole-fused pyrazines were obtained in moderate yields (**3q**–**3s**, 58–62%). Moreover, the compound **3q** [45] was further confirmed by X-ray crystallographic analysis. In addition, 4-amino-2*H*-chromen-2-one and ethyl (*E*)-3-amino-3-phenylacrylate are not applicable to this reaction, and no target compounds are formed.

Due to the emergence of new configurations, this has sparked a great deal of interest and prompted us to subject **3a** to condition optimization. 3-Methyl-1-phenyl-1*H*-pyrazol-5-amine **1a** was still chosen as the substrate model. Under the initial conditions shown in entry 1 of Table 2 for **1a** (0.2 mmol) using Cu(OAc)_2_ as a catalyst, the desired product 3a was obtained with a yield of 36%. Different oxidants were then tested, and the addition of tert-butyl peroxybenzo resulted in an increase in yield (Table 2, entries 2–5). Subsequently, different Cu catalysts were experimented on, and it was found that the yield was enhanced when CuCl_2_ was used as the catalyst (Table 2, entries 6–10). Then, the addition of different additives was used to test the experimental effect, and it was found that the addition of Na_2_CO_3_ promotes the reaction and results in an increase in yield (Table 2, entries 11–15). Different solvents were also screened, but all resulted in lower yields (Table 2, entries 16–22). Subsequently, it was found that the addition of 1,10-phenantnroline leads to a higher increase in yield (Table 2, entries 23–24). Based on this, we changed the temperature to observe the experimental effect and found that the optimal reaction temperature was still 130 °C (Table 2, entries 25–27). Finally, the yield decreased when the equivalent of CuCl_2_ was increased to 40% or decreased to 10% (Table 2, entries 28–29). We screened the conditions to obtain the best solution to synthesize **3a** [45]: **1a** (0.2 mmol), CuCl_2_ (20 mol%), 1,10-phenanthroline (0.3 equiv.), tert-butyl peroxybenzoate (0.5 equiv.), and Na_2_CO_3_ (2.5 equiv.) were heated in toluene (2 mL) at 130 °C for 12 h under air.

**Figure 3:**
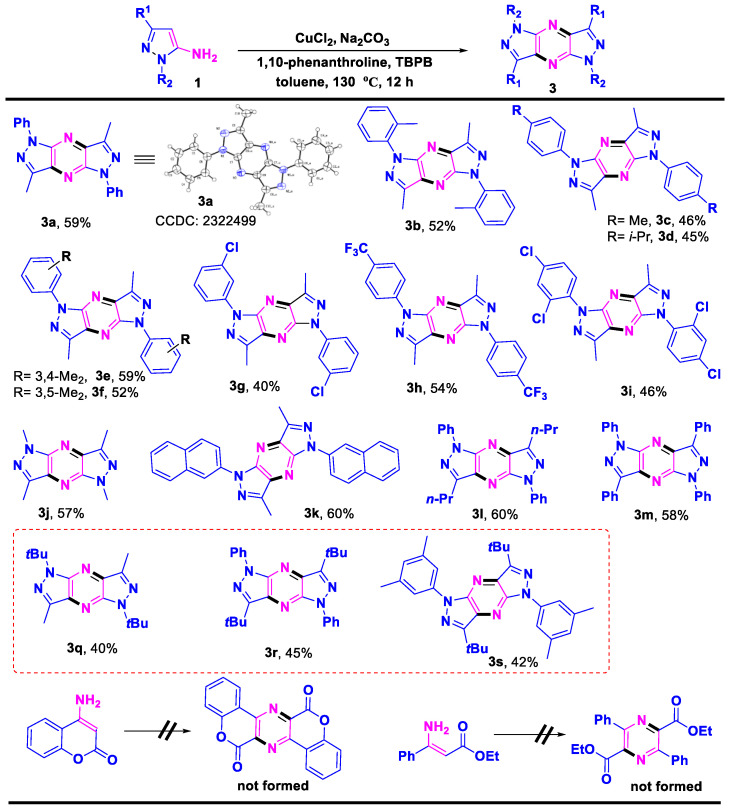
Scope of dipyrazole-fused pyrazines. Reaction conditions: **1a** (0.2 mmol), CuCl_2_ (20 mol%), 1,10-phenanthroline (0.3 equiv.), *tert-butyl* peroxybenzoate (0.5 equiv.), and Na_2_CO_3_ (2.5 equiv.) in toluene at 130 °C for 12 h. Isolated yields provided.

Next, due to the emergence of new configurations, we tested different species of 5-aminopyrazoles to synthesize dipyrazole-fused pyrazines (Figure 3). When R_1_ is a methyl group, different substituted substrates (e.g., 2-Me, 4-Me, 4-*i*Pr, 3,4-(Me)_2_, 3,5-(Me)_2_, 3-Cl, 4-CF_3_, 2,4-Cl_2_) on phenyl rings **1b**–**1i** could participate in this reaction, giving the desired products **3b**–**3i** in 40–59% yields. To our delight, compared to phenyl-substituted 5-aminopyrazoles, methyl- and naphthalene-substituted substrates also worked well, giving the corresponding products in moderate yields (**3j**–**3k**, 57–60%). When R_2_ is phenyl, whether R_1_ is alkyl or aryl (e.g., -*n*Pr, -Ph), these substrates performed the reactions smoothly to give the desired products in medium yields (**3l**–**3m**, 58–60%). Meanwhile, in this transformation process, whether R_1_ or R_2_ is tert-butyl can also be tolerated, leading to the final products being in moderate yields (**3q**–**3s**, 40–45%). In the same way, under the conditions shown in Figure 3, both 4-amino-2*H*-chromen-2-one and ethyl (*E*)-3-amino-3-phenylacrylate cannot produce the desired target products.

**Scheme 2:**
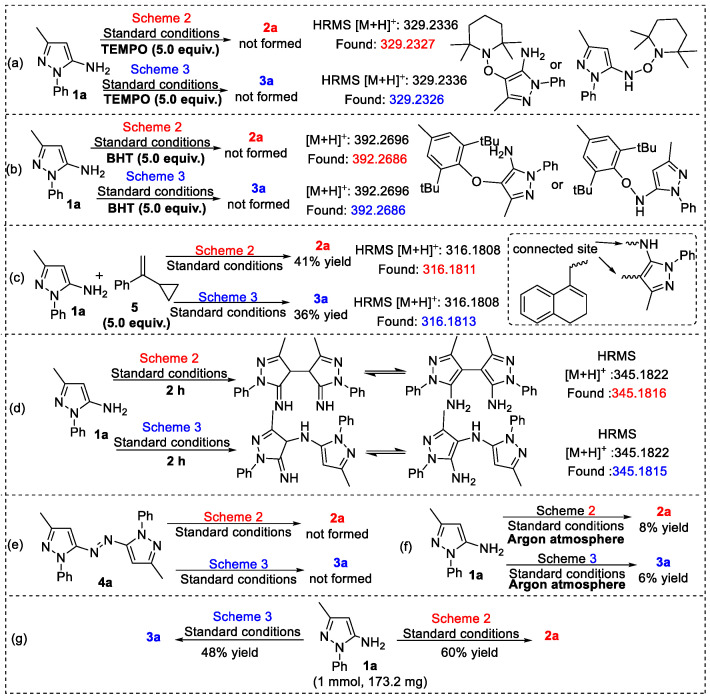
Control experiments (**a**–**f**) and the scale-up reactions (**g**).

**Scheme 3:**
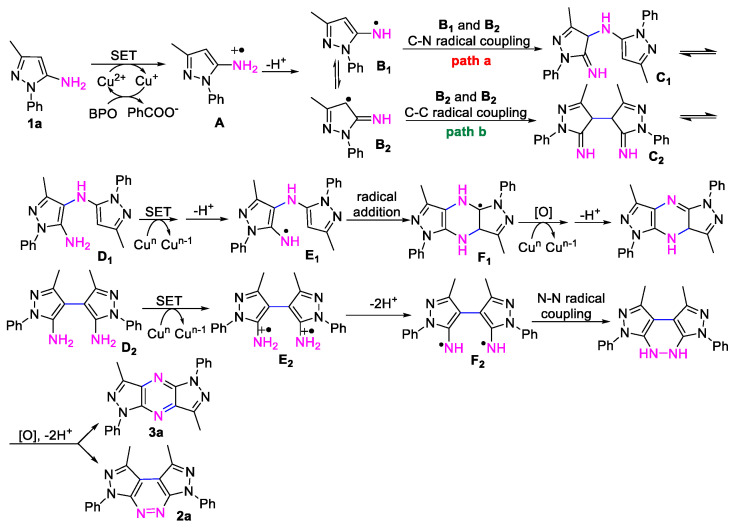
Proposed mechanism.

To establish the mechanism, several control experiments were conducted. The addition of the radical scavenger TEMPO or BHT (5.0 equiv.) under the standard conditions of Scheme 2 or 3, respectively, was found to completely inhibit the formation of **2a** or **3a**. The crude reaction solution was subjected to HRMS, with the product of **1a** binding to TEMPO or BHT, which could be found under the two conditions, respectively (Scheme 2a and 2b). This demonstrated that this reaction was performed involving the radical pathway. Then, **1a** was reacted with (1-cyclopropylvinyl) benzene **5** (5.0 equiv.) under Scheme 2 or Scheme 3 standard conditions, and the results showed that the yields of both **2a** and **3a** were decreased, and the adduct could be detected in the crude reaction solution through HRMS (Scheme 2c). Moreover, the production of potential intermediates was, respectively, detected by HRMS after 2 h of the Scheme 2 or Scheme 3 reactions (Scheme 2d). We assessed whether the azo compound (*E*)-1,2-bis(3-methyl-1-phenyl-1*H*-pyrazol-5-yl) diazene **4a** could produce **2a** or **3a** under Scheme 2 or Scheme 3 standard conditions; the results showed that **2a** or **3a** was not produced, suggesting that **4a** is not a reaction intermediate (Scheme 2e). We then tested the reaction with Scheme 2 or Scheme 3 standard conditions under argon atmosphere and found that the yield was only 8% for **2a** and 6% for **3a**, thus demonstrating that oxygen is necessary for the reaction to proceed (Scheme 2f). As shown in Scheme 2g, when the amount of substrate **1a** was scaled-up to 1.0 mmol, the products **2a** and **3a** could still be obtained in 60% and 48% yields, respectively.

Based on our observations and literature reports [46,47,48], a plausible reaction mechanism is depicted (Scheme 3). 5-Aminopyrazole (**1a**) reacted with Cu(II) to give intermediate **A** through a SET pathway. After the deprotonation, N radical **B_1_** or C radical **B_2_** may form. In path a, **B_1_** and **B_2_** underwent C-N radical coupling to generate intermediate **C_1_**, which could undergo structural mutual transformation to obtain **D_1_**. **D_1_** underwent Cu-mediated one-electron oxidation and deprotonation to obtain N radical **E_1_**. Subsequently, **F_1_** was obtained by radical addition. **3a** could finally be achieved through successive Cu-mediated SET, deprotonation, and oxidative dehydrogenation from intermediate **F_1_**. In path b, **B_2_** and **B_2_** underwent C-C radical coupling to generate intermediate **C_2_**, which could undergo structural mutual transformation to obtain **D_2_**. Amino cationic radical **E_2_** was obtained through Cu-mediated SET. **E_2_** lost two protons to obtain N radical **F_2_**. From **F_2_**, N-N radical coupling and oxidative dehydrogenation successively occurred to form **2a**.

**Figure 4:**
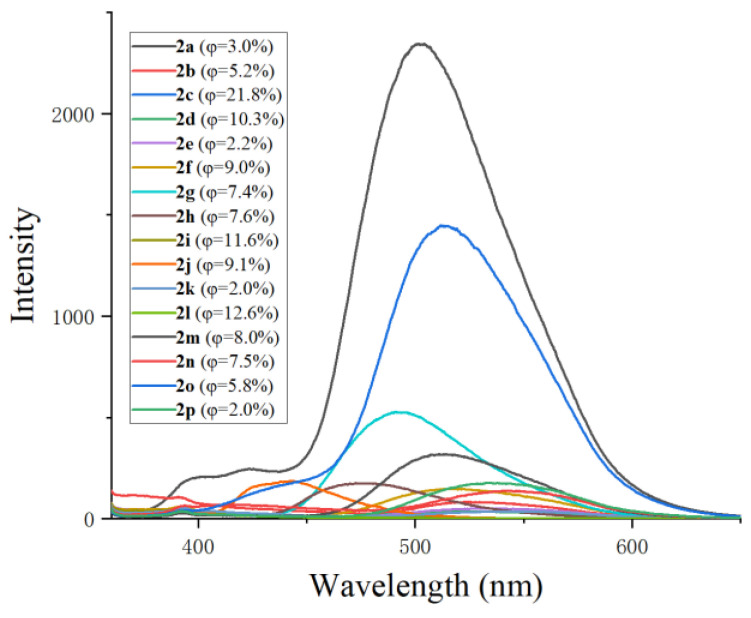
Fluorescence spectra of compounds **2** in CH_2_Cl_2_ (1 × 10^−5^ M) under UV irradiation (285 nm).

**Figure 5:**
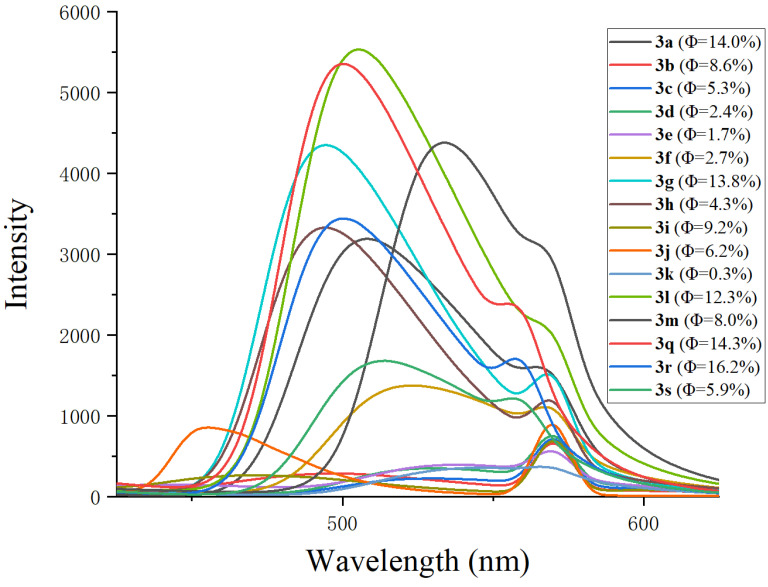
Fluorescence spectra of compounds **3** in CH_2_Cl_2_ (1 × 10^−5^ M) under UV irradiation (285 nm).

**Figure 6:**
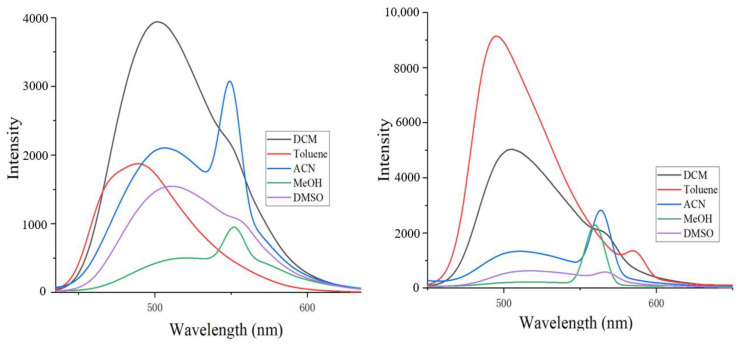
The fluorescence emission spectra of **2a** (**left**) and **3l** (**right**) in CH_2_Cl_2_, toluene, ACN, CH_3_OH, and DMSO at a concentration of 1 × 10^−5^ mol/L.

Most of the synthetic compounds showed a fluorescent nature during UV monitoring (see Supplementary Materials Figure S4). We proceeded to characterize their fluorescence performance (Figure 4 and Figure 5). The UV absorption wavelengths of compound **2** ranged from 266 to 309 nm. The fluorescence emission of them ranged from 293 to 511 nm (Table 3). In compound **2**, when R_1_ is a methyl group and R_2_ is a benzene ring, the quantum yield of most products with different substituents on the benzene ring (**2b**–**2d**, **2f**–**2i**) is higher than that of unsubstituted products (**2a**). The quantum yields of the bis-methyl-substituted product (**2j**) were slightly higher than those of the bis-phenyl-substituted product (**2m**). The UV absorption wavelengths of compound **3** ranged from 274 to 304 nm. The fluorescence emission of them ranged from 474 to 544 nm (Table 4). Interestingly, the above fluorescence quantum yield trend observed in compound **3** is opposite to that of compound **2**. Finally, we tested the effect of solvent polarity on the fluorescence spectra and found that compounds **2** and **3** both showed significant red and blue shifts in DCM and toluene, while the emission maxima in ACN, DMSO, and CH_3_OH were almost the same (Figure 6).

## 3. Materials and Methods

### 3.1. General Information

Analytical thin-layer chromatography (TLC) was performed by using pre-coated silica gel HF254 glass plates. Column chromatography was performed by using silica gel (200–300 mesh). The ^1^H NMR and ^13^C NMR spectra were recorded on a Bruker Advance 500 or 400 MHz instrument at 500 or 400 MHz (^1^H NMR) and 126 or 101 MHz (^13^C NMR). We used the residual solvent peak in CDCl_3_ as an internal reference (*δ* = 7.26 for ^1^H and *δ* = 77.0 for ^13^C{^1^H}). Chemical shifts (*δ*) are reported in ppm relative to the internal standard of tetramethylsilane (TMS). The coupling constants (*J*) are quoted in Hz (hertz). Resonances are described as s (singlet), d (doublet), t (triplet), q (quartet), m (multiplet), br (broad), or combinations thereof. High-resolution mass spectra (HRMS) were obtained on Thermo Scientific Q-Exactive (ESI mode, Q-Exactive Orbitrap MS system) (From Thermo Fisher Scientific, Shanghai, China). The melting points were measured with the SGW X-4 apparatus (From INESA, Shanghai, China). Data collection for the crystal structure was performed by using Mo K*α* radiation on a Bruker Smart APEX CCD area-detector diffractometer (From Bruker, Beijing, China). UV data were recorded by Shimadzu UV 2000 (From Shimadzu, Jiangsu, China) at ambient temperature, and fluorescence data were recorded by HITACHI F7000 (From HITACHI, Beijing, China) at ambient temperature.

### 3.2. Synthetic Procedures

**Scheme 4:**
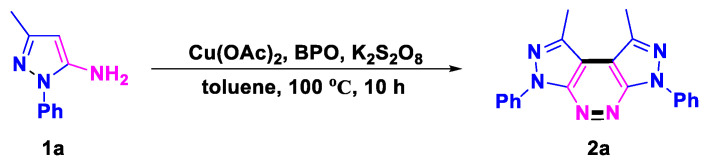
General procedure for synthesis of **2a**.

What follows is the general procedure for the synthesis of **2** (**2a** as an example). 3-Methyl-1-phenyl-1*H*-pyrazol-5-amine (**1a**) (35 mg, 0.2 mmol, 1.0 equiv.), Cu(OAc)_2_ (55 mg, 0.3 mmol, 3.0 equiv.), benzoyl peroxide (BPO) (12 mg, 0.05 mmol, 0.5 equiv.), and K_2_S_2_O_8_ (68 mg, 0.25 mmol, 2.5 equiv.) were added to a screw-cap test tube, and then the solvent of toluene (2.0 mL) was added. The mixture was then stirred at 100 °C in an oil bath for 10 h under air atmosphere. After the reaction was completed (monitored by TLC), 50 mL of water was added to the mixture, which was then extracted three times with CH_2_Cl_2_ (3 × 50 mL). The combined organic phase was dried with anhydrous Na_2_SO_4_, filtered, and concentrated under reduced pressure. The crude residues were purified by column chromatography (silica gel: 200–300 mesh, solvent system: petroleum ether/ethyl acetate = 5:1) to obtain the corresponding products **2a** (27 mg, 79%) (Scheme 4).

**Scheme 5:**
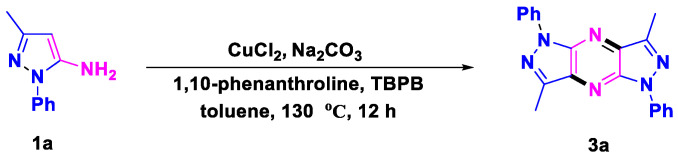
General procedure for synthesis of **3a**.

General procedure for the synthesis of **3** (**3a** as an example). 3-Methyl-1-phenyl-1*H*-pyrazol-5-amine (**1a**) (35 mg, 0.2 mmol, 1.0 equiv.), CuCl_2_ (3.0 mg, 0.02 mmol, 20 mol%), 1,10-phenanthroline (5.4 mg, 0.03 mmol, 0.3 equiv.), tert-butyl peroxybenzoate (10 uL, 0.05 mmol, 0.5 equiv.), and Na_2_CO_3_ (27 mg, 0.25 mmol, 2.5 equiv.) were added to a screw-cap test tube, and then the solvent of toluene (2.0 mL) was added. The mixture was then stirred at 130 °C in an oil bath for 12 h under air atmosphere. After the reaction was completed (monitored by TLC), 50 mL of water was added to the mixture, which was then extracted three times with CH_2_Cl_2_ (3 × 50 mL). The combined organic phase was dried with anhydrous Na_2_SO_4_, filtered, and concentrated under reduced pressure. The crude residues were purified by column chromatography (silica gel: 200–300 mesh, solvent system: petroleum ether/ethyl acetate = 10:1) to obtain the corresponding products **3a** (20mg, 59%) (Scheme 5).

### 3.3. Characterization of Products

*1,8-Dimethyl-3,6-diphenyl-3,6-dihydrodipyrazolo [3,4-c:4′,3′-e]pyridazine* (**2a**), (silica gel: 200–300 mesh, solvent system: petroleum ether/ethyl acetate = 5:1), 27 mg, 70%, yellow solid, m.p.: 242–243 °C. ^1^H NMR (400 MHz, CDCl_3_) *δ* (ppm) 8.46–8.41 (m, 4H), 7.60–7.55 (m, 4H), 7.40–7.35 (m, 2H), 3.01 (s, 6H). ^13^C NMR (126 MHz, CDCl_3_) *δ* (ppm) 150.6, 140.3, 139.2, 129.2, 126.6, 121.6, 109.7, 15.4. HRMS (ESI): *m*/*z* [M + H]^+^ calcd. for C_20_H_17_N_6_: 341.1509; found: 341.1505.

*1,8-Dimethyl-3,6-di-o-tolyl-3,6-dihydrodipyrazolo [3,4-c:4′,3′-e]pyridazine* (**2b**), (silica gel: 200–300 mesh, solvent system: petroleum ether/ethyl acetate = 5:1), 22 mg, 60%, yellow solid, m.p.: 220–221 °C. ^1^H NMR (400 MHz, CDCl_3_) *δ* (ppm) 7.50 (d, *J* = 7.2 Hz, 2H), 7.43–7.40 (m, 4H), 7.39–7.34 (m, 2H), 3.01 (s, 6H), 2.21 (s, 6H). ^13^C NMR (101 MHz, CDCl_3_) *δ* (ppm) 151.3, 140.1, 137.2, 135.5, 131.3, 129.2, 127.9, 126.6, 108.1, 18.4, 15.3. HRMS (ESI): *m*/*z* [M + H]^+^ calcd. for C_22_H_21_N_6_: 369.1822; found: 369.1812.

*1,8-Dimethyl-3,6-di-p-tolyl-3,6-dihydrodipyrazolo [3,4-c:4′,3′-e]pyridazine* (**2c**), (silica gel: 200–300 mesh, solvent system: petroleum ether/ethyl acetate = 5:1), 21 mg, 58%, yellow solid, m.p.: 272–273 °C. ^1^H NMR (500 MHz, CDCl_3_) *δ* (ppm) 8.27 (d, *J* = 8.5 Hz, 4H), 7.36 (d, *J* = 8.5 Hz, 4H), 2.98 (s, 6H), 2.44 (s, 6H). ^13^C NMR (126 MHz, CDCl_3_) *δ* (ppm) 150.4, 139.9, 136.8, 136.4, 129.8, 121.5, 109.4, 21.1, 15.4. HRMS (ESI): *m*/*z* [M + Na]^+^ calcd. for C_22_H_21_N_6_: 369.1822; found: 369.1816.

*3,6-Bis(4-isopropylphenyl)-1,8-dimethyl-3,6-dihydrodipyrazolo [3,4-c:4′,3′-e] pyridazine* (**2d**), (silica gel: 200–300 mesh, solvent system: petroleum ether/ethyl acetate = 5:1), 26 mg, 61%, yellow solid, m.p.: 227–228 °C. ^1^H NMR (500 MHz, CDCl_3_) *δ* (ppm) 8.29 (d, *J* = 8.5 Hz, 4H), 7.42 (d, *J* = 8.5 Hz, 4H), 3.05–3.00 (m, 2H), 2.99 (s, 6H), 1.33 (s, 6H), 1.31 (s, 6H). ^13^C NMR (101 MHz, CDCl_3_) *δ* (ppm) 150.5, 147.5, 140.0, 137.0, 127.2, 121.7, 109.4, 33.8, 24.0, 15.4. HRMS (ESI): *m*/*z* [M + H]^+^ calcd. for C_26_H_29_N_6_: 425.2448; found: 425.2440.

*3,6-Bis(3,4-dimethylphenyl)-1,8-dimethyl-3,6-dihydrodipyrazolo [3,4-c:4′,3′-e] pyridazine* (**2e**), (silica gel: 200–300 mesh, solvent system: petroleum ether/ethyl acetate = 5:1), 23 mg, 59%, yellow solid, m.p.: 269–270 °C. ^1^H NMR (400 MHz, CDCl_3_) *δ* (ppm) 8.10 (d, *J* = 6.4 Hz, 4H), 7.31 (d, *J* = 8.8 Hz, 2H), 2.98 (s, 6H), 2.40 (s, 6H), 2.35 (s, 6H). ^13^C NMR (101 MHz, CDCl_3_) *δ* (ppm) 150.5, 139.8, 137.6, 137.0, 135.2, 130.2, 122.8, 119.3, 109.3, 20.1, 19.4, 15.4. HRMS (ESI): *m*/*z* [M + H]^+^ calcd. for C_24_H_25_N_6_: 397.2135; found: 397.2125.

*3,6-Bis(3,5-dimethylphenyl)-1,8-dimethyl-3,6-dihydrodipyrazolo [3,4-c:4′,3′-e] pyridazine* (**2f**), (silica gel: 200–300 mesh, solvent system: petroleum ether/ethyl acetate = 5:1), 25 mg, 63%, yellow solid, m.p.: 272–273 °C. ^1^H NMR (400 MHz, CDCl_3_) *δ* (ppm) 7.99 (s, 4H), 7.02 (s, 2H), 2.99 (s, 6H), 2.46 (s, 12H). ^13^C NMR (101 MHz, CDCl_3_) *δ* (ppm) 150.5, 140.0, 139.0, 128.5, 119.6, 109.4, 29.7, 21.5, 15.4. HRMS (ESI): *m*/*z* [M + H]^+^ calcd. for C_24_H_25_N_6_: 397.2135; found: 397.2134.

*3,6-Bis(3-chlorophenyl)-1,8-dimethyl-3,6-dihydrodipyrazolo [3,4-c:4′,3′-e] pyridazine* (**2g**), (silica gel: 200–300 mesh, solvent system: petroleum ether/ethyl acetate = 5:1), 29 mg, 70%, yellow solid, m.p.: 251–252 °C. ^1^H NMR (400 MHz, CDCl_3_) *δ* (ppm) 8.50–8.44 (m, 4H), 7.50 (t, *J* = 8.0 Hz, 2H), 7.34 (ddd, *J* = 8.1, 2.0, 1.0 Hz, 2H), 2.99 (s, 6H). ^13^C NMR (126 MHz, CDCl_3_) *δ* (ppm) 150.7, 141.0, 140.1, 135.1, 130.3, 126.6, 121.3, 119.2, 110.1, 15.4. HRMS (ESI): *m*/*z* [M + H]^+^ calcd. for C_20_H_15_C_l2_N_6_: 409.0730; found: 409.0723.

*1,8-Dimethyl-3,6-bis(4-(trifluoromethyl)phenyl)-3,6-dihydrodipyrazolo [3,4-c:4′,3′-e] pyridazine* (**2h**), (silica gel: 200–300 mesh, solvent system: petroleum ether/ethyl acetate = 5:1), 28 mg, 58%, white solid, m.p.: 252–253 °C. ^1^H NMR (500 MHz, CDCl_3_) *δ* (ppm) 8.70 (d, *J* = 8.5 Hz, 4H), 7.83 (d, *J* = 8.5 Hz, 4H), 3.01 (s, 6H). ^13^C NMR (126 MHz, CDCl_3_) *δ* (ppm) 150.9, 141.8, 141.5, 126.5 (q, *J_CF_* = 3.7 Hz), 120.8, 110.5, 15.5. ^19^F NMR (470 MHz, CDCl_3_) *δ* (ppm) −62.13. HRMS (ESI): *m*/*z* [M + H]^+^ calcd. for C_22_H_15_F_6_N_6_: 477.1257; found: 477.1249.

*3,6-Bis(2,4-dichlorophenyl)-1,8-dimethyl-3,6-dihydrodipyrazolo [3,4-c:4′,3′-e] pyridazine* (**2i**), (silica gel: 200–300 mesh, solvent system: petroleum ether/ethyl acetate = 5:1), 33 mg, 70%, yellow solid, m.p.: 199–200 °C. ^1^H NMR (500 MHz, CDCl_3_) *δ* (ppm) 7.65 (d, *J* = 2.5 Hz, 2H), 7.59 (d, *J* = 8.5 Hz, 2H), 7.45 (dd, *J* = 8.5, 2.0 Hz, 2H), 3.01 (s, 6H). ^13^C NMR (126 MHz, CDCl_3_) *δ* (ppm) 151.4, 141.5, 135.8, 134.4, 133.0, 130.6, 130.5, 127.9, 108.8, 15.4. HRMS (ESI): *m*/*z* [M + H]^+^ calcd. for C_20_H_13_C_l4_N_6_: 476.9950; found: 476.9943.

*1,3,6,8-Tetramethyl-3,6-dihydrodipyrazolo [3,4-c:4′,3′-e] pyridazine* (**2j**), (silica gel: 200–300 mesh, solvent system: petroleum ether/ethyl acetate = 5:1), 11 mg, 50%, yellow solid, m.p.: 106–107 °C. ^1^H NMR (500 MHz, CDCl_3_) *δ* (ppm) 4.39 (s, 6H), 2.84 (s, 6H). ^13^C NMR (126 MHz, CDCl_3_) *δ* (ppm) 150.6, 138.0, 107.4, 35.1, 14.9. HRMS (ESI): *m*/*z* [M + H]^+^ calcd. for C_10_H_13_N_6_: 217.1196; found: 217.1188.

*1,8-Dimethyl-3,6-di(naphthalen-2-yl)-3,6-dihydrodipyrazolo [3,4-c:4′,3′-e] pyridazine* (**2k**), (silica gel: 200–300 mesh, solvent system: petroleum ether/ethyl acetate = 5:1), 30 mg, 68%, yellow solid, m.p.: 242–243 °C. ^1^H NMR (400 MHz, CDCl_3_) *δ* (ppm) 8.96 (d, *J* = 2.4 Hz, 2H), 8.59 (dd, *J* = 9.2, 2.4 Hz, 2H), 8.05–7.99 (m, 4H), 7.90 (d, *J* = 8.0 Hz, 2H), 7.57–7.49 (m, 4H), 3.01 (s, 6H). ^13^C NMR (126 MHz, CDCl_3_) *δ* 150.8, 140.5, 136.8, 133.6, 131.9, 129.2, 128.4, 127.7, 126.7, 126.0, 120.4, 119.0, 109.8, 15.5. HRMS (ESI): *m*/*z* [M + H]^+^ calcd. for C_28_H_21_N_6_: 441.1822; found: 441.1815.

*1,8-Diethyl-3,6-diphenyl-3,6-dihydrodipyrazolo [3,4-c:4′,3′-e] pyridazine* (**2l**), (silica gel: 200–300 mesh, solvent system: petroleum ether/ethyl acetate = 5:1), 29 mg, 78%, yellow solid, m.p.: 246–247 °C. ^1^H NMR (500 MHz, CDCl_3_) *δ* (ppm) 8.45 (d, *J* = 7.5 Hz, 4H), 7.57 (t, *J* = 7.5 Hz, 4H), 7.37 (t, *J* = 7.5 Hz, 2H), 3.37 (q, *J* = 7.5 Hz, 4H), 1.58 (d, *J* = 7.5 Hz, 6H). ^13^C NMR (126 MHz, CDCl_3_) *δ* 150.5, 145.6, 139.3, 129.2, 126.5, 121.6, 108.9, 22.8, 13.4. HRMS (ESI): *m*/*z* [M + H]^+^ calcd. for C_22_H_21_N_6_: 369.1822; found: 369.1817.

*1,3,6,8-Tetraphenyl-3,6-dihydrodipyrazolo [3,4-c:4′,3′-e] pyridazine* (**2m**), (silica gel: 200–300 mesh, solvent system: petroleum ether/ethyl acetate = 5:1), 35 mg, 76%, orange solid, m.p.: 217–218 °C. ^1^H NMR (400 MHz, CDCl_3_) *δ* (ppm) 8.54–8.50 (m, 4H), 7.64–7.59 (m, 4H), 7.43 (t, *J* = 7.6 Hz, 2H), 7.37 (d, *J* = 6.8 Hz, 4H), 7.21 (t, *J* = 7.6 Hz, 2H), 7.03 (t, *J* = 8.0 Hz, 4H). ^13^C NMR (101 MHz, CDCl_3_) *δ* (ppm) 151.1, 144.7, 139.1, 132.3, 129.3, 128.6, 127.8, 127.2, 122.3, 108.2. HRMS (ESI): *m*/*z* [M + H]^+^ calcd. for C_30_H_21_N_6_: 465.1822; found: 465.1813.

*3,6-Diphenyl-1,8-di(thiophen-2-yl)-3,6-dihydrodipyrazolo [3,4-c:4′,3′-e] pyridazine* (**2n**), (silica gel: 200–300 mesh, solvent system: petroleum ether/ethyl acetate = 5:1), 28 mg, 58%, orange solid, m.p.: 253–354 °C. ^1^H NMR (500 MHz, CDCl_3_) *δ* (ppm) 8.46 (d, *J* = 8.0 Hz, 4H), 7.61 (t, *J* = 8.0 Hz, 4H), 7.44 (t, *J* = 7.5 Hz, 2H), 7.32 (d, *J* = 5.0 Hz, 2H), 6.82 (d, *J* = 3.5 Hz, 2H), 6.80–6.78 (m, 2H). ^13^C NMR (126 MHz, CDCl_3_) *δ* (ppm) 150.8, 138.8, 138.6, 133.0, 129.3, 129.0, 127.5, 127.4, 126.8, 122.5, 108.4. HRMS (ESI): *m*/*z* [M + H]^+^ calcd. for C_26_H_17_N_6_S_2_: 477.0951; found: 477.0945.

*1,8-Bis(4-fluorophenyl)-3,6-diphenyl-3,6-dihydrodipyrazolo [3,4-c:4′,3′-e] pyridazine* (**2o**), (silica gel: 200–300 mesh, solvent system: petroleum ether/ethyl acetate = 5:1), 32 mg, 64%, orange solid, m.p.: 254–255 °C. ^1^H NMR (400 MHz, CDCl_3_) *δ* (ppm) 8.51–8.47 (m, 4H), 7.64–7.59 (m, 4H), 7.47–7.42 (m, 2H), 7.37–7.32 (m, 4H), 6.84–6.77 (m, 4H). ^13^C NMR (126 MHz, CDCl_3_) *δ* (ppm) 164.6, 162.1, 151.0, 143.5, 139.0, 130.4 (d, *J_CF_* = 8.3 Hz), 128.4, 127.4, 122.3, 115.0, 114.9 (d, *J_CF_* = 21.9 Hz), 108.0. ^19^F NMR (470 MHz, CDCl_3_) *δ* (ppm) −112.51. HRMS (ESI): *m*/*z* [M + H]^+^ calcd. for C_30_H_19_F_2_N_6_: 501.1634; found: 501.1626.

*1,8-Bis(4-fluorophenyl)-3,6-di-p-tolyl-3,6-dihydrodipyrazolo [3,4-c:4′,3′-e] pyridazine* (**2p**), (silica gel: 200–300 mesh, solvent system: petroleum ether/ethyl acetate = 5:1), 32 mg, 60%, orange solid, m.p.: 253–254 °C. ^1^H NMR (500 MHz, CDCl_3_) *δ* (ppm) 8.33 (d, *J* = 8.5 Hz, 4H), 7.40 (d, *J* = 8.5 Hz, 4H), 7.33 (dd, *J* = 8.5, 5.5 Hz, 4H), 6.79 (t, *J* = 8.5 Hz, 4H), 2.47 (s, 6H). ^13^C NMR (126 MHz, CDCl_3_) δ (ppm) 164.3, 162.3, 150.8, 143.1, 137.3, 136.6, 130.4 (d, *J_CF_* = 8.3 Hz), 128.5 (d, *J_CF_* = 3.2 Hz), 122.2, 115.0, 114.8 (d, *J_CF_* = 22.0 Hz), 107.8, 21.1. ^19^F NMR (470 MHz, CDCl_3_) *δ* (ppm) −112.67. HRMS (ESI): *m*/*z* [M + H]^+^ calcd. for C_32_H_23_F_2_N_6_: 529.1947; found: 529.1939.

*3,7-Dimethyl-1,5-diphenyl-1,5-dihydrodipyrazolo [3,4-b:3′,4′-e] pyrazine* (**3a**), (silica gel: 200–300 mesh, solvent system: petroleum ether/ethyl acetate = 10:1), 20 mg,59%, orange solid, m.p.: 276–277 °C. ^1^H NMR (500 MHz, CDCl_3_) *δ* (ppm) 8.40 (d, *J* = 8.0 Hz, 4H), 7.57 (t, *J* = 8.0 Hz, 4H), 7.32 (t, *J* = 7.5 Hz, 2H), 2.84 (s, 6H). ^13^C NMR (126 MHz, CDCl_3_) *δ* (ppm) 143.1, 142.3, 139.5, 134.1, 129.2, 125.6, 119.8, 11.6. HRMS (ESI): *m*/*z* [M + H]^+^ calcd. for C_20_H^17^N_6_: 341.1509; found: 341.1501.

*3,7-Dimethyl-1,5-di-o-tolyl-1,5-dihydrodipyrazolo [3,4-b:3′,4′-e] pyrazine* (**3b**), (silica gel: 200–300 mesh, solvent system: petroleum ether/ethyl acetate = 10:1), 19 mg, 52%, yellow solid, m.p.: 234–235 °C. ^1^H NMR (400 MHz, CDCl_3_) *δ* (ppm) 7.50–7.47 (m, 2H), 7.44 (td, *J* = 7.1, 6.6, 2.1 Hz, 4H), 7.41–7.36 (m, 2H), 2.74 (s, 6H), 2.28 (s, 6H). ^13^C NMR (101 MHz, CDCl_3_) *δ* (ppm) 143.3, 142.6, 137.3, 135.5, 133.3, 131.4, 128.7, 127.7, 126.7, 18.7, 11.7. HRMS (ESI): *m*/*z* [M + H]^+^ calcd. for C_22_H_21_N_6_: 369.1822; found: 369.1816.

*3,7-Dimethyl-1,5-di-p-tolyl-1,5-dihydrodipyrazolo [3,4-b:3′,4′-e] pyrazine* (**3c**), (silica gel: 200–300 mesh, solvent system: petroleum ether/ethyl acetate = 10:1), 17 mg, 46%, yellow solid, m.p.: 273–274 °C. Due to the low solubility of the product, 0.6 mL of CDCl_3_/TFA (v:v = 20:1) was used to measure the NMR spectrum. ^1^H NMR ((400 MHz, CDCl_3_/TFA = 20:1(v:v)) *δ* (ppm) 7.71 (d, *J* = 8.4 Hz, 4H), 7.43 (d, *J* = 8.0 Hz, 4H), 2.87 (s, 6H), 2.48 (s, 6H). ^13^C NMR (101 MHz, CDCl_3_/TFA = 20:1(v:v)) *δ* (ppm) 162.3 (q, *J*_CF_ = 43.8 Hz, CO), 143.40, 142.11, 139.87, 133.84, 133.62, 130.53, 124.03, 114.3 (q, *J*_CF_ = 285.6 Hz, CF_3_), 20.95, 10.76. HRMS (ESI): *m*/*z* [M + H]^+^ calcd. for C_17_H_14_NO_5_: 369.1822; found: 369.1813.

*1,5-Bis(4-isopropylphenyl)-3,7-dimethyl-1,5-dihydrodipyrazolo [3,4-b:3′,4′-e] pyrazine* (**3d**), (silica gel: 200–300 mesh, solvent system: petroleum ether/ethyl acetate = 10:1), 19 mg, 45%, white solid, m.p.: 236–237 °C. ^1^H NMR (400 MHz, CDCl_3_) *δ* (ppm) 8.26 (d, *J* = 8.6 Hz, 4H), 7.42 (d, *J* = 8.6 Hz, 4H), 3.00 (m, 2H), 2.82 (s, 6H), 1.33 (d, *J* = 6.9 Hz, 12H). ^13^C NMR (101 MHz, CDCl_3_) *δ* 146.3, 142.6, 142.1, 137.3, 133.9, 127.2, 120.0, 33.8, 24.1, 11.6. HRMS (ESI): *m*/*z* [M + H]^+^ calcd. for C_26_H_29_N_6_: 425.2448; found: 425.2441.

*1,5-Bis(3,4-dimethylphenyl)-3,7-dimethyl-1,5-dihydrodipyrazolo [3,4-b:3′,4′-e] pyrazine* (**3e**), (silica gel: 200–300 mesh, solvent system: petroleum ether/ethyl acetate = 10:1), 23 mg, 59%, yellow solid, m.p.: 251–252 °C. Due to the low solubility of the product, 0.6 mL of CDCl3/TFA (v:v = 20:1) was used to measure the NMR spectrum. ^1^H NMR (500 MHz, CDCl_3_/TFA = 20:1(v:v)) *δ* (ppm) 7.55 (d, *J* = 8.8 Hz, 1H), 7.37 (d, *J* = 8.4 Hz, 1H), 2.87 (s, 1H), 2.38 (s, 3H). ^13^C NMR (126 MHz, CDCl_3_/TFA = 20:1(v:v)) *δ* (ppm) 162.2 (q, *J*_CF_ = 44.1 Hz, CO), 143.1, 142.0, 138.8, 138.5, 133.7, 130.9, 125.1, 121.5, 114.3 (q, *J*_CF_ = 285.4 Hz, CF3), 19.7, 19.4, 10.8. HRMS (ESI): *m*/*z* [M + H]^+^ calcd. for C_24_H_25_N_6_: 397.2135; found: 397.2125.

*1,5-Bis(3,5-dimethylphenyl)-3,7-dimethyl-1,5-dihydrodipyrazolo [3,4-b:3′,4′-e] pyrazine* (**3f**), (silica gel: 200–300 mesh, solvent system: petroleum ether/ethyl acetate = 10:1), 21 mg, 52%, yellow solid, m.p.: 254–255 °C. ^1^H NMR (400 MHz, CDCl_3_) *δ* (ppm) 7.98 (s, 4H), 6.97 (s, 2H), 2.83 (s, 6H), 2.47 (s, 12H). ^13^C NMR (101 MHz, CDCl_3_) *δ* (ppm) 153.3, 142.7, 142.2, 139.3, 139.0, 134.0, 127.4, 117.9, 21.6, 11.6. HRMS (ESI): *m*/*z* [M + H]^+^ calcd. for C_24_H_25_N_6_: 397.2135; found: 397.2123.

*Methyl 6-chloro-9-oxo-3,3a-dihydro-9H-chromeno [3,2-c]isoxazole-3-carboxylate* (**3g**), (silica gel: 200–300 mesh, solvent system: petroleum ether/ethyl acetate = 10:1), 16 mg, 40%, yellow solid, m.p.: 238–239 °C. ^1^H NMR (500 MHz, CDCl_3_) *δ* (ppm) 8.47 (s, 2H), 8.39 (d, *J* = 8.5 Hz, 2H), 7.49 (t, *J* = 8.0 Hz, 2H), 7.28 (d, *J* = 10.0 Hz, 2H), 2.84 (s, 6H). ^13^C NMR (126 MHz, CDCl_3_) *δ* (ppm) 143.8, 142.3, 140.4, 135.0, 134.2, 130.3, 125.5, 119.5, 117.3, 11.6. HRMS (ESI): *m*/*z* [M + H]^+^ calcd. for C_20_H_15_Cl_2_N_6_: 397.2135; found: 397.2130.

*3,7-Dimethyl-1,5-bis(4-(trifluoromethyl)phenyl)-1,5-dihydrodipyrazolo [3,4-b:3′,4′-e] pyrazine* (**3h**), (silica gel: 200–300 mesh, solvent system: petroleum ether/ethyl acetate = 10:1), 26 mg, 54%, yellow solid, m.p.: 252–253 °C. Due to the low solubility of the product, 0.6 mL of CDCl_3_/TFA (v:v = 20:1) was used to measure the NMR spectrum. ^1^H NMR (500 MHz, CDCl_3_/TFA = 20:1 (v:v)) *δ* (ppm) 8.34 (d, *J* = 8.0 Hz, 4H), 7.88 (d, *J* = 8.5 Hz, 4H), 2.90 (s, 6H). ^13^C NMR (126 MHz, CDCl_3_/TFA = 20:1 (v:v)) *δ* (ppm) 162.2 (q, *J*_CF_ = 44.0 Hz, CO), 145.1, 142.6, 140.6, 134.5, 126.9 (q, *J_CF_* = 3.7 Hz), 121.2, 114.2(q, *J*_CF_ = 284.8 Hz, CF_3_), 11.2. ^19^F NMR (470 MHz, CDCl_3_) *δ* (ppm)-62.13. HRMS (ESI): *m*/*z* [M + H]+ calcd. for C22H15F6N6: 477.1257; found: 477.1249.

*1,5-Bis(2,4-dichlorophenyl)-3,7-dimethyl-1,5-dihydrodipyrazolo [3,4-b:3′,4′-e] pyrazine* (**3i**), (silica gel: 200–300 mesh, solvent system: petroleum ether/ethyl acetate = 10:1), 22 mg, 46%, light yellow solid, m.p.: 256–257 °C. Due to the low solubility of the product, 0.6 mL of CDCl_3_/TFA (v:v = 20:1) was used to measure the NMR spectrum. ^1^H NMR (500 MHz, CDCl_3_/TFA = 20:1 (v:v)) *δ* (ppm) 7.69 (d, *J* = 2.5 Hz, 2H), 7.55 (d, *J* = 8.0 Hz, 2H), 7.51 (dd, *J* = 8.5, 2.5 Hz, 2H), 2.80 (s, 6H). ^13^C NMR (126 MHz, CDCl_3_/TFA = 20:1 (v:v)) *δ* (ppm) 161.7 (q, *J*_CF_ = 43.5Hz, CO), 144.5, 143.1, 137.4, 133.8, 133.4, 132.3, 130.8, 130.8, 128.4, 114.2 (q, *J*_CF_ = 284.8Hz, CF3). HRMS (ESI): *m*/*z* [M + H]^+^ calcd. for C_20_H_13_Cl_4_N_6_: 476.9950; found: 476.9945.

*1,3,5,7-Tetramethyl-1,5-dihydrodipyrazolo [3,4-b:3′,4′-e] pyrazine* (**3j**), (silica gel: 200–300 mesh, solvent system: petroleum ether/ethyl acetate = 10:1), 12 mg, 57%, yellow solid, m.p.: 216–217 °C. ^1^H NMR (500 MHz, CDCl_3_) *δ* (ppm) 4.16 (s, 6H), 2.72 (s, 6H). ^13^C NMR (126 MHz, CDCl_3_) *δ* (ppm) 142.9, 140.1, 132.6, 34.1, 11.5. HRMS (ESI): *m*/*z* [M + H]^+^ calcd. for C_10_H_13_N_6_: 217.1196; found: 217.1190.

*3,7-Dimethyl-1,5-di(naphthalen-2-yl)-1,5-dihydrodipyrazolo [3,4-b:3′,4′-e] pyrazine* (**3k**), (silica gel: 200–300 mesh, solvent system: petroleum ether/ethyl acetate = 10:1), 26 mg, 60%, yellow solid, m.p.: 252–253 °C. Due to the low solubility of the product, 0.6 mL of CDCl_3_/TFA (v:v = 20:1) was used to measure the NMR spectrum. ^1^H NMR (500 MHz, CDCl_3_/TFA = 20:1 (v:v)) *δ* 8.45 (s, 2H), 8.10 (d, *J* = 1.5 Hz, 4H), 7.97 (ddd, *J* = 12.0, 7.6, 2.0 Hz, 4H), 7.61 (m, 4H), 2.93 (s, 6H). ^13^C NMR (126 MHz, CDCl_3_/TFA = 20:1 (v:v)) *δ* 162.1 (q, *J*_CF_ = 42.8 Hz, CO), 144.01, 142.41, 134.29, 134.13, 133.45, 132.68, 129.99, 128.27, 128.01, 127.45, 127.09, 121.35, 120.98, 114.2 (q, *J*_CF_ = 284.8 Hz, CF_3_), 11.01. HRMS (ESI): *m*/*z* [M + H]^+^ calcd. for C_28_H_21_N_6_: 441.1822; found: 441.1816.

*1,5-Diphenyl-3,7-dipropyl-1,5-dihydrodipyrazolo [3,4-b:3′,4′-e] pyrazine* (**3l**), (silica gel: 200–300 mesh, solvent system: petroleum ether/ethyl acetate = 10:1), 24 mg, 60%, yellow solid, m.p.: 228–229 °C. 1H NMR (500 MHz, CDCl_3_) δ (ppm) 8.42 (d, *J* = 7.4 Hz, 4H), 7.59–7.55 (m, 4H), 7.31 (t, *J* = 7.5 Hz, 2H), 3.23 (t, *J* = 7.5 Hz, 4H), 2.07 (m, 4H), 1.12 (t, *J* = 7.5 Hz, 6H). ^13^C NMR (126 MHz, CDCl_3_) δ (ppm) 146.8, 142.3, 139.6, 133.8, 129.2, 125.4, 119.7, 28.5, 21.6, 14.1. HRMS (ESI): *m*/*z* [M + H]+ calcd. for C_24_H_25_N_6_: 397.2135; found: 397.2127.

*1,3,5,7-Tetraphenyl-1,5-dihydrodipyrazolo [3,4-b:3′,4′-e] pyrazine* (**3m**), (silica gel: 200–300 mesh, solvent system: petroleum ether/ethyl acetate = 10:1), 27 mg, 58%, orange solid, m.p.: 275–276 °C. Due to the low solubility of the product, 0.6 mL of CDCl_3_/TFA (v:v = 20:1) was used to measure the NMR spectrum. ^1^H NMR (400 MHz, CDCl_3_/TFA = 20:1 (v:v)) *δ* (ppm) 8.45 (d, *J* = 6.4 Hz, 4H), 8.17 (d, *J* = 7.6 Hz, 4H), 7.71–7.51 (m, 12H). ^13^C NMR (101 MHz, CDCl_3_/TFA = 20:1 (v:v)) *δ* (ppm) 162.7 (q, *J*_CF_ = 43.4 Hz, CO), 158.1, 137.3, 130.8, 129.9, 129.5, 128.6, 128.1, 123.2, 114.4 (q, *J*_CF_ = 285.8 Hz, CF_3_). HRMS (ESI): *m*/*z* [M + H]^+^ calcd. for C_30_H_21_N_6_: 465.1822; found: 465.1816.

*1,5-Di-tert-butyl-3,7-dimethyl-1,5-dihydrodipyrazolo [3,4-b:3′,4′-e] pyrazine* (**3q**), (silica gel: 200–300 mesh, solvent system: petroleum ether/ethyl acetate = 10:1), 12 mg, 40%, yellow solid, m.p.: 228–229 °C. ^1^H NMR (400 MHz, CDCl_3_) *δ* (ppm) 2.68 (s, 6H), 1.85 (s, 18H). ^13^C NMR (101 MHz, CDCl_3_) *δ* (ppm) 142.3, 138.8, 132.7, 59.9, 29.1, 11.3. HRMS (ESI): *m*/*z* [M + H]^+^ calcd. for C_16_H_25_N_6_: 301.2135; found: 301.2134.

*3,7-Di-tert-butyl-1,5-diphenyl-1,5-dihydrodipyrazolo [3,4-b:3′,4′-e] pyrazine* (**3r**), (silica gel: 200–300 mesh, solvent system: petroleum ether/ethyl acetate = 10:1), 19 mg, 45%,yellow solid, m.p.: 252–253 °C. ^1^H NMR (400 MHz, CDCl_3_) *δ* (ppm) 8.49–8.44 (m, 4H), 7.59–7.54 (m, 4H), 7.29 (m, 2H), 1.72 (s, 18H). ^13^C NMR (101 MHz, CDCl_3_) *δ* (ppm) 153.1, 142.0, 139.9, 132.4, 129.1, 125.1, 119.4, 34.3, 29.2. HRMS (ESI): *m*/*z* [M + H]^+^ calcd. for C_26_H_29_N_6_: 425.2448; found: 425.2441.

*3,7-Di-tert-butyl-1,5-bis(3,5-dimethylphenyl)-1,5-dihydrodipyrazolo [3,4-b:3′,4′-e] pyrazine* (**3s**), (silica gel: 200–300 mesh, solvent system: petroleum ether/ethyl acetate = 10:1), 20 mg, 42%, yellow solid, m.p.: 262–263 °C. ^1^H NMR (500 MHz, CDCl_3_) *δ* (ppm) 8.10 (s, 4H), 6.94 (s, 2H), 2.47 (s, 12H), 1.72 (s, 18H). ^13^C NMR (126 MHz, CDCl_3_) *δ* (ppm) 152.8, 141.9, 139.7, 138.8, 132.2, 126.8, 117.3, 34.2, 29.1, 21.7. HRMS (ESI): *m*/*z* [M + H]^+^ calcd. for C_30_H_37_N_6_: 481.3074; found: 481.3070.

*(E)-1,2-bis(3-methyl-1-phenyl-1H-pyrazol-5-yl)diazene* (**4a**), (silica gel: 200–300 mesh, solvent system: petroleum ether/ethyl acetate = 8:1), 14 mg, 40%, orange solid, m.p.: 198–199 °C. ^1^H NMR (500 MHz, CDCl_3_) *δ* (ppm) 7.70 (d, *J* = 7.5 Hz, 4H), 7.50 (t, *J* = 7.5 Hz, 4H), 7.40 (t, *J* = 7.5 Hz, 2H), 6.40 (s, 2H), 2.39 (s, 6H). ^13^C NMR (126 MHz, CDCl_3_) *δ* (ppm) 154.3, 150.0, 139.0, 128.8, 127.5, 124.8, 94.6, 14.0. HRMS (ESI): *m*/*z* [M + H]^+^ calcd. for C_20_H_19_N_6_: 343.1666; found: 343.1663.

*(1-Cyclopropylvinyl)benzene* (**5**), (silica gel: 200–300 mesh, solvent system: petroleum ether), 20 mg, 70%, white solid. ^1^H NMR (500 MHz, CDCl_3_) *δ* (ppm) 7.58 (d, *J* = 6.0 Hz, 2H), 7.31 (t, *J* = 7.0 Hz, 2H), 7.26 (d, *J* = 7.5 Hz, 1H), 5.26 (s, 1H), 4.92 (s, 1H), 1.63 (m, 1H), 0.83–0.79 (m, 2H), 0.58 (dd, *J* = 5.5, 2.2 Hz, 2H). ^13^C NMR (126 MHz, CDCl_3_) *δ* (ppm) 149.3, 141.6, 128.1, 127.4, 126.1, 109.0, 15.6, 6.6. HRMS (ESI): *m*/*z* [M + H]^+^ calcd. for C_11_H_13_: 145.1012; found: 145.1008.

## 4. Conclusions

In summary, we have developed a concise protocol for the synthesis of dipyrazole-fused pyridazines and pyrazines via C-H/C-H, C-H/N-H, and N-H/N-H direct coupling from simple 5-aminopyrazoles. The mechanism investigations uncovered that the reaction involved the radical dimerization of 5-aminopyrazoles. The protocol exhibits broad substrate scope and high functional group compatibility. In addition, the preliminary fluorescence results suggest that dipyrazole-fused pyridazines and pyrazines may have some potential applications in materials chemistry.

## Figures and Tables

**Table 1:** Optimization studies for the preparation of **2a** ^a,b^. (^a^) Reaction conditions: 3-methyl-1-phenyl-1*H*-pyrazol-5-amine **1a** (0.2 mmol) in solvent (2.0 mL) at different temperatures for 10 h under air in closed vial. (^b^) Isolated yields of **2a** based on **1a**. BPO = benzoyl peroxide, TBHP = tert-butyl hydroperoxide, DDQ = 2,3-dichloro-5,6-dicyano-1,4-benzoquinone, TBPB = tert-butyl peroxy benzoate.

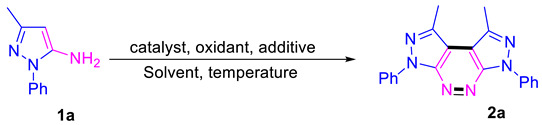						
**Entry**	**Catalysts ** **(Equiv.)**	**Oxidants ** **(Equiv.)**	**Additives** **(Equiv.)**	**Temp ** **(℃)**	**Solvent**	**Yield (%)**
1	Cu(OAc)_2_ (3.0)			100	Toluene	50
2	Cu(OAc)_2_ (3.0)	BPO (0.5)		100	Toluene	58
3	Cu(OAc)_2_ (3.0)	TBHP (0.5)		100	Toluene	52
4	Cu(OAc)_2_ (3.0)	DDQ (0.5)		100	Toluene	42
5	Cu(OAc)_2_ (3.0)	TBPB (0.5)		100	Toluene	38
6	CuCl_2_(3.0)	BPO (0.5)		100	Toluene	46
7	CuBr (3.0)	BPO (0.5)		100	Toluene	40
8	CuI (3.0)	BPO (0.5)		100	Toluene	38
9	CuCl (3.0)	BPO (0.5)		100	Toluene	42
10	Cu(OAc)_2_.H_2_O (3.0)	BPO (0.5)		100	Toluene	48
11	Zn (OAc)_2_ (3.0)	BPO (0.5)		100	Toluene	36
12	FeCl_3_(3.0)	BPO (0.5)		100	Toluene	12
13	Cu(OAc)_2_ (3.0)	BPO (0.5)		110	Toluene	55
14	Cu(OAc)_2_ (3.0)	BPO (0.5)		120	Toluene	53
15	Cu(OAc)_2_ (3.0)	BPO (0.5)		90	Toluene	51
16	Cu(OAc)_2_ (3.0)	BPO (0.5)		80	Toluene	44
17	Cu(OAc)_2_ (2.0)	BPO (0.5)		100	Toluene	56
18	Cu(OAc)_2_ (1.0)	BPO (0.5)		100	Toluene	48
19	Cu(OAc)_2_ (3.0)	BPO (0.25)		100	Toluene	55
20	Cu(OAc)_2_ (3.0)	BPO (0.75)		100	Toluene	53
21	Cu(OAc)_2_ (3.0)	BPO (0.5)		100	DMSO	50
22	Cu(OAc)_2_ (3.0)	BPO (0.5)		100	Dioxane	40
23	Cu(OAc)_2_ (3.0)	BPO (0.5)		100	DEC	42
24	Cu(OAc)_2_ (3.0)	BPO (0.5)		100	MeCN	40
25	Cu(OAc)_2_ (3.0)	BPO (0.5)		100	DMF	56
26	Cu(OAc)_2_ (3.0)	BPO (0.5)	Na_2_CO_3_ (2.5)	100	Toluene	54
27	Cu(OAc)_2_ (3.0)	BPO (0.5)	Cs_2_CO_3_ (2.5)	100	Toluene	41
28	Cu(OAc)_2_ (3.0)	BPO (0.5)	K_2_S_2_O_8_ (2.5)	100	Toluene	79
29	Cu(OAc)_2_ (3.0)	BPO (0.5)	K_2_S_2_O_8_ (1.5)	100	Toluene	60
30	Cu(OAc)_2_ (3.0)	BPO (0.5)	K_2_S_2_O_8_ (3.5)	100	Toluene	70

**Table 2:** Optimization studies for the preparation of **3a** ^a,b^. (^a^) Reaction conditions: 3-methyl-1-phenyl-1*H*-pyrazol-5-amine **1a** (0.2 mmol) in solvent (2.0 mL) at different temperatures for 12 h under air in closed vial. (^b^) Isolated yields. BPO = benzoyl peroxide, TBHP = tert-butyl hydroperoxide, DDQ = 2,3-dichloro-5,6-dicyano-1,4-benzoquinone, TBPB = tert-butyl peroxy benzoate, Bipy = 2,2-bipyridine, 1,10-phen = 1,10-phenanthroline.

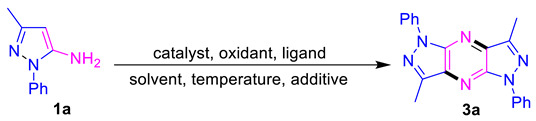							
**Entry**	**Catalysts ** **(Equiv.)**	**Oxidants ** **(Equiv.)**	**Additives ** **(Equiv.)**	**Ligands ** **(Equiv.)**	**Temp ** **(℃)**	**Solvent**	**Yield** **(%)**
1	Cu(OAc)_2_ (20%)				130	Toluene	36
2	Cu(OAc)_2_ (20%)	BPO (0.5)			130	Toluene	38
3	Cu(OAc)_2_ (20%)	TBHP (0.5)			130	Toluene	37
4	Cu(OAc)_2_ (20%)	DDQ (0.5)			130	Toluene	32
5	Cu(OAc)_2_ (20%)	TBPB (0.5)			130	Toluene	41
6	CuCl_2_ (20%)	TBPB (0.5)			130	Toluene	47
7	CuBr (20%)	TBPB (0.5)			130	Toluene	40
8	CuI (20%)	TBPB (0.5)			130	Toluene	38
9	CuCl (20%)	TBPB (0.5)			130	Toluene	41
10	Cu(OAc)_2_.H_2_O(20%)	TBPB (0.5)			130	Toluene	43
11	CuCl_2_ (20%)	TBPB (0.5)	Na_2_CO_3_ (2.5)		130	Toluene	51
12	CuCl_2_ (20%)	TBPB (0.5)	Cs_2_CO_3_ (2.5)		130	Toluene	34
13	CuCl_2_ (20%)	TBPB (0.5)	tBuONa (2.5)		130	Toluene	36
14	CuCl_2_ (20%)	TBPB (0.5)	KI (2.5)		130	Toluene	29
15	CuCl_2_ (20%)	TBPB (0.5)	K_2_S_2_O_8_ (2.5)		130	Toluene	38
16	CuCl_2_ (20%)	TBPB (0.5)	Na_2_CO_3_ (2.5)		130	Toluene	48
17	CuCl_2_ (20%)	TBPB (0.5)	Na_2_CO_3_ (2.5)		130	DMF	35
18	CuCl_2_ (20%)	TBPB (0.5)	Na_2_CO_3_ (2.5)		130	DMSO	32
19	CuCl_2_ (20%)	TBPB (0.5)	Na_2_CO_3_ (2.5)		130	NMP	24
20	CuCl_2_ (20%)	TBPB (0.5)	Na_2_CO_3_ (2.5)		130	Dioxane	26
21	CuCl_2_ (20%)	TBPB (0.5)	Na_2_CO_3_ (2.5)		130	DEC	24
22	CuCl_2_ (20%)	TBPB (0.5)	Na_2_CO_3_ (2.5)		130	DMAC	21
23	CuCl_2_ (20%)	TBPB (0.5)	Na_2_CO_3_ (2.5)	Bipy (0.3)	130	Toluene	49
24	CuCl_2_ (20%)	TBPB (0.5)	Na_2_CO_3_ (2.5)	1,10-Phen (0.3)	130	Toluene	59
25	CuCl_2_ (20%)	TBPB (0.5)	Na_2_CO_3_ (2.5)	1,10-Phen (0.3)	120	Toluene	46
26	CuCl_2_ (20%)	TBPB (0.5)	Na_2_CO_3_ (2.5)	1,10-Phen (0.3)	100	Toluene	38
27	CuCl_2_ (20%)	TBPB (0.5)	Na_2_CO_3_ (2.5)	1,10-Phen (0.3)	80	Toluene	29
28	CuCl_2_ (40%)	TBPB (0.5)	Na_2_CO_3_ (2.5)	1,10-Phen (0.3)	130	Toluene	50
29	CuCl_2_ (10%)	TBPB (0.5)	Na_2_CO_3_ (2.5)	1,10-Phen (0.3)	130	Toluene	35

**Table 3:** Photophysical properties of compound **2** in CH_2_Cl_2_ (1 × 10^−5^ M). ^a^ Absorption maximum. ^b^ Emission maximum. ^c^ Quinine sulfate was used as a standard for fluorescence QY = 0.54 (0.5 M H_2_SO_4_).

Compd.	λ_max_^a^ (nm)	λ_em_^b^ (nm)	Stokes Shift (nm)	Φ_F_^c^ (%)
**2a**	279	502	223	3.0
**2b**	266	298	32	5.2
**2c**	272	295	23	21.8
**2d**	273	300	27	10.3
**2e**	238	304	66	2.2
**2f**	274	296	22	9.0
**2g**	309	492	183	7.4
**2h**	312	478	166	7.6
**2i**	271	294	23	11.6
**2j**	273	300	27	9.1
**2k**	273	293	20	2.0
**2l**	271	294	23	12.6
**2m**	273	511	238	8.0
**2n**	272	296	24	7.5
**2o**	277	510	233	5.8
**2p**	274	298	24	2.0

**Table 4:** Photophysical properties of compound **3** in CH_2_Cl_2_ (1 × 10^−5^ M). ^a^ Absorption maximum. ^b^ Emission maximum. ^c^ Quinine sulfate was used as a standard for fluorescence QY = 0.54 (0.5 M H_2_SO_4_).

Compd.	λ_max_^a^ (nm)	λ_em_^b^ (nm)	Stokes Shift (nm)	Φ_F_^c^ (%)
**3a**	284	505	221	14.0
**3b**	304	501	197	8.6
**3c**	284	523	239	5.3
**3d**	286	530	244	2.4
**3e**	285	530	245	1.7
**3f**	285	522	237	2.7
**3g**	285	495	210	13.8
**3h**	284	496	212	4.3
**3i**	287	474	187	9.2
**3j**	274	302	28	6.2
**3k**	287	544	257	0.3
**3l**	284	503	219	12.3
**3m**	289	534	245	8.0
**3q**	284	499	215	14.3
**3r**	283	500	217	16.2
**3s**	285	512	227	5.9

## Data Availability

The data presented in this study are available in the article and Supplementary Materials.

## Supplementary Materials

The following supporting information can be downloaded at https://www.mdpi.com/article/10.3390/molecules30020381/s1. Tables S1–S7, Scheme S1–S12 and Figures S1–S14: X-ray crystal structure and crystallographic data; optimization of reaction conditions; and characterization data for product **2a**–**2p**, **3a**–**3m**, **3q**–**3s, 4a**, and **5**, including ^1^H-NMR and ^13^C-NMR, see references [49,50,51].
