# Supplementary material for: 5-Aminopyrazole Dimerization: Cu-Promoted Switchable Synthesis of Pyrazole-Fused Pyridazines and Pyrazines via Direct Coupling of C-H/N-H, C-H/C-H, and N-H/N-H Bonds
Source: Molecules. 2025 Jan 17;30(2):381. doi: 10.3390/molecules30020381 (PMC11767409; doi:10.3390/molecules30020381)
Supplement: Supplementary file 1 [file molecules-30-00381-s001.zip › molecules-3417133-supplementary.pdf]

Supporting Information

5-Aminopyrazole Dimerization: Cu-Promoted Switchable Synthesis of Pyrazole-Fused Pyridazines and Pyrazines via Direct Coupling of C-H/N-H, C-H/C-H, and N-H/N-H Bonds

Yi-Xin Chai [1], Jun-Jie Ren [1], Yi-Ming Li [1], Yi-Cheng Bai [1], Qing-Qing Zhang [1], Yi-Zhen Zhao [1], Xue Yang [1], Xiao-Han Zhang [1], Xin-Shuang Zhang [1], An-Xin Wu [3,*], Yan-Ping Zhu [1,2,*] and Yuan-Yuan Sun [1,*]

[1] Key Laboratory of Molecular Pharmacology and Drug Evaluation, Ministry of Education, School of Pharmacy, Yantai University, Yantai 264005, China
[2] International Innovation Center for Forest Chemicals and Materials, Nanjing Forestry University, Nanjing 210037, China
[3] National Key Laboratory of Green Pesticide, International Joint Research Center for Intelligent Biosensor Technology and Health, College of Chemistry, Central China Normal University, Wuhan 430079, China
* Correspondence: chwuax@mail.ccnu.edu.cn (A.-X.W.); chemzyp@ytu.edu.cn (Y.-P.Z.); graceyuanyuan2022@163.com (Y.-Y.S.)

Table of Contents

1. General information **S3**

2. Optimization of the Reaction Conditions **S3**

3. X-Ray Crystal Structure and Crystallographic Data **S6**

4. Fluorescence spectra of compounds 2g and 3m in different solvents **S12**

5.Experimental Procedures **S14**

6. Controlled Experiments **S16**

7. Characterization of Products **S25**

8. References **S41**

9. Copy of ^{1}H, ^{13}C NMR Spectra of Products **S41**

1. General information

Materials and General Experimental: Aminopyrazoles used in the text were purchased from Shanghai Shaoyuan Co. Ltd., all solvents and commercially available reagents were obtained from commercial suppliers and used without further purification Unless stated otherwise, all solvents and commercially available reagents were obtained from commercial suppliers and used without further purification. In addition, petroleumether (b.p. 60-90 °C), which was used for column chromatography, was distilled prior to use. Non-commercial starting materials were prepared as described below or according to literature procedures. Analytical thin layer chromatography (TLC) was performed using pre-coated silica gel HF254 glass plates. Column chromatography was performed using silica gel (200-300 mesh).

Instrumentation: Nuclear magnetic resonance (NMR) spectra were recorded on a Bruker Advance 500 or 400 MHz spectrometers at ambient temperature using the non or partly deuterated solvent as internal standard (^{1}H: δ 7.26 ppm and $^{13}C\{^1H\}$: *δ* 77.0 ppm for $CDCl_3$). Chemical shifts (*δ*) are reported in ppm, relative to the internal standard of tetramethylsilane (TMS). The coupling constants (*J*) are quoted in hertz (Hz). Resonances are described as s (singlet), d (doublet), t (triplet), q (quartet), m (multiplet), br (broad), or combinations thereof. High-resolution mass-spectrometric (HRMS) were obtained on an Apex-Ultra MS equipped with an electrospray source. Melting points were determined using SGW X-4 apparatus and not corrected. The X-ray diffraction data for the crystallized compound were collected on a Bruker Smart APEX CCD area detector diffractometer (graphite monochromator, Mo Kα radiation, λ = 0.71073 Å) at 296(2) K. All the heating procedures were conducted with an oil bath. UV data was recorded by Shimadzu UV 2000 at ambient temperature and fluorescence data was recorded by HITACHI F7000 at ambient temperature.

2. Optimization of the Reaction Conditions

3-Methyl-1-phenyl-1*H*-pyrazol-5-amine **1a** was selected as model substrate for screening and optimization of the reaction conditions. Under the initial conditions shown in entry 1 of Table S1 for **1a** (0.2 mmol) using $Cu(OAc)_2$ as a catalyst, the desired product **2a** was obtained with a yield of 50%. Subsequently, we investigated the effect of the type of oxidants on the reaction, e.g., benzoyl peroxide, tert-butyl hydroperoxide, 2,3-dichloro-5,6-dicyano-1,4-benzoquinone and tert-butyl peroxybenzo, it was found that when benzoyl peroxide was used as oxidant, a yield of 58% was achieved (Table S1, entries 2–5). Then, the different catalyst types were studied, and it was found that the yields were all reduced (Table S1, entries 6–12). The effect of temperature on the method was also tested. These results showed that the optimal reaction temperature was 100 °C (Table S1, entries 13–16). Continuing the study of the equivalent of $Cu(OAc)_2$, it was found that the yield was decreased when the amount of $Cu(OAc)_2$ was decreased (Table S1, entries 17–18). Benzoyl peroxide equivalents were also tested and the best yield was found at 0.5 equivalent (Table S1, entries 19–20). The solvents were also investigated, such as DMSO, 1,4-dioxane,

dimethyl carbonate, acetonitrile, and DMF, the yields have not improved well (Table S1, entries 21–25). Finally, the effect of adding three different additives, Na_2CO_3, Cs_2CO_3, $K_2S_2O_8$, were tested and it was found that the addition of $K_2S_2O_8$, resulted in a significant increase in yield (Table S1, entries 26–28). Following this, the equiv.alent of $K_2S_2O_8$ was investigated and it was found that the highest yield was achieved when the equivalent of $K_2S_2O_8$ was 2.5 (Table S1, entries 29–30).

Table S1. Optimization studies for the preparation of **2a** [a, b]

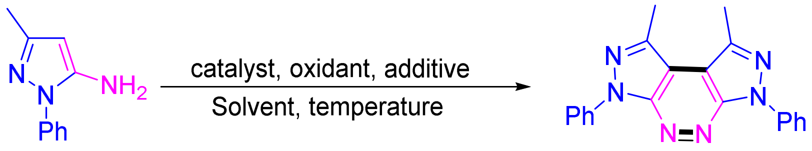

1a **2a**

Entry	Catalysts (equiv.)	Oxidants (equiv.)	Additives (equiv.)	Temp (°C)	Solvent	Yield (%)
1	$Cu(OAc)_2$ (3.0)			100	Toluene	50
2	$Cu(OAc)_2$ (3.0)	BPO (0.5)		100	Toluene	58
3	$Cu(OAc)_2$ (3.0)	TBHP (0.5)		100	Toluene	52
4	$Cu(OAc)_2$ (3.0)	DDQ (0.5)		100	Toluene	42
5	$Cu(OAc)_2$ (3.0)	TBPB (0.5)		100	Toluene	38
6	$CuCl_2$(3.0)	BPO (0.5)		100	Toluene	46
7	CuBr (3.0)	BPO (0.5)		100	Toluene	40
8	CuI (3.0)	BPO (0.5)		100	Toluene	38
9	CuCl (3.0)	BPO (0.5)		100	Toluene	42
10	$Cu(OAc)_2.H_2O$ (3.0)	BPO (0.5)		100	Toluene	48
11	Zn $(OAc)_2$ (3.0)	BPO (0.5)		100	Toluene	36
12	$FeCl_3$(3.0)	BPO (0.5)		100	Toluene	12
13	$Cu(OAc)_2$ (3.0)	BPO (0.5)		110	Toluene	55
14	$Cu(OAc)_2$ (3.0)	BPO (0.5)		120	Toluene	53
15	$Cu(OAc)_2$ (3.0)	BPO (0.5)		90	Toluene	51
16	$Cu(OAc)_2$ (3.0)	BPO (0.5)		80	Toluene	44
17	$Cu(OAc)_2$ (2.0)	BPO (0.5)		100	Toluene	56
18	$Cu(OAc)_2$ (1.0)	BPO (0.5)		100	Toluene	48
19	$Cu(OAc)_2$ (3.0)	BPO (0.25)		100	Toluene	55
20	$Cu(OAc)_2$ (3.0)	BPO (0.75)		100	Toluene	53
21	$Cu(OAc)_2$ (3.0)	BPO (0.5)		100	DMSO	50
22	$Cu(OAc)_2$ (3.0)	BPO (0.5)		100	Dioxane	40
23	$Cu(OAc)_2$ (3.0)	BPO (0.5)		100	DEC	42
24	$Cu(OAc)_2$ (3.0)	BPO (0.5)		100	MeCN	40
25	$Cu(OAc)_2$ (3.0)	BPO (0.5)		100	DMF	56
26	$Cu(OAc)_2$ (3.0)	BPO (0.5)	Na_2CO_3 (2.5)	100	Toluene	54
27	$Cu(OAc)_2$ (3.0)	BPO (0.5)	Cs_2CO_3 (2.5)	100	Toluene	41
**28**	**$Cu(OAc)_2$ (3.0)**	**BPO (0.5)**	**$K_2S_2O_8$ (2.5)**	**100**	**Toluene**	**79**
29	$Cu(OAc)_2$ (3.0)	BPO (0.5)	$K_2S_2O_8$ (1.5)	100	Toluene	60
30	$Cu(OAc)_2$ (3.0)	BPO (0.5)	$K_2S_2O_8$ (3.5)	100	Toluene	70

[a] Reaction conditions: 3-methyl-1-phenyl-1*H*-pyrazol-5-amine **1a** (0.2 mmol) in solvent (2.0 mL) at different temperatures for 10 hours under air in closed vial. [b] Isolated yields of **2a** based on **1a**.

BPO= Benzoyl peroxide, TBHP= Tert-butyl hydroperoxide, DDQ= 2,3-dichloro-5,6-dicyano-1,4-benzoquinone, TBPB= Tert-butyl peroxyl benzoate.

Due to the emergence of new configurations, it sparked a great deal of interests and prompted us to subject **3a** to condition optimization. 3-Methyl-1-phenyl-1*H*-pyrazol-5-amine **1a** was still chosen as the substrate model. Under the initial conditions shown in entry 1 of Table S2 for **1a** (0.2 mmol) using $Cu(OAc)_2$ as a catalyst, the desired product **3a** was obtained with a yield of 36%. Different oxidants were then tested and the addition of tert-butyl peroxyl benzo resulted in an increase in yield (Table S2, entries 2–5). On this basis, different Cu catalysts were experimented and it was found that the yield was enhanced when $CuCl_2$ was used as catalyst (Table S2, entries 6–10). Then, the addition of different additives was used to test the experimental effect and it was found that the addition of Na_2CO_3 promotes the reaction and results in an increase in yield (Table S2, entries 11–15). Different solvent also screened, such as DMF, DMSO, 1,4-dioxane, diethyl carbonate, acetonitrile, and dimethylacetamide, but all resulted in lower yields (Table S2, entries 16–22). Subsequently, by adding different ligands, such as 2,2'-bipyridine and 1,10-phenantnroline, it was found that the addition of 1,10-Phenantnroline leads to a higher increase in yield (Table S2, entries 23–24). Based on this, we changed the temperature to observe the experimental effect and found that the optimal reaction temperature was still 130 °C (Table S2, entries 25–27). Finally, the yield decreased when the equivalent of $CuCl_2$ was increased to 40% or decreased to 10% (Table S2, entries 28–29).

Table S2. Optimization studies for the preparation of **3a** [a, b]

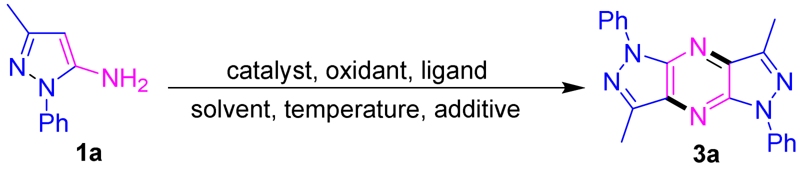

Entry	Catalysts (equiv.)	Oxidants (equiv.)	Additives (equiv.)	Ligands (equiv.)	Temp (°C)	Solvent	Yield (%)
1	$Cu(OAc)_2$ (20%)				130	Toluene	36
2	$Cu(OAc)_2$ (20%)	BPO (0.5)			130	Toluene	38
3	$Cu(OAc)_2$ (20%)	TBHP (0.5)			130	Toluene	37
4	$Cu(OAc)_2$ (20%)	DDQ (0.5)			130	Toluene	32
5	$Cu(OAc)_2$ (20%)	TBPB (0.5)			130	Toluene	41
6	$CuCl_2$ (20%)	TBPB (0.5)			130	Toluene	47
7	CuBr (20%)	TBPB (0.5)			130	Toluene	40
8	CuI (20%)	TBPB (0.5)			130	Toluene	38
9	CuCl (20%)	TBPB (0.5)			130	Toluene	41
10	$Cu(OAc)_2.H_2O$(20%)	TBPB (0.5)			130	Toluene	43
11	$CuCl_2$ (20%)	TBPB (0.5)	Na_2CO_3 (2.5)		130	Toluene	51
12	$CuCl_2$ (20%)	TBPB (0.5)	Cs_2CO_3 (2.5)		130	Toluene	34
13	$CuCl_2$ (20%)	TBPB (0.5)	tBuONa (2.5)		130	Toluene	36
14	$CuCl_2$ (20%)	TBPB (0.5)	KI (2.5)		130	Toluene	29
15	$CuCl_2$ (20%)	TBPB (0.5)	$K_2S_2O_8$ (2.5)		130	Toluene	38

16	$CuCl_2$ (20%)	TBPB (0.5)	Na_2CO_3 (2.5)		130	Toluene	48
17	$CuCl_2$ (20%)	TBPB (0.5)	Na_2CO_3 (2.5)		130	DMF	35
18	$CuCl_2$ (20%)	TBPB (0.5)	Na_2CO_3 (2.5)		130	DMSO	32
19	$CuCl_2$ (20%)	TBPB (0.5)	Na_2CO_3 (2.5)		130	NMP	24
20	$CuCl_2$ (20%)	TBPB (0.5)	Na_2CO_3 (2.5)		130	Dioxane	26
21	$CuCl_2$ (20%)	TBPB (0.5)	Na_2CO_3 (2.5)		130	DEC	24
22	$CuCl_2$ (20%)	TBPB (0.5)	Na_2CO_3 (2.5)		130	DMAC	21
23	$CuCl_2$ (20%)	TBPB (0.5)	Na_2CO_3 (2.5)	Bipy (0.3)	130	Toluene	49
**24**	**$CuCl_2$ (20%)**	**TBPB (0.5)**	**Na_2CO_3 (2.5)**	**1,10-Phen (0.3)**	**130**	**Toluene**	**59**
25	$CuCl_2$ (20%)	TBPB (0.5)	Na_2CO_3 (2.5)	1,10-Phen (0.3)	120	Toluene	46
26	$CuCl_2$ (20%)	TBPB (0.5)	Na_2CO_3 (2.5)	1,10-Phen (0.3)	100	Toluene	38
27	$CuCl_2$ (20%)	TBPB (0.5)	Na_2CO_3 (2.5)	1,10-Phen (0.3)	80	Toluene	29
28	$CuCl_2$ (40%)	TBPB (0.5)	Na_2CO_3 (2.5)	1,10-Phen (0.3)	130	Toluene	50
29	$CuCl_2$ (10%)	TBPB (0.5)	Na_2CO_3 (2.5)	1,10-Phen (0.3)	130	Toluene	35

[a)] Reaction conditions: 3-methyl-1-phenyl-1*H*-pyrazol-5-amine **1a** (0.2 mmol), in solvent (2.0 mL) at different temperatures for 12 hours under air in closed vial. [b)] Isolated yields. BPO = benzoyl peroxide, TBHP = tert-butyl hydroperoxide, DDQ = 2,3-dichloro-5,6-dicyano-1,4-benzoquinone, TBPB = tert-butyl peroxy benzoate, Bipy = 2,2-bipyridine, 1,10-phen = 1,10-phenanthroline.

3. X-Ray Crystal Structure and Crystallographic Data

The purified compound **2a** is dissolved in dichloromethane and petroleum ether, and placed in a dark cabinet to slowly evaporate. After several days, a yellow bulk crystal is obtained. The X-ray crystal-structure determinations were obtained on a Bruker Smart APEX CCD area detector diffractometer at 296(2) K.

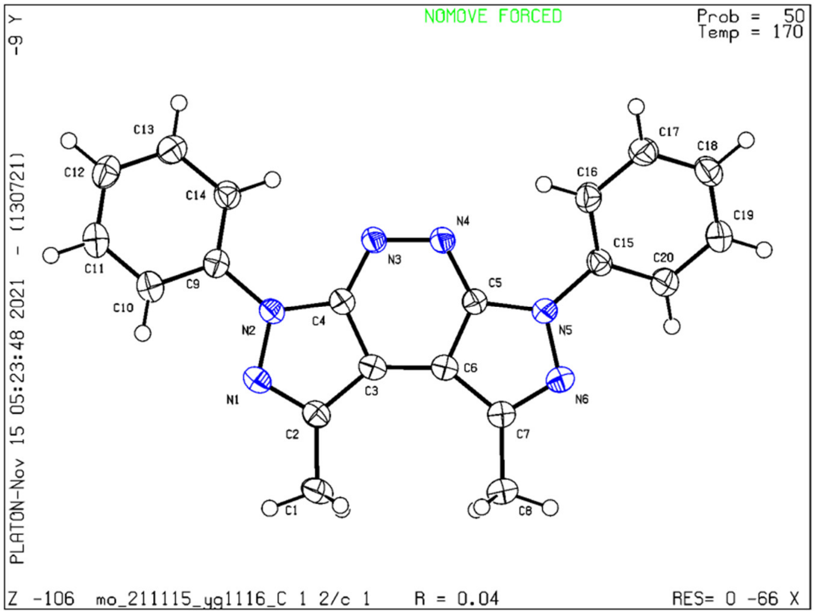

Figure S1. X-ray crystal structure of compound **2a**

Table S3. Crystal data and structure refinement for compound 2a.

Identification code	CCDC: 2322498
Empirical formula	$C_{20}H_{16}N_6$
Formula weight	340.39
Temperature/K	170.0
Crystal system	monoclinic
Space group	C2/c
a/Å	19.2428(11)
b/Å	7.5679(5)
c/Å	23.9148(17)
α/°	90
β/°	108.533(2)
γ/°	90
Volume/Å^3	3302.0(4)
Z	8
ρ_{calc}g/cm^3	1.369
μ/mm^{-1}	0.087
F (000)	1424.0
Crystal size/mm^3	0.48 × 0.136× 0.1
Radiation	MoKα (λ = 0.71073)
2Θ range for data collection/	4.76 to 54.226
Index ranges	$-24 \le h \le 22$, $-9 \le k \le 9$, $-30 \le 1 \le 30$
Reflections collected	18060
Independent reflections	3649 [R_{int} = 0.0701, R_{sigma} = 0.0506]
Data/restraints/parameters	3649/0/237
Goodness-of-fit on F^2	1.024
Final R indexes [I>=2σ (I)]	R_1= 0.0444, wR_2= 0.1106
Final R indexes [all data]	R_1= 0.0589, wR_2= 0.1224
Largest diff. peak/hole / e Å^{-3}	0.20/-0.32

The purified compound **3a** is dissolved in dichloromethane, methanol and petroleum ether, and placed in a dark cabinet to slowly evaporate. After several days, a yellow bulk crystal is obtained. The X-ray crystal-structure determinations were obtained on a Bruker Smart APEX CCD area detector diffractometer at 296(2) K.

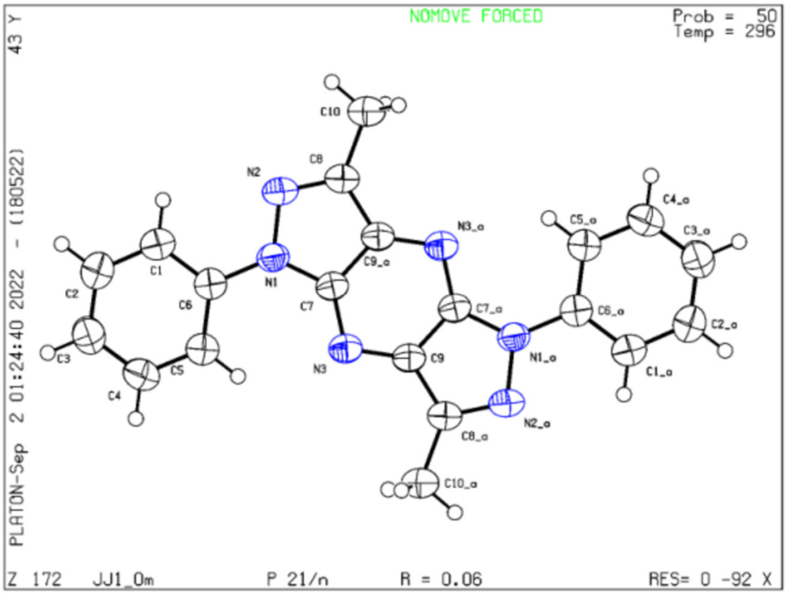

Figure S2. X-ray crystal structure of compound **3a**

Table S4. Crystal data and structure refinement for compound 3a.

Identification code	CCDC: 2322499
Empirical formula	$C_{20}H_{16}N_6$
Formula weight	340.39
Temperature/K	296 (2)
Crystal system	monoclinic
Space group	P21/n
a/Å	4.947(2)
b/Å	14.404(7)
c/Å	11.794(6)
α/°	90
β/°	94.857(8)
γ/°	90
Volume/Å^3	837.3(7)
Z	2
ρ_{calc}g/cm^3	1.350
μ/mm^{-1}	0.085
F (000)	356.0
Crystal size/mm^3	0.18 × 0.16 × 0.07
Radiation	MoKα (λ = 0.71073)
2Θ range for data collection/	4.474 to 55.164
Index ranges	$-6 \leq h \leq 6$, $-18 \leq k \leq 18$, $-11 \leq l \leq 15$
Reflections collected	5052
Independent reflections	1914 [R_{int} = 0.0506, R_{sigma} = 0.0711]

Data/restraints/parameters	1914/0/119
Goodness-of-fit on F^2	0.972
Final R indexes [I>=2σ (I)]	R_1= 0.0608, wR_2= 0.1421
Final R indexes [all data]	R_1= 0.1142, wR_2= 0.1698
Largest diff. peak/hole / e Å^{-3}	0.17/-0.21

The purified compound **3q** is dissolved in dichloromethane, methanol and petroleum ether, and placed in a dark cabinet to slowly evaporate. After several days, a yellow bulk crystal is obtained. The X-ray crystal-structure determinations were obtained on a Bruker Smart APEX CCD area detector diffractometer at 296(2) K.

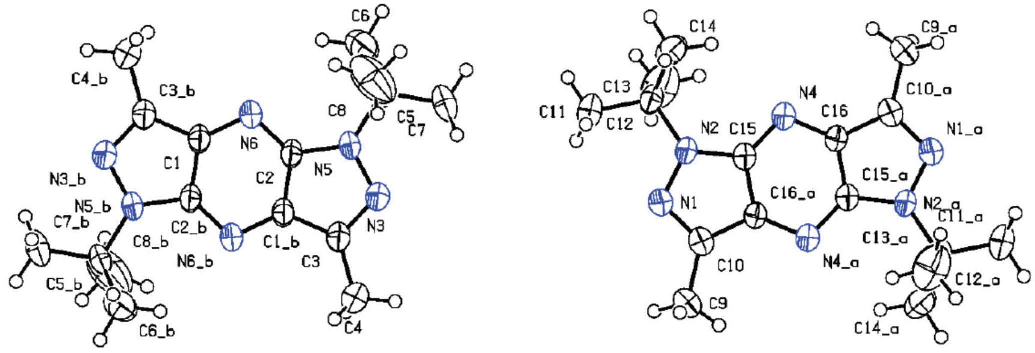

Figure S3. X-ray crystal structure of compound **3q**

Table S5. Crystal data and structure refinement for compound 3q.

Identification code	CCDC: 2322519
Empirical formula	$C_{16}H_{24}N_6$
Formula weight	300.41
Temperature/K	296.15
Crystal system	triclinic
Space group	P-1
a/Å	9.547(13)
b/Å	9.749(13)
c/Å	10.532(14)
α/°	110.097(16)
β/°	97.741(14)
γ/°	107.749(16)
Volume/Å^3	845(2)
Z	2
ρ_{calc}g/cm^3	1.181
μ/mm^{-1}	0.075
F (000)	324.0
Crystal size/mm^3	0.19 × 0.18 × 0.16

Radiation	MoKα (λ = 0.71073)
2Θ range for data collection/	4.274 to 55.024
Index ranges	-12 ≤ h ≤ 9, -12 ≤ k ≤ 12, -11 ≤ l ≤ 13
Reflections collected	4695
Independent reflections	3691 [R_{int} = 0.0386, R_{sigma} = 0.0978]
Data/restraints/parameters	3691/0/207
Goodness-of-fit on F^2	0.933
Final R indexes [I>=2σ (I)]	R_1= 0.0771, wR_2= 0.1908
Final R indexes [all data]	R_1= 0.1641, wR_2= 0.2340
Largest diff. peak/hole / e $Å^{-3}$	0.25/-0.26

4. Fluorescence spectra of compounds 2 and 3

Most of the compounds showed fluorescent nature during UV monitoring. Figure S4 shows the fluorescence phenomena of compounds **2** and **3** at a concentration of $1x10^{-5}$ M in CH_2Cl_2 under daylight, 254 nm UV, and 365 nm UV, respectively. Based on our observations and literature reports,[4] we proceeded to characterize their fluorescence performance.

All the compounds were weighed on an electronic balance 5 mg into a 30 mL syringe vial, the volume of CH_2Cl_2 required was calculated according to the following formula, the CH_2Cl_2 was added sequentially to dissolve the samples to obtain a mother solution of $1x10^{-3}$ M firstly, then 40 uL of the solution was aspirated into a 5 mL syringe vial using a 100 uL pipette respectively, then 4 mL of CH_2Cl_2 was added sequentially to dilute the mother solution by 100-fold to give a solution of $1x10^{-5}$ M for testing.

The formula for calculating the CH_2Cl_2 volume is as follows:

$v= m / M \times c$

v represents the volume of CH_2Cl_2 required for different samples, m= 5 mg, M represents the relative molecular mass of the different compounds, $c=1x10^{-3}$ M.

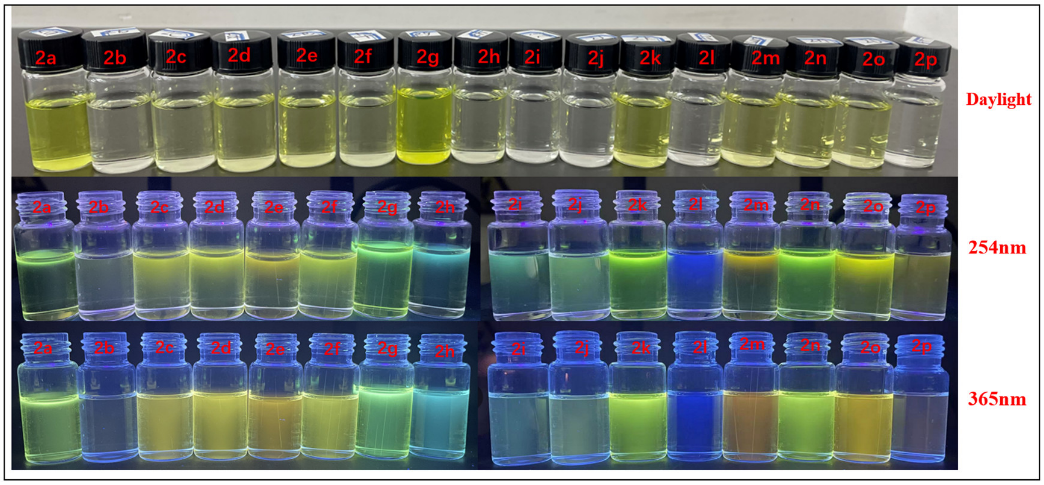

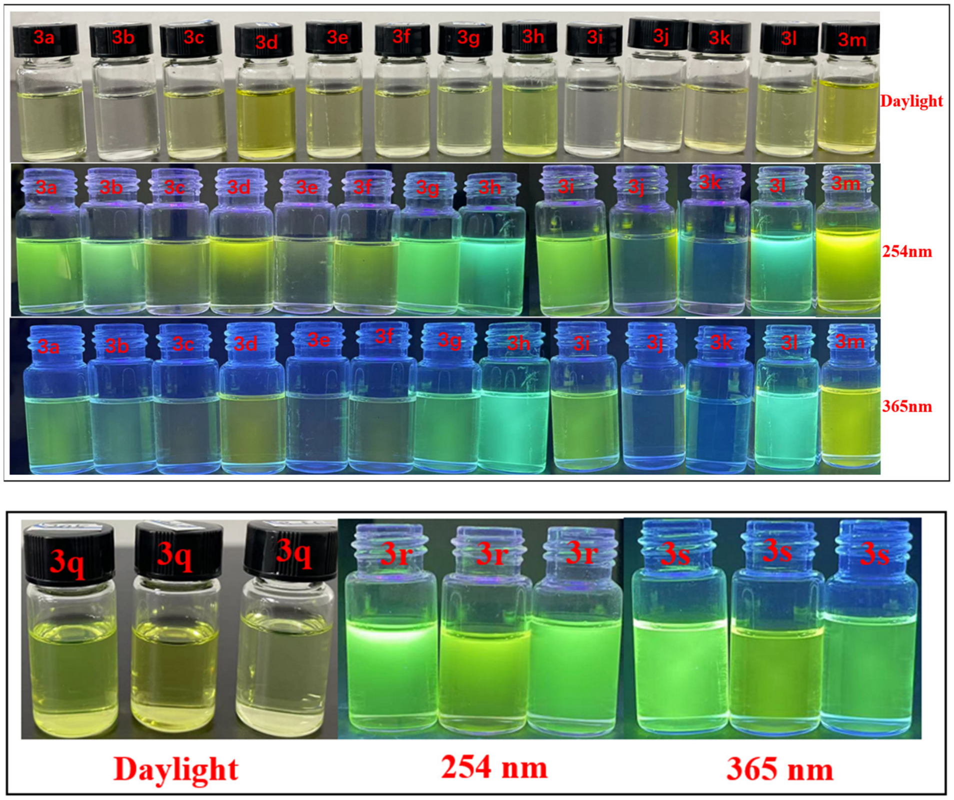

Figure S4. Fluorescence of compounds **2** and **3** at the concentration of 1x10^{-5} M in CH_2Cl_2.

The UV absorption wavelengths of compound **2** ranged from 238 to 309 nm, the maximum fluorescence emission wavelengths of these compounds were from 294 to 511 nm, and the stokes shifts of these compounds ranged from 20 to 238. Among the compounds **2**, compound **2c** have the highest fluorescence quantum yield of 21.8%.

Table S6. Photophysical properties of compound **2**

Compd.	$\lambda_{max}{}^{a}$(nm)	$\lambda_{em}{}^{b}$(nm)	Stokes shift (nm)	$\Phi_F{}^{c}$ (%)
**2a**	279	502	223	3.0
**2b**	266	298	32	5.2
**2c**	272	295	23	21.8
**2d**	273	300	27	10.3
**2e**	238	304	66	2.2
**2f**	274	296	22	9.0
**2g**	309	492	183	7.4
**2h**	312	478	166	7.6
**2i**	271	294	23	11.6
**2j**	273	300	27	9.1
**2k**	273	293	20	2.0
**2l**	271	294	23	12.6
**2m**	273	511	238	8.0
**2n**	272	296	24	7.5
**2p**	274	298	24	2.0

Concentration: 1 x10^{-5}M in CH_2Cl_2. [a]Absorption maximum. [b]Emission maximum.[c] Quinine sulfate was used as a standard for fluorescence QY = 0.54 (0.5 M H_2SO_4).

The UV absorption wavelengths of compound **3** ranged from 284 to 304 nm, and the maximum fluorescence emission wavelengths of these compounds were from 302 to 530 nm, and the stokes shifts of most compound over 200. Among the compounds **3**, compound **3q** had the highest fluorescence quantum yield of 16.2%.

Table S7. Photophysical properties of compound **3**

Compd.	$\lambda_{max}{}^a$(nm)	$\lambda_{em}{}^b$(nm)	Stokes shift (nm)	$\Phi_F{}^c$ (%)
**3a**	284	505	221	14.0
**3b**	304	501	197	8.6
**3c**	284	523	239	5.3
**3d**	286	530	244	2.4
**3e**	285	530	245	1.7
**3f**	285	522	237	2.7
**3g**	285	495	210	13.8
**3h**	284	496	212	4.3
**3i**	287	474	187	9.2
**3j**	274	302	28	6.2
**3k**	287	544	257	0.3
**3l**	284	503	219	12.3
**3m**	289	534	245	8.0
**3q**	284	499	215	14.3
**3r**	283	500	217	16.2
**3s**	285	512	227	5.9

Concentration: 1x10^{-5} M in CH_2Cl_2. [a]Absorption maximum. [b]Emission maximum.[c] Quinine sulfate was used as a standard for fluorescence QY = 0.54 (0.5 M H_2SO_4).

The calculation formula of quantum yield was adopted as follows:

$\Phi_s = F_s/F_r \times A_r/A_s \times ns^2/ nr^2 \times \Phi_r$

Where, s and r represent sample and reference respectively. A is the absorbance. F is the relative integrated fluorescence intensity, and n is the refractive index of the solvent.

We prepared **2a-2p**, **3a-3m**, and **3q-3s** to a concentration of 1x10^{-5}M in CH_2Cl_2 and tested the UV absorbance at room temperature with a Shimadzu UV 2000. Next, we tested the fluorescence emission data when excited at its maximum absorption wavelength with a HITACHI F7000, and calculated the fluorescence quantum yield using the above equation.

Finally, we tested the effect of solvent polarity on the fluorescence spectrum, **2a** and **3l** were chosen as examples, the results showed that both compounds **2** and **3** were observed to be significantly redshifted and blue shifted in methanol and toluene, respectively, with nearly similar emission maxima in dichloromethane, acetonitrile, and dimethyl sulfoxide.

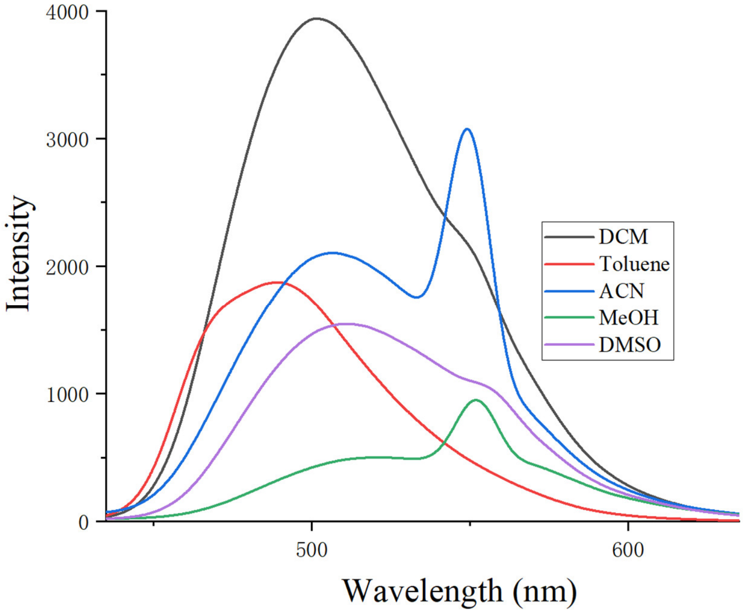

Figure S5. The Fluorescence Emission Spectra of **2a** in CH_2Cl_2, Toluene, ACN, CH_3OH and DMSO at a concentration of $1x10^{-5}$ mol/L.

We chose compound **2a** as the sample to screen several solvents, such as CH_2Cl_2, toluene, ACN, CH_3OH and DMSO at the concentration of $1x10^{-5}$ mol/L. The data of the fluorescence emission was tested by HITACHI F7000 at room temperature.

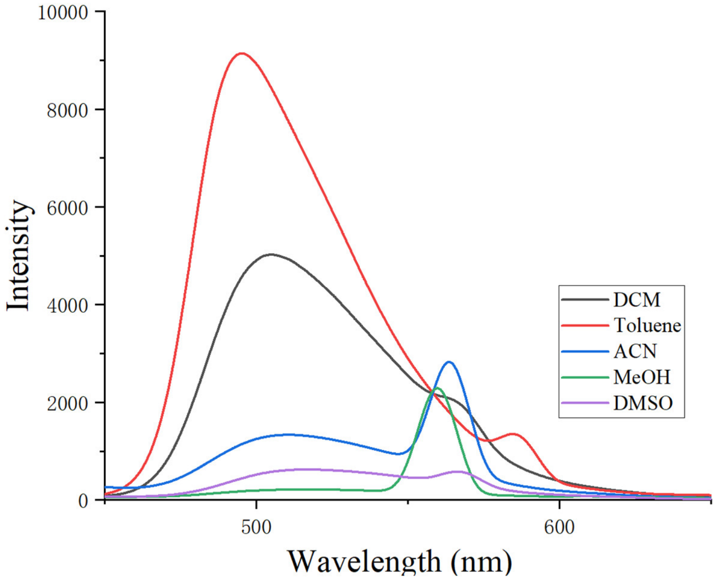

Figure S6. The Fluorescence Emission Spectra of **3l** in CH_2Cl_2, Toluene, ACN, CH_3OH and DMSO at a concentration of $1x10^{-5}$ mol/L.

Then we chose compound **3l** as the sample in the above solvents at a concentration of 1x10^{-5} mol/L. The data of the fluorescence emission was tested by HITACHI F7000 at room temperature.

5. Experimental Procedures

5.1 General Procedure for the Synthesis of 2 (2a as an example).

3-Methyl-1-phenyl-1*H*-pyrazol-5-amine (**1a**) (35 mg, 0.2 mmol, 1.0 equiv.), $Cu(OAc)_2$ (55 mg, 0.3 mmol, 3.0 equiv.), benzoyl peroxide (BPO) (12 mg, 0.05 mmol, 0.5 equiv.), $K_2S_2O_8$ (68 mg, 0.25 mmol, 2.5 equiv.) were added to a screw-cap test tube, and then solvent of toluene (2.0 mL) was added. The mixture was then stirred at 100 °C in oil bath for 10 h under air atmosphere. After the reaction was completed (monitored by TLC), and 50 mL of water was added to the mixture, which was then extracted three times with CH_2Cl_2 (3× 50 mL). The combined organic phase was dried with anhydrous Na_2SO_4, filtered and concentrated under reduced pressure. The crude residues were purified by column chromatography (silica gel: 200−300 mesh, solvent system: petroleum ether/ethyl acetate = 5:1) to obtain the corresponding products **2a** (27 mg, 79%).

Scale-up experiment: A 100 mL pressure vial was charged with 3-methyl-1-phenyl-1*H*-pyrazol-5-amine (**1a**) (346 mg, 2.0 mmol, 1.0 equiv.), $Cu(OAc)_2$ (546 mg, 3.0 mmol, 3.0 equiv.), benzoyl peroxide (BPO) (245 mg, 0.5 mmol, 0.5 equiv.), $K_2S_2O_8$ (675mg, 2.5 mmol, 2.5 equiv.) in toluene (8.0 mL) were added. The reaction was stirred at 100 °C in oil bath for 10 h under air atmosphere. After the reaction was completed (monitored by TLC). Then added 150 mL water to the mixture, then extracted with CH_2Cl_2 three times (3×150 mL). The combined organic phase was dried with anhydrous Na_2SO_4, filtered and concentrated under reduced pressure. The residue was purified by flash column chromatography (silica gel: 200−300 mesh, solvent system: petroleum ether/ethyl acetate = 5:1) to yield the corresponding product **2a** (204 mg, 60%).

5.2 General Procedure for the Synthesis of 3 (3a as an example).

3-Methyl-1-phenyl-1*H*-pyrazol-5-amine (**1a**) (35 mg, 0.2 mmol, 1.0 equiv.), $CuCl_2$ (3.0 mg, 0.02 mmol, 20 mol%), 1,10-phenanthroline (5.4 mg, 0.03 mmol, 0.3 equiv.), tert-butyl peroxybenzoate (10 uL, 0.05 mmol, 0.5 equiv.), Na_2CO_3 (27 mg, 0.25 mmol, 2.5 equiv.) were added to a screw-cap test tube, and then solvent of toluene (2.0 mL) was added. The mixture was then stirred at 130 °C in oil bath for 12 h under air atmosphere. After the reaction was completed (monitored by TLC), and 50 mL of water was added to the mixture, which was then extracted three times with CH_2Cl_2 (3×50 mL). The combined organic phase was dried with anhydrous Na_2SO_4, filtered and concentrated under reduced pressure. The crude residues were purified by column chromatography (silica gel: 200−300 mesh, solvent system: petroleum ether/ethyl acetate = 10:1) to obtain the corresponding products **3a** (20mg, 59%).

Scale-up experiment: A 100 mL pressure vial was charged with 3-methyl-1-phenyl-1*H*-pyrazol-5-amine (**1a**) (346 mg, 2.0 mmol, 1.0 equiv.), $CuCl_2$ (27 mg, 0.2 mmol, 20 mol%), 1,10-phenanthroline (54 mg, 0.3 mmol, 0.3 equiv.), tert-butyl peroxybenzoate

(88 uL, 0.5 mmol, 0.5 equiv.), Na_2CO_3 (265 mg, 2.5 mmol, 2.5 equiv.) in toluene (8.0 mL) were added. The reaction was stirred at 130 °C in oil bath for 12 h under air atmosphere. After the reaction was completed (monitored by TLC). Then added 150 mL water to the mixture, then extracted with CH_2Cl_2 three times (3×150 mL). The combined organic phase was dried with anhydrous Na_2SO_4, filtered and concentrated under reduced pressure. The residue was purified by flash column chromatography (silica gel: 200−300 mesh, solvent system: petroleum ether/ethyl acetate = 10:1) to yield the corresponding product **2a** (163 mg, 48%).

5.3 Procedure for the Synthesis of (1-Cyclopropylvinyl) benzene (5) (Adapted from Literature)

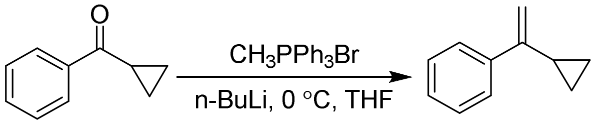

5

Scheme S1. Synthesis of (1-cyclopropylvinyl) benzene **(5)**

In a 50 mL round bottom flask methyltriphenylphosphonium bromide (4.64 g, 13 mmol, 1.3 equiv.) was dissolved in anhydrous THF (30 mL) and the mixture was cooled to 0 °C. nBuLi (4.0 mL, 2.5 M, 10 mmol) was added dropwise. After 30 minutes of reaction, to this mixture cyclopropyl phenyl ketone (1.46 g, 10.0 mmol, 1.0 equiv.) was added dropwise and the reaction was left to stir for 4 hours, at which the reaction was deemed done by TLC analysis. After the reaction is completed, add NH_4Cl solution to the reaction solution. And then the mixture was extracted with EtOAc, the combined organic phases were dried over anhydrous Na_2SO_4 and the solvent was evaporated under vacuum. The resulting crude product was purified by flash column chromatography on silica gel using ethyl acetate as the eluent to give the pure product **5**.[1]

5.4 Procedure for the Synthesis of (*E*)-1,2-bis(3-methyl-1-phenyl-1*H*-pyrazol-5-yl) diazene (4a) (Adapted from Literature)

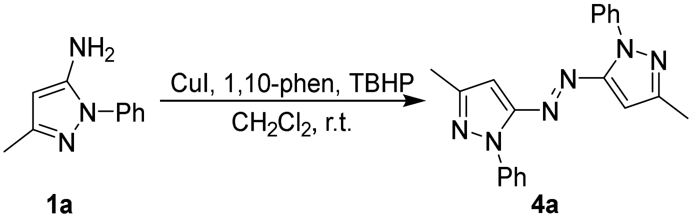

1a **4a**

Scheme S2. Synthesis of (*E*)-1,2-bis(3-methyl-1-phenyl-1*H*-pyrazol-5-yl) diazene **(4a)**

3-Methyl-1-phenyl-1*H*-pyrazol-5-amine **(1a)** (173 mg, 1.0 mmol, 1.0 equiv.) was introduced in a 25 mL reaction flask. CuI (10 mg, 0.05 mmol, 0.1 equiv.), 1,10-

phenanthroline (27 mg, 0.15 mmol, 0.3 equiv.), and CH_2Cl_2 (2.0 mL) as well as TBHP (TBHP 70% solution in water) (210 uL, 1.5 mmol, 3.0 equiv.) were then successively added and the mixture stirred at room temperature for 6 h. After the completion of the reaction (monitored by TLC), the solvent was removed under vacuum. The residue was purified by flash column chromatography (silica gel: 200−300 mesh, solvent system: petroleum ether/ethyl acetate = 8:1) to afford the pure product **4a**.[2]

6. Controlled Experiments

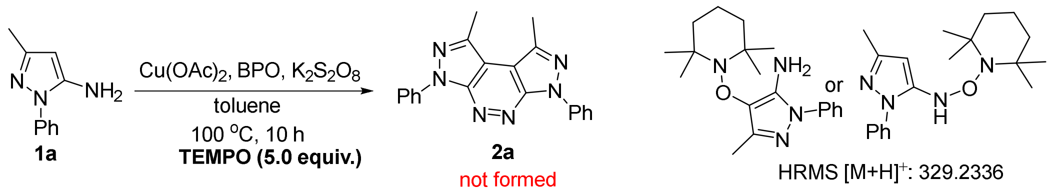

Scheme S3. The reaction with TEMPO as free radical scavenger under scheme 2 conditions.

3-Methyl-1-phenyl-1*H*-pyrazol-5-amine **(1a)** (35 mg, 0.2 mmol, 1.0 equiv.), $Cu(OAc)_2$ (55 mg, 0.3 mmol, 3.0 equiv.), benzoyl peroxide (BPO) (12 mg, 0.05 mmol, 0.5 equiv.), $K_2S_2O_8$ (68 mg, 0.25 mmol, 2.5 equiv.), TEMPO (78 mg, 0.5 mmol, 5 equiv.) were added to a screw-cap test tube, and then solvent of toluene (2.0 mL) was added. The mixture was then stirred at 100 °C in oil bath for 10 h under air atmosphere. After the reaction was completed (monitored by TLC), **2a** was not generated. The crude reaction solution was subjected to HR-MS, the product of **1a** binding to TEMPO could be found.

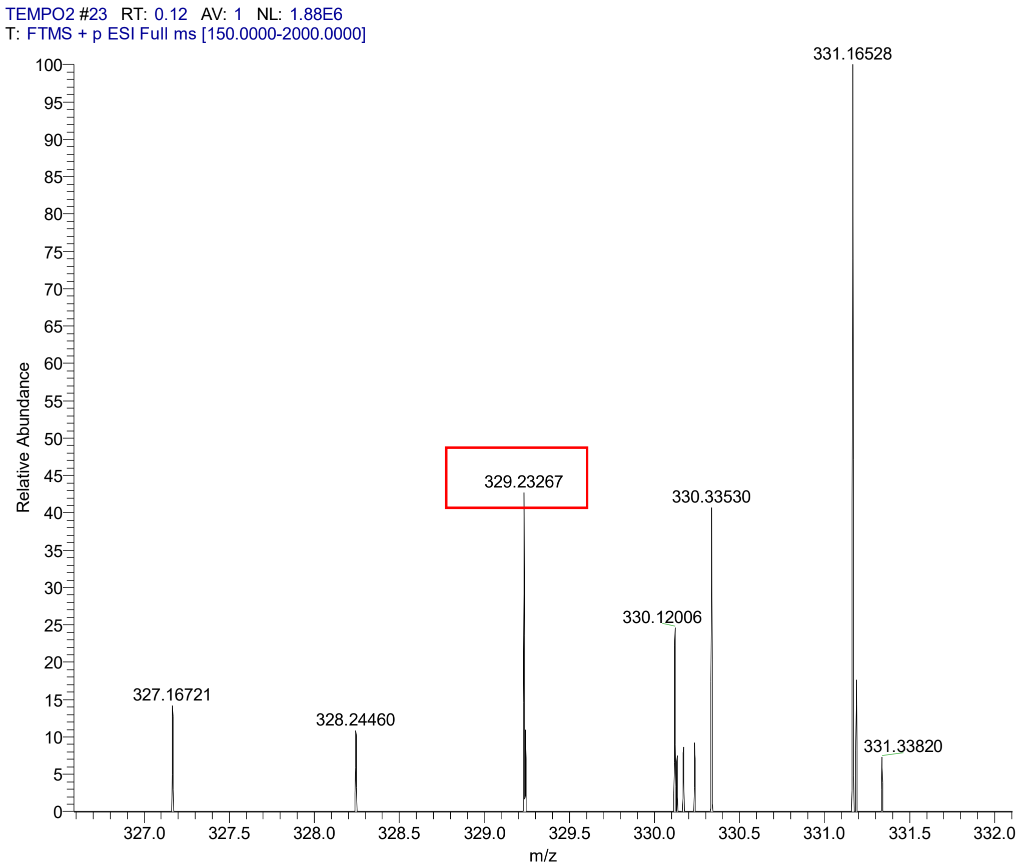

Figure S7. The HRMS spectra for the product of **1a** binding to TEMPO under scheme 2 conditions.

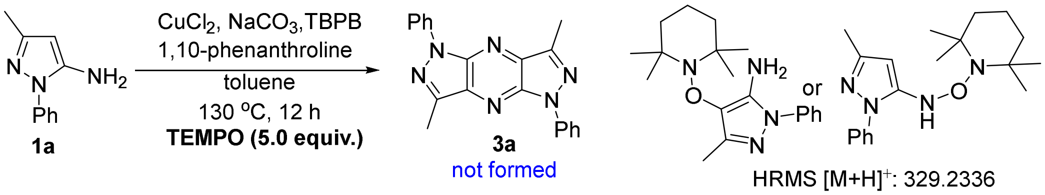

Scheme S4. The reaction with TEMPO as free radical scavenger under scheme 3 conditions

3-Methyl-1-phenyl-1*H*-pyrazol-5-amine (**1a**) (35 mg, 0.2 mmol, 1.0 equiv.), $CuCl_2$(3.0 mg, 0.02 mmol, 20 mol%), 1,10-phenanthroline (5.4 mg, 0.03 mmol, 0.3 equiv.), tert-butyl peroxybenzoate (10 uL, 0.05 mmol, 0.5 equiv.), Na_2CO_3(27 mg, 0.25 mmol, 2.5 equiv.), TEMPO (78 mg, 0.5 mmol, 5.0 equiv.) were added to a screw-cap test tube, and then solvent of toluene (2.0 mL) was added. The mixture was then stirred at 130 °C in oil bath for 12 h under air atmosphere. After the reaction was completed (monitored by TLC), **3a** was not generated. The crude reaction solution was subjected to HR-MS, the product of **1a** binding to TEMPO could be found.

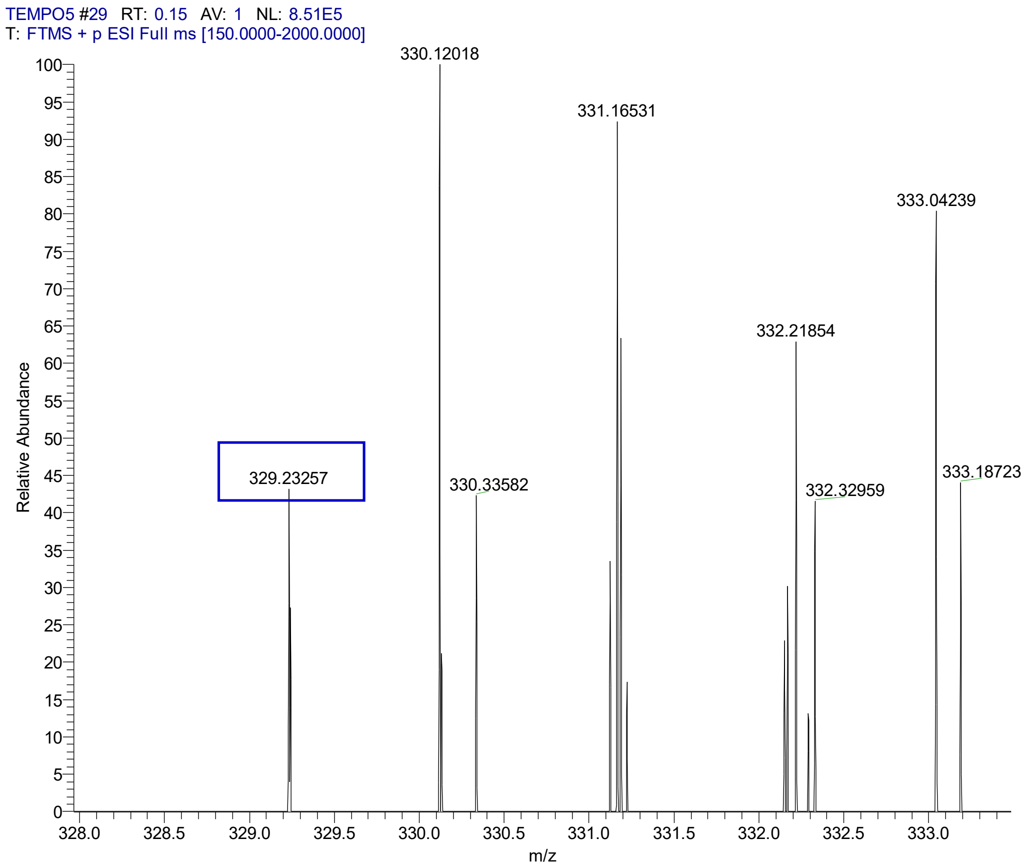

Figure S8. The HRMS spectra for the product of **1a** binding to TEMPO under scheme 3 conditions.

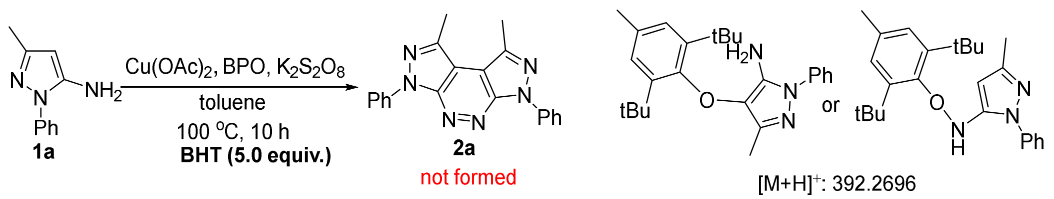

Scheme S5. The reaction with BHT as free radical scavenger under scheme 2 conditions.

3-Methyl-1-phenyl-1*H*-pyrazol-5-amine (**1a**) (35 mg, 0.2 mmol, 1.0 equiv.), $Cu(OAc)_2$ (55 mg, 0.3 mmol, 3.0 equiv.), benzoyl peroxide (BPO) (12 mg, 0.05 mmol, 0.5 equiv.), $K_2S_2O_8$ (68 mg, 0.25 mmol, 2.5 equiv.), BHT (110 mg, 0.5 mmol, 5.0 equiv.) were added to a screw-cap test tube, and then solvent of toluene (2.0 mL) was added. The mixture was then stirred at 100 °C in oil bath for 10 h under air atmosphere. After the reaction was completed (monitored by TLC), **2a** was not generated. The crude reaction solution was subjected to HR-MS, the product of **1a** binding to BHT could be found.

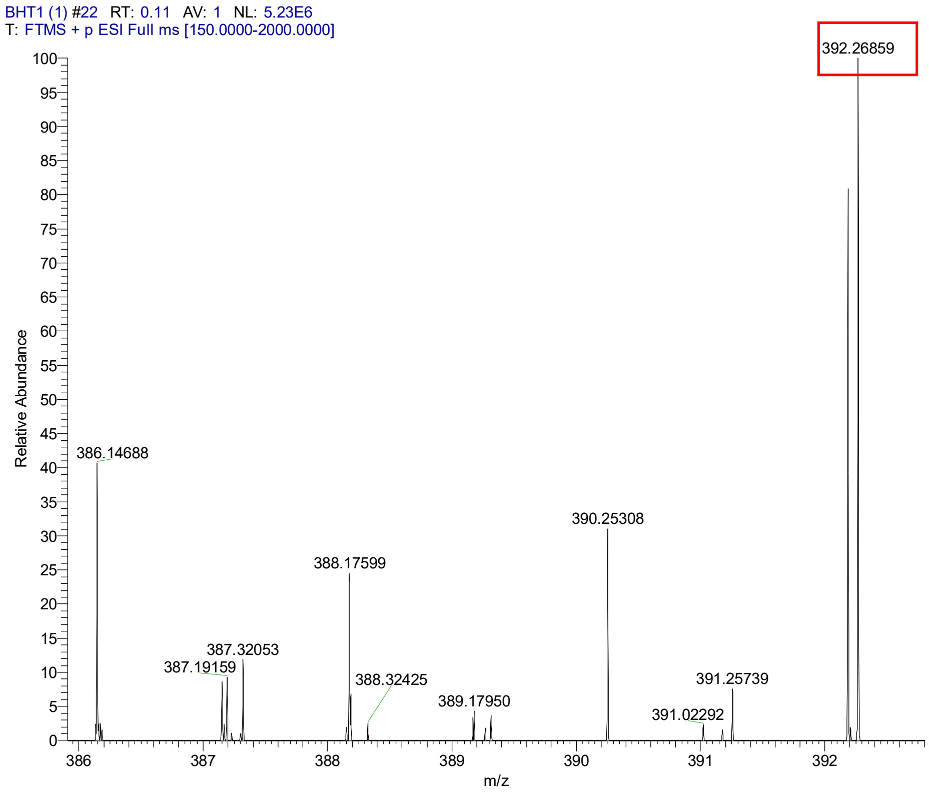

Figure S9. The HRMS spectra for the product of **1a** binding to BHT under scheme 2 conditions.

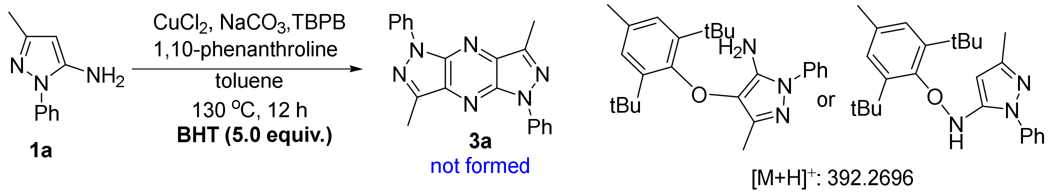

Scheme S6. The reaction with BHT as free radical scavenger under scheme 3 conditions.

3-Methyl-1-phenyl-1*H*-pyrazol-5-amine (**1a**) (35 mg, 0.2 mmol, 1.0 equiv.), $CuCl_2$ (3.0 mg, 0.02 mmol, 20 mol%), 1,10-phenanthroline (5.4 mg, 0.03 mmol, 0.3 equiv.), tert-Butyl peroxybenzoate (10 uL, 0.05 mmol, 0.5 equiv.), Na_2CO_3 (27 mg, 0.25 mmol, 2.5 equiv.), BHT (110 mg, 0.5 mmol, 5 equiv.) were added to a screw-cap test tube, and then solvent of toluene (2.0 mL) was added. The mixture was then stirred at 130 °C in oil bath for 12 h under air atmosphere. After the reaction was completed (monitored by TLC), **3a** was not generated. The crude reaction solution was subjected to HR-MS, the product of **1a** binding to BHT could be found.

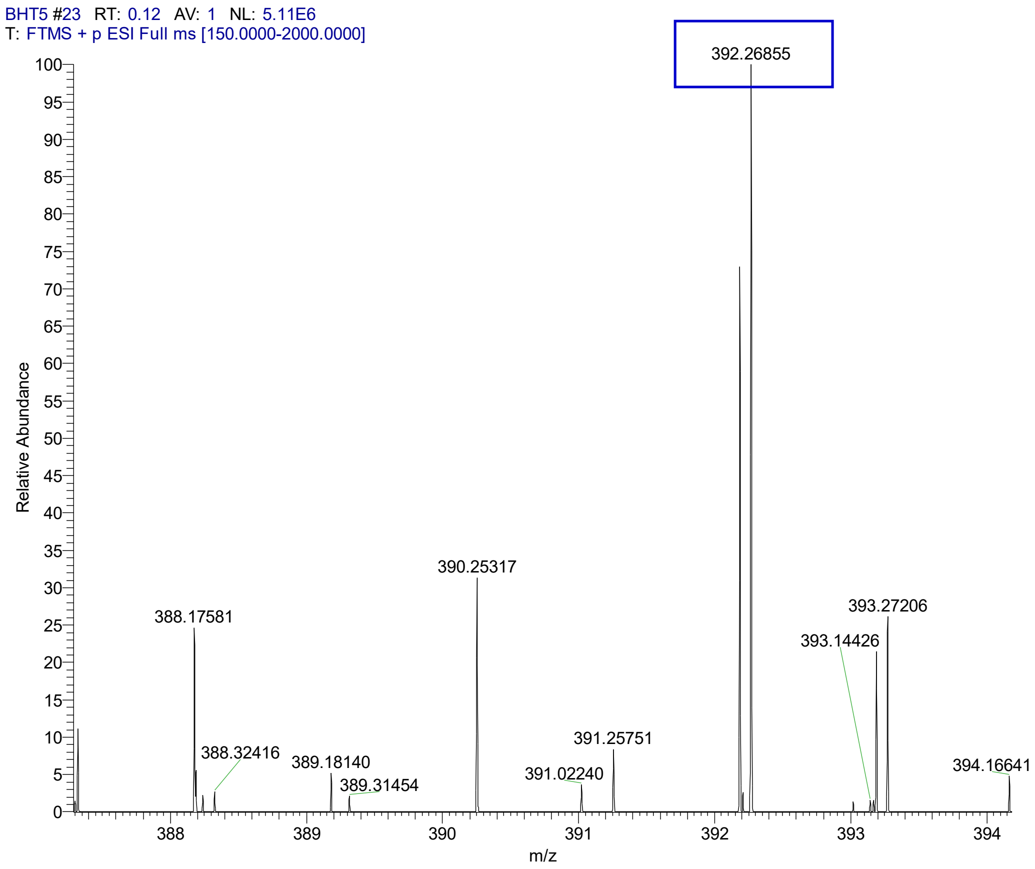

Figure S10. The HRMS spectra for the product of **1a** binding to BHT under scheme 3 conditions.

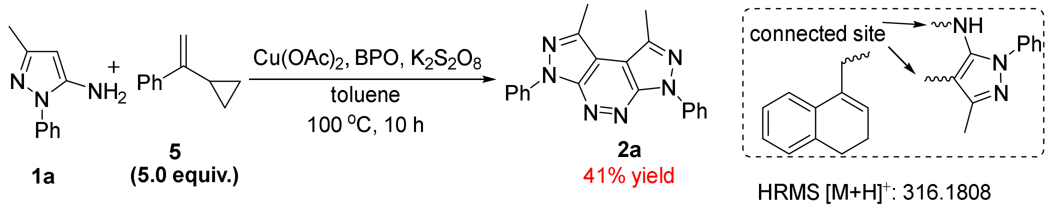

Scheme S7. The reaction of **1a** with **5** under scheme 2 conditions.

3-Methyl-1-phenyl-1*H*-pyrazol-5-amine **(1a)** (35 mg, 0.2 mmol, 1.0 equiv.), (1-cyclopropylvinyl) benzene **(5)** (40 uL, 0.5 mmol, 5.0 equiv.), $Cu(OAc)_2$ (55 mg, 0.3 mmol, 3.0 equiv.), benzoyl peroxide (BPO) (12 mg, 0.05mmol, 0.5 equiv.), $K_2S_2O_8$ (68 mg, 0.25mmol, 2.5 equiv.) were added to a screw-cap test tube, and then solvent of toluene (2.0 mL) was added. The mixture was then stirred at 100 °C in oil bath for 10 h under air atmosphere. After the reaction was completed (monitored by TLC), and 50 mL of water was added to the mixture, which was then extracted three times with CH_2Cl_2 (3×50 mL). The combined organic phase was dried with anhydrous Na_2SO_4, filtered and concentrated under reduced pressure. The crude residues were purified by column chromatography (silica gel: 200−300 mesh, solvent system: petroleum

ether/ethyl acetate = 5:1) to obtain the corresponding products **2a** (14 mg, 41%). The crude reaction solution was subjected to HR-MS, the adduct could be detected.

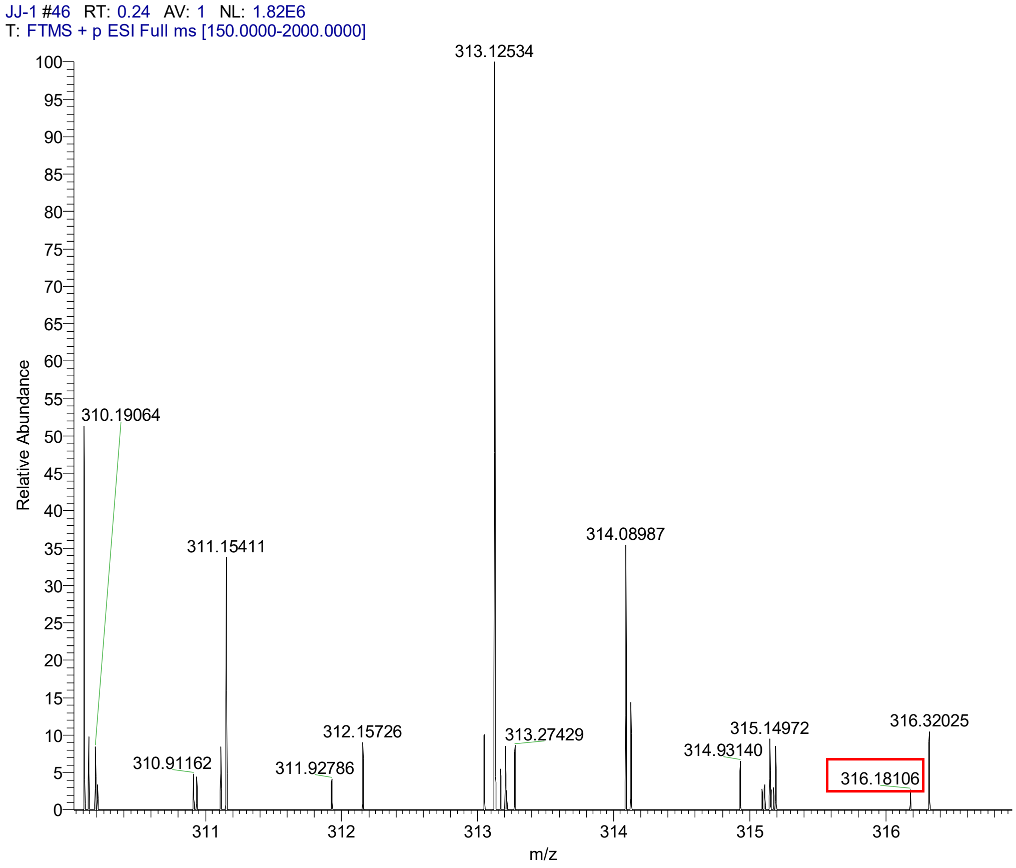

Figure S11. The HRMS spectra for the adduct of **1a** and **5** under scheme 2 conditions.

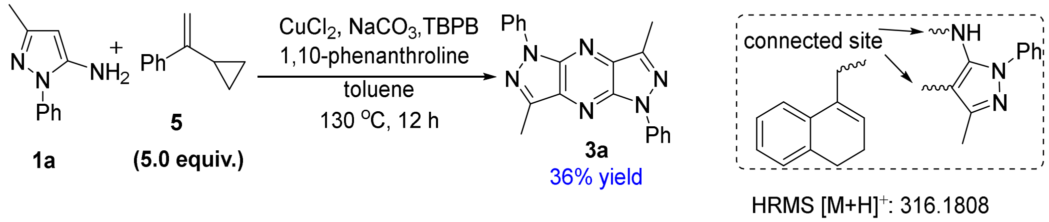

Scheme S8. The reaction of **1a** with **5** under scheme 3 conditions.

3-Methyl-1-phenyl-1*H*-pyrazol-5-amine **(1a)** (35 mg, 0.2 mmol, 1.0 equiv.), (1-cyclopropylvinyl) benzene **(5)** (40 uL, 0.5 mmol, 5.0 equiv.), $CuCl_2$(3.0 mg, 0.02 mmol, 20 mol%), 1,10-phenanthroline (5.4 mg, 0.03 mmol, 0.3 equiv.), tert-butyl peroxybenzoate (10 uL, 0.05 mmol, 0.5 equiv.), Na_2CO_3 (27 mg, 0.25 mmol, 2.5 equiv.) were added to a screw-cap test tube, and then solvent of toluene (2.0 mL) was added. The mixture was then stirred at 130 °C in oil bath for 12 h under air atmosphere. After the reaction was completed (monitored by TLC), and 50 mL of water was added to the mixture, which was then extracted three times with CH_2Cl_2 (3 × 50 mL). The combined

organic phase was dried with anhydrous Na_2SO_4, filtered and concentrated under reduced pressure. The crude residues were purified by column chromatography (silica gel: 200−300 mesh, solvent system: petroleum ether/ethyl acetate = 10:1) to obtain the corresponding products **3a** (12 mg, 36%). The crude reaction solution was subjected to HR-MS, the adduct could be detected.

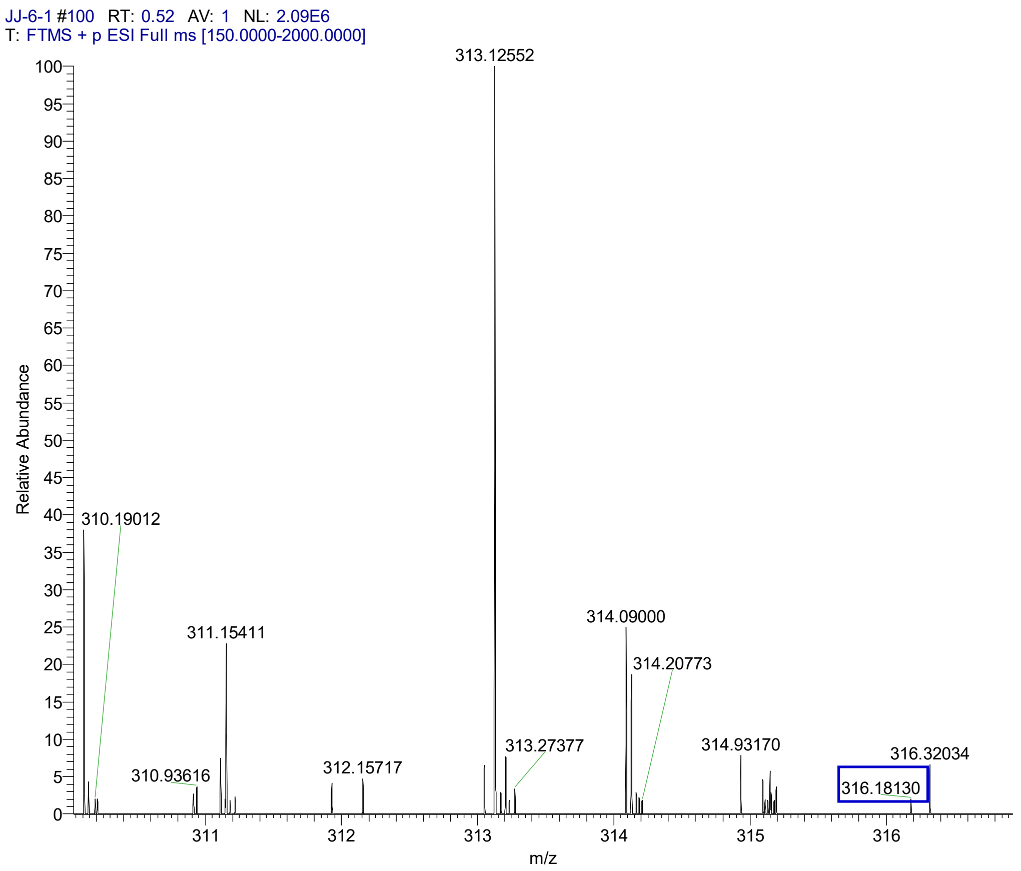

Figure S12. The HRMS spectra for the adduct of **1a** and **5** under scheme 3 conditions.

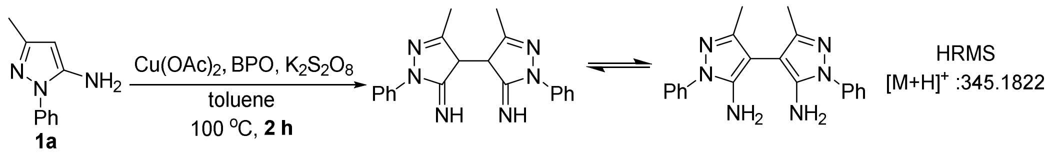

Scheme S9. The reaction with scheme 2 conditions for 2 h.

3-Methyl-1-phenyl-1*H*-pyrazol-5-amine **(1a)** (35 mg, 0.2 mmol, 1.0 equiv.), $Cu(OAc)_2$ (55mg, 0.3 mmol, 3.0 equiv.), benzoyl peroxide (BPO) (12 mg, 0.05 mmol, 0.5 equiv.), $K_2S_2O_8$ (68 mg, 0.25 mmol, 2.5 equiv.) were added to a screw-cap test tube, and then solvent of toluene (2.0 mL) was added. The mixture was then stirred at 100 °C in oil bath for 2 h under air atmosphere. The crude reaction solution was subjected to HR-MS, the intermediates could be found.

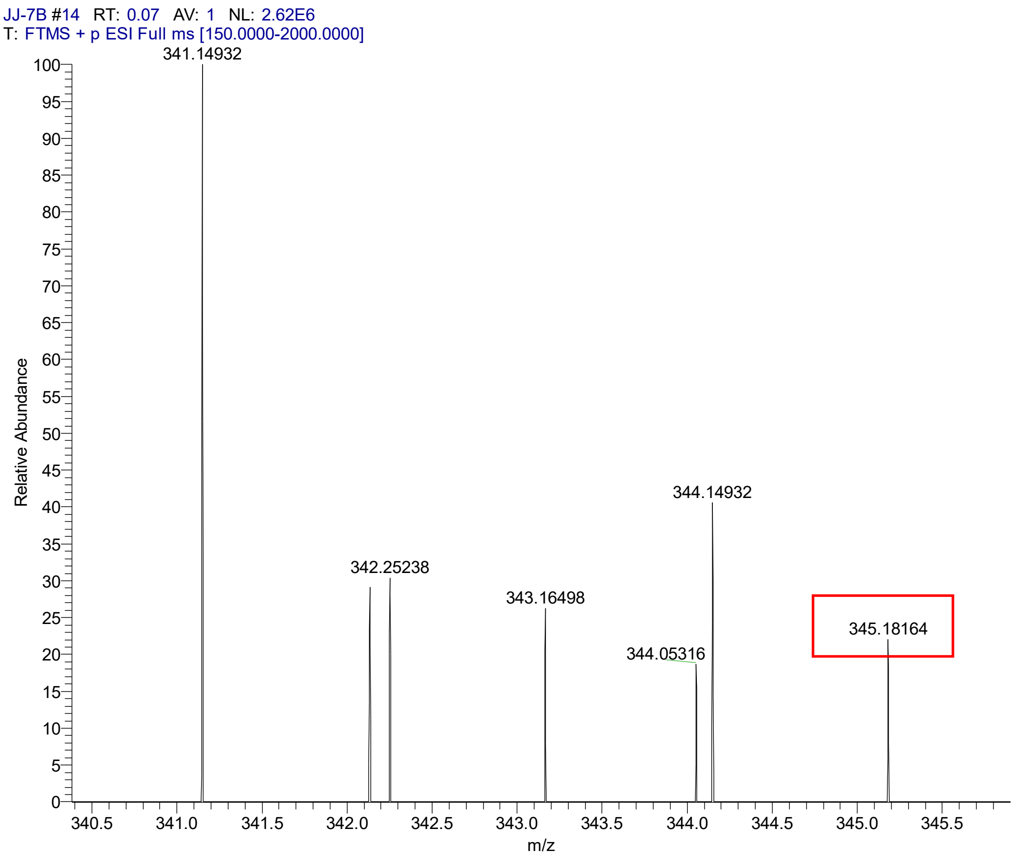

Figure S13. The HRMS spectra for potential intermediates under scheme 2 conditions

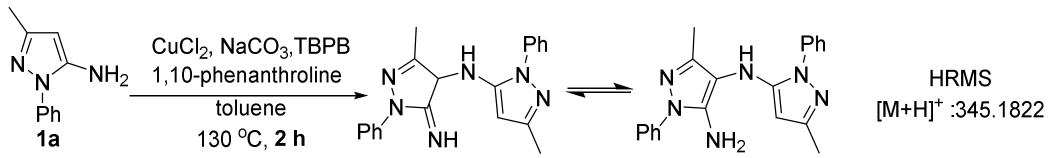

Scheme S10. The reaction with scheme 3 conditions for 2 h.

3-Methyl-1-phenyl-1*H*-pyrazol-5-amine **(1a)** (35 mg, 0.2 mmol, 1.0 equiv.), $CuCl_2$ (3.0 mg, 0.02 mmol, 20 mol%), 1,10-phenanthroline (5.4 mg, 0.03 mmol, 0.3 equiv.), tert-butyl peroxybenzoate (10 uL, 0.05 mmol, 0.5 equiv.), Na_2CO_3 (27 mg, 0.25 mmol, 2.5 equiv.) were added to a screw-cap test tube, and then solvent of toluene (2.0 mL) was added. The mixture was then stirred at 130 °C in oil bath for 2 h under air atmosphere. The crude reaction solution was subjected to HR-MS, the intermediates could be found.

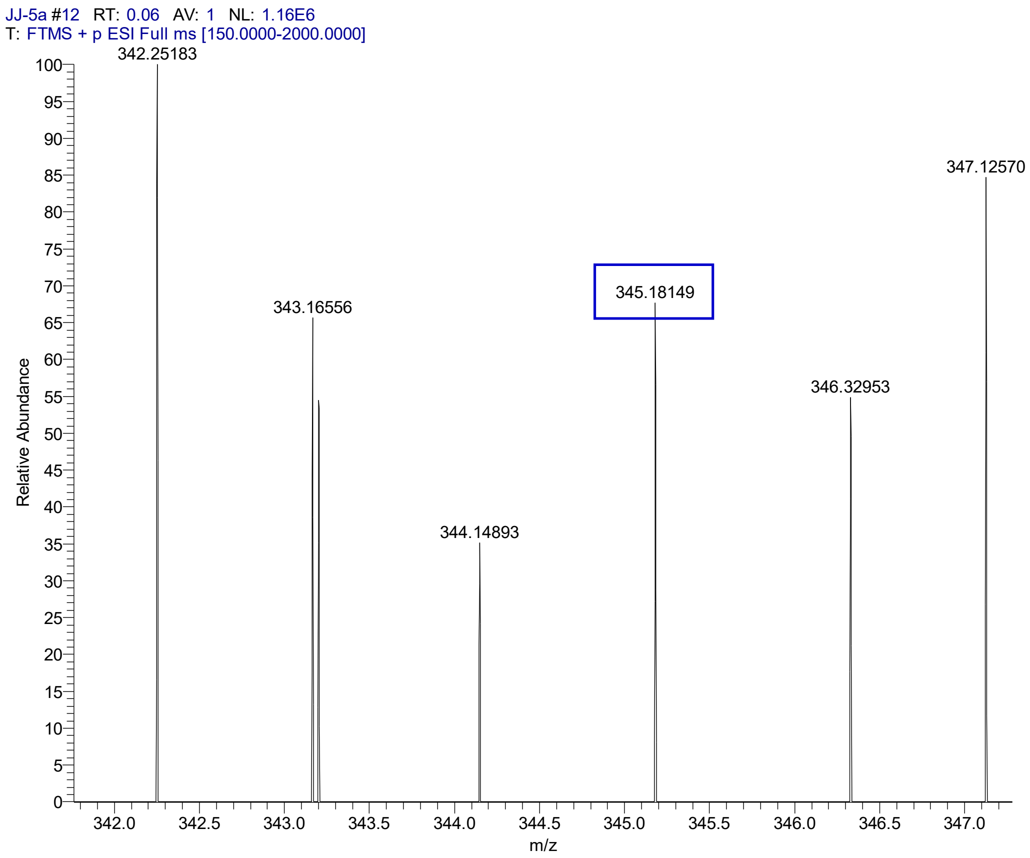

Figure S14. The HRMS spectra for potential intermediates under scheme 3 conditions

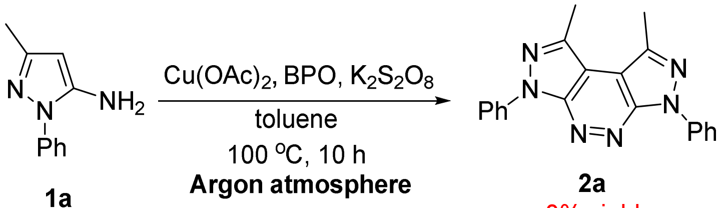

Scheme S11. The reaction with scheme 2 conditions under argon atmosphere

3-Methyl-1-phenyl-1*H*-pyrazol-5-amine (**1a**) (35 mg, 0.2 mmol, 1.0 equiv.), $Cu(OAc)_2$ (55 mg, 0.3 mmol, 3.0 equiv.), benzoyl peroxide (BPO) (12 mg, 0.05mmol, 0.5 equiv.), $K_2S_2O_8$ (68 mg, 0.25 mmol, 2.5 equiv.) were added to a screw-cap test tube, and then solvent of toluene (2.0 mL) was added. The mixture was then stirred at 100 °C in oil bath for 10 h under argon atmosphere. After the reaction was completed (monitored by TLC), and 50 mL of water was added to the mixture, which was then extracted three times with CH_2Cl_2 (3×50 mL). The combined organic phase was dried with anhydrous Na_2SO_4, filtered and concentrated under reduced pressure. The crude residues were purified by column chromatography (silica gel: 200−300 mesh, solvent system: petroleum ether/ethyl acetate = 5:1) to obtain the corresponding products **2a** (2.7 mg, 8%).

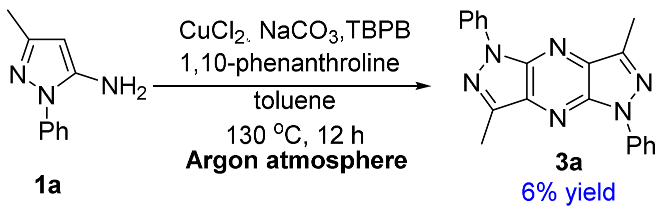

Scheme S12. The reaction with scheme 3 conditions under argon atmosphere

3-Methyl-1-phenyl-1*H*-pyrazol-5-amine (**1a**) (35 mg, 0.2 mmol, 1.0 equiv.), $CuCl_2$ (3.0 mg, 0.02 mmol, 20 mol%), 1,10-phenanthroline (5.4 mg, 0.03 mmol, 0.3 equiv.), tert-butyl peroxybenzoate (10 uL, 0.05 mmol, 0.5 equiv.), Na_2CO_3 (27 mg, 0.25 mmol, 2.5 equiv.) were added to a screw-cap test tube, and then solvent of toluene (2.0 mL) was added. The mixture was then stirred at 130 °C in oil bath for 12 h under argon atmosphere. After the reaction was completed (monitored by TLC), and 50 mL of water was added to the mixture, which was then extracted three times with CH_2Cl_2 (3×50 mL). The combined organic phase was dried with anhydrous Na_2SO_4, filtered and concentrated under reduced pressure. The crude residues were purified by column chromatography (silica gel: 200−300 mesh, solvent system: petroleum ether/ethyl acetate = 10:1) to obtain the corresponding products **3a** (2 mg ,6%).

7. Characterization of Products

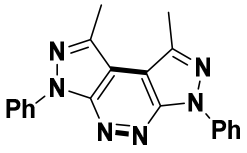

2a

1,8-Dimethyl-3,6-diphenyl-3,6-dihydrodipyrazolo[3,4-c:4',3'-e]pyridazine (2a)[3], (silica gel: 200−300 mesh, solvent system: petroleum ether/ethyl acetate = 5:1), 27 mg, 70%, yellow solid, m.p. 242-243 °C. ^{1}H-NMR (400 MHz, $CDCl_3$) δ 8.46 – 8.41 (m, 4H), 7.60 – 7.55 (m, 4H), 7.40 – 7.35 (m, 2H), 3.01 (s, 6H). ^{13}C-NMR (126 MHz, $CDCl_3$) δ 150.6, 140.3, 139.2, 129.2, 126.6, 121.6, 109.7, 15.4. HR-MS (ESI): calcd for $[M+H]^+$ $C_{20}H_{17}N_6$: 341.1509; found: 341.1505.

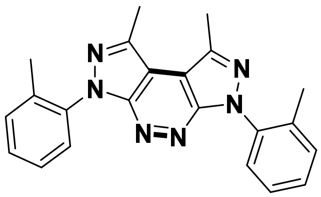

2b

1,8-Dimethyl-3,6-di-o-tolyl-3,6-dihydrodipyrazolo[3,4-*c*:4',3'-*e*]pyridazine (2b)[3], (silica gel: 200−300 mesh, solvent system: petroleum ether/ethyl acetate = 5:1), 22 mg, 60%, yellow solid, m.p. 220-221 °C. ^{1}H-NMR (400 MHz, $CDCl_3$) δ 7.50 (d, J = 7.2 Hz, 2H), 7.43 – 7.40 (m, 4H), 7.39 – 7.34 (m, 2H), 3.01 (s, 6H), 2.21 (s, 6H). ^{13}C-NMR (101 MHz, $CDCl_3$) δ 151.3, 140.1, 137.2, 135.5, 131.3, 129.2, 127.9, 126.6, 108.1, 18.4, 15.3. HR-MS (ESI): calcd for $[M+H]^+$ $C_{22}H_{21}N_6$: 369.1822; found: 369.1812.

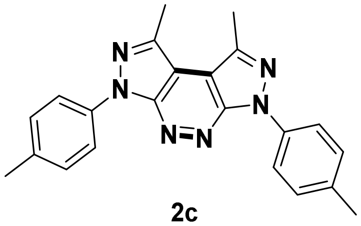

2c

1,8-Dimethyl-3,6-di-p-tolyl-3,6-dihydrodipyrazolo[3,4-c:4',3'-e]pyridazine (2c)[3], (silica gel: 200−300 mesh, solvent system: petroleum ether/ethyl acetate = 5:1), 21 mg, 58%, yellow solid, m.p. 272-273 °C. ^{1}H-NMR (500 MHz, $CDCl_3$) δ 8.27 (d, J = 8.5 Hz, 4H), 7.36 (d, J = 8.5 Hz, 4H), 2.98 (s, 6H), 2.44 (s, 6H). ^{13}C-NMR (126 MHz, $CDCl_3$) δ 150.4, 139.9, 136.8, 136.4, 129.8, 121.5, 109.4, 21.1, 15.4. HR-MS (ESI): calcd for $[M+H]^+$ $C_{22}H_{21}N_6$: 369.1822; found: 369.1816.

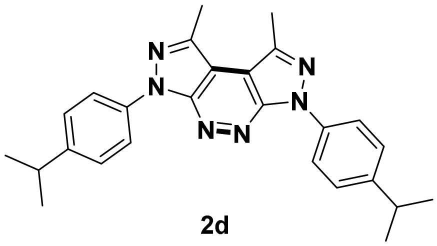

2d

3,6-Bis(4-isopropylphenyl)-1,8-dimethyl-3,6-dihydrodipyrazolo[3,4-*c*:4',3'-*e*] pyridazine (2d), (silica gel: 200−300 mesh, solvent system: petroleum ether/ethyl acetate = 5:1), 26 mg, 61%, orange solid, m.p. 227-228 °C. ^{1}H-NMR (500 MHz, $CDCl_3$) δ 8.29 (d, J = 8.5 Hz, 4H), 7.42 (d, J = 8.5 Hz, 4H), 3.05 – 3.00 (m, 2H), 2.99 (s, 6H), 1.33 (s, 6H), 1.31 (s, 6H). ^{13}C-NMR (101 MHz, $CDCl_3$) δ 150.5, 147.5, 140.0, 137.0, 127.2, 121.7, 109.4, 33.8, 24.0, 15.4. HR-MS (ESI): calcd for $[M+H]^+$ $C_{26}H_{29}N_6$: 425.2448; found: 425.2440.

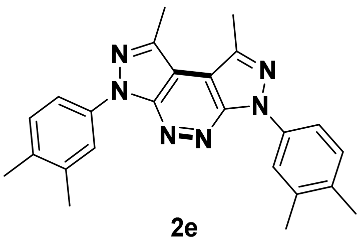

2e

3,6-Bis(3,4-dimethylphenyl)-1,8-dimethyl-3,6-dihydrodipyrazolo[3,4-*c*:4',3'-*e*] pyridazine (**2e**), (silica gel: 200−300 mesh, solvent system: petroleum ether/ethyl acetate = 5:1), 23 mg, 59%, yellow solid, m.p. 269-270 °C. ^{1}H-NMR (400 MHz, $CDCl_3$) δ 8.10 (d, J = 6.4 Hz, 4H), 7.31 (d, J = 8.8 Hz, 2H), 2.98 (s, 6H), 2.40 (s, 6H), 2.35 (s, 6H).^{13}C-NMR (101 MHz, $CDCl_3$) δ 150.5, 139.8, 137.6, 137.0, 135.2, 130.2, 122.8, 119.3, 109.3, 20.1, 19.4, 15.4. HR-MS (ESI): calcd for $[M+H]^+$ $C_{24}H_{25}N_6$: 397.2135; found: 397.2125.

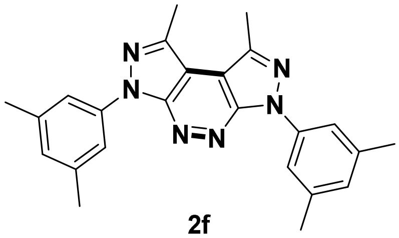

2f

3,6-Bis(3,5-dimethylphenyl)-1,8-dimethyl-3,6-dihydrodipyrazolo[3,4-*c*:4',3'-*e*] pyridazine (**2f**), (silica gel: 200−300 mesh, solvent system: petroleum ether/ethyl acetate = 5:1), 25 mg, 63%, yellow solid, m.p. 272-273 °C. ^{1}H-NMR (400 MHz, $CDCl_3$) δ 7.99 (s, 4H), 7.02 (s, 2H), 2.99 (s, 6H), 2.46 (s, 12H).^{13}C-NMR (101 MHz, $CDCl_3$) δ 150.5, 140.0, 139.0, 128.5, 119.6, 109.4, 29.7, 21.5, 15.4. HR-MS (ESI): calcd for $[M+H]^+$ $C_{24}H_{25}N_6$: 397.2135; found: 397.2134.

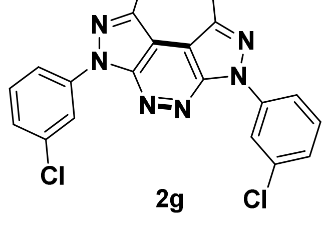

2g

3,6-Bis(3-chlorophenyl)-1,8-dimethyl-3,6-dihydrodipyrazolo[3,4-*c*:4',3'-*e*] pyridazine (**2g**), (silica gel: 200−300 mesh, solvent system: petroleum ether/ethyl acetate = 5:1), 29 mg, 70%, yellow solid, m.p. 251-252 °C. ^{1}H-NMR (400 MHz, $CDCl_3$) δ 8.50 – 8.44 (m, 4H), 7.50 (t, J = 8.0 Hz, 2H), 7.34 (ddd, J = 8.1, 2.0, 1.0 Hz, 2H), 2.99 (s, 6H). ^{13}C-NMR (126 MHz, $CDCl_3$) δ 150.7, 141.0, 140.1, 135.1, 130.3, 126.6, 121.3, 119.2, 110.1, 15.4. HR-MS (ESI): calcd for $[M+H]^+$ $C_{20}H_{15}Cl_2N_6$: 409.0730; found: 409.0723.

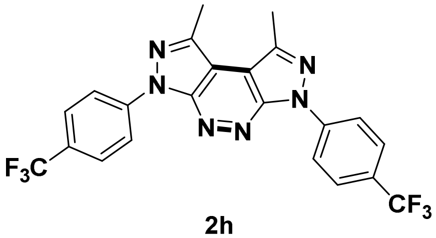

2h

1,8-Dimethyl-3,6-bis(4-(trifluoromethyl)phenyl)-3,6-dihydrodipyrazolo[3,4-*c*:4',3'-*e*] pyridazine (**2h**), (silica gel: 200−300 mesh, solvent system: petroleum ether/ethyl acetate = 5:1), 28 mg, 58%, yellow solid, m.p. 252-253 °C. ^{1}H-NMR (500 MHz, $CDCl_3$) δ 8.70 (d, J = 8.5 Hz, 4H), 7.83 (d, J = 8.5 Hz, 4H), 3.01 (s, 6H). ^{13}C-NMR (126 MHz, $CDCl_3$) δ 150.9, 141.8, 141.5, 126.5 (q, J_{CF} = 3.7 Hz), 120.8, 110.5, 15.5. ^{19}F NMR (470 MHz, $CDCl_3$) δ (ppm) -62.13. HR-MS (ESI): calcd for $[M+H]^+$ $C_{22}H_{15}F_6N_6$: 477.1257; found: 477.1249.

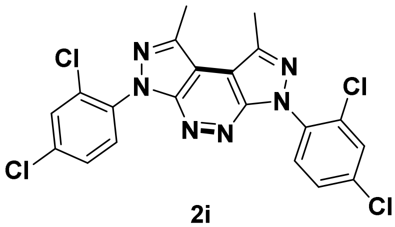

2i

3,6-Bis(2,4-dichlorophenyl)-1,8-dimethyl-3,6-dihydrodipyrazolo[3,4-*c*:4',3'-*e*] pyridazine (**2i**)[3], (silica gel: 200−300 mesh, solvent system: petroleum ether/ethyl acetate = 5:1), 33 mg, 70%, yellow solid, m.p. 199-200 °C. ^{1}H-NMR (500 MHz, $CDCl_3$) δ 7.65 (d, J = 2.5 Hz, 2H), 7.59 (d, J = 8.5 Hz, 2H), 7.45 (dd, J = 8.5, 2.0 Hz, 2H), 3.01 (s, 6H). ^{13}C-NMR (126 MHz, $CDCl_3$) δ 151.4, 141.5, 135.8, 134.4, 133.0, 130.6, 130.5,

127.9, 108.8, 15.4. HR-MS (ESI): calcd for $[M+H]^+$ $C_{20}H_{13}Cl_4N_6$: 476.9950; found: 476.9943.

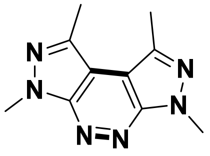

2j

1,3,6,8-Tetramethyl-3,6-dihydrodipyrazolo[3,4-*c*:4',3'-*e*] pyridazine (**2j**)[3], (silica gel: 200−300 mesh, solvent system: petroleum ether/ethyl acetate = 5:1), 11 mg, 50%, yellow solid, m.p. 106-107°C. ^{1}H-NMR (500 MHz, $CDCl_3$) δ 4.39 (s, 6H), 2.84 (s, 6H).^{13}C-NMR (126 MHz, $CDCl_3$) δ 150.6, 138.0, 107.4, 35.1, 14.9. HR-MS (ESI): calcd for $[M+H]^+$ $C_{10}H_{13}N_6$: 217.1196; found: 217.1188.

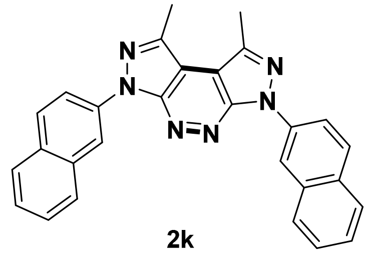

2k

1,8-Dimethyl-3,6-di(naphthalen-2-yl)-3,6-dihydrodipyrazolo[3,4-*c*:4',3'-*e*] pyridazine (**2k**), (silica gel: 200−300 mesh, solvent system: petroleum ether/ethyl acetate = 5:1), 30 mg, 68%, yellow solid, m.p. 242-243 °C. ^{1}H-NMR (400 MHz, $CDCl_3$) δ 8.96 (d, J = 2.4 Hz, 2H), 8.59 (dd, J = 9.2, 2.4 Hz, 2H), 8.05 – 7.99 (m, 4H), 7.90 (d, J = 8.0 Hz, 2H), 7.57 – 7.49 (m, 4H), 3.01 (s, 6H).^{13}C-NMR (126 MHz, $CDCl_3$) δ 150.8, 140.5, 136.8, 133.6, 131.9, 129.2, 128.4, 127.7, 126.7, 126.0, 120.4, 119.0, 109.8, 15.5. HR-MS (ESI): calcd for $[M+H]^+$ $C_{28}H_{21}N_6$: 441.1822; found: 441.1815.

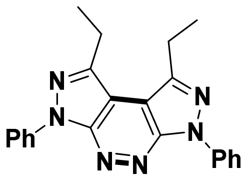

2l

1,8-Diethyl-3,6-diphenyl-3,6-dihydrodipyrazolo[3,4-*c*:4',3'-*e*] pyridazine (2l), (silica gel: 200−300 mesh, solvent system: petroleum ether/ethyl acetate = 5:1), 29 mg, 78%, yellow solid, m.p. 246-247 °C. ^{1}H-NMR (500 MHz, $CDCl_3$) δ 8.45 (d, J = 7.5 Hz, 4H), 7.57 (t, J = 7.5 Hz, 4H), 7.37 (t, J = 7.5 Hz, 2H), 3.37 (q, J = 7.5 Hz, 4H), 1.58 (d, J = 7.5 Hz, 6H).^{13}C-NMR (126 MHz, $CDCl_3$) δ 150.5, 145.6, 139.3, 129.2, 126.5, 121.6, 108.9, 22.8, 13.4. HR-MS (ESI): calcd for $[M+H]^+$ $C_{22}H_{21}N_6$: 369.1822; found: 369.1817.

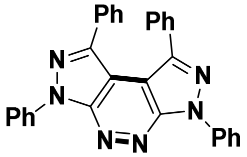

2m

1,3,6,8-Tetraphenyl-3,6-dihydrodipyrazolo[3,4-*c*:4',3'-*e*] pyridazine (2m)[3], (silica gel: 200−300 mesh, solvent system: petroleum ether/ethyl acetate = 5:1), 35 mg, 76%, orange solid, m.p. 217-218 °C. ^{1}H-NMR (400 MHz, $CDCl_3$) δ 8.54 – 8.50 (m, 4H), 7.64 – 7.59 (m, 4H), 7.43 (t, J = 7.6 Hz, 2H), 7.37 (d, J = 6.8 Hz, 4H), 7.21 (t, J = 7.6 Hz, 2H), 7.03 (t, J = 8.0 Hz, 4H).^{13}C-NMR (101 MHz, $CDCl_3$) δ 151.1, 144.7, 139.1, 132.3, 129.3, 128.6, 127.8, 127.2, 122.3, 108.2. HR-MS (ESI): calcd for $[M+H]^+$ $C_{30}H_{21}N_6$: 465.1822; found: 465.1813.

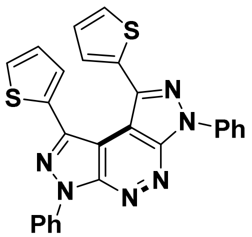

2n

3,6-Diphenyl-1,8-di(thiophen-2-yl)-3,6-dihydrodipyrazolo[3,4-*c*:4',3'-*e*] pyridazine (2n), (silica gel: 200−300 mesh, solvent system: petroleum ether/ethyl acetate = 5:1), 28 mg, 58%, orange solid, m.p. 253-254 °C. ^{1}H-NMR (500 MHz, $CDCl_3$) δ 8.46 (d, J = 8.0 Hz, 4H), 7.61 (t, J = 8.0 Hz, 4H), 7.44 (t, J = 7.5 Hz, 2H), 7.32 (d, J = 5.0 Hz, 2H), 6.82 (d, J = 3.5 Hz, 2H), 6.80 – 6.78 (m, 2H).^{13}C-NMR (126 MHz, $CDCl_3$) δ 150.8, 138.8, 138.6, 133.0, 129.3, 129.0, 127.5, 127.4, 126.8, 122.5, 108.4. HR-MS (ESI): calcd for $[M+H]^+$ $C_{26}H_{17}N_6S_2$: 477.0951; found: 477.0945.

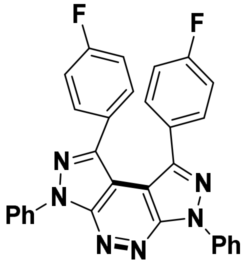

2o

1,8-Bis(4-fluorophenyl)-3,6-diphenyl-3,6-dihydrodipyrazolo[3,4-*c*:4',3'-*e*] pyridazine (**2o**), (silica gel: 200−300 mesh, solvent system: petroleum ether/ethyl acetate = 5:1), 32 mg, 64%, orange solid, m.p. 254-255 °C. ^{1}H-NMR (400 MHz, $CDCl_3$) δ 8.51 – 8.47 (m, 4H), 7.64 – 7.59 (m, 4H), 7.47 – 7.42 (m, 2H), 7.37 – 7.32 (m, 4H), 6.84 – 6.77 (m, 4H).^{13}C-NMR (101 MHz, $CDCl_3$) δ 164.6, 162.1, 151.0, 143.5, 139.0, 130.5 (d, J_{CF} = 8.4 Hz), 128.4, 127.4, 122.3, 114.9 (d, J_{CF} = 21.9 Hz), 108.0. ^{19}F NMR (470 MHz, $CDCl_3$) δ (ppm) -112.51. HR-MS (ESI): calcd for $[M+H]^+$ $C_{30}H_{19}F_2N_6$: 501.1634; found: 501.1626.

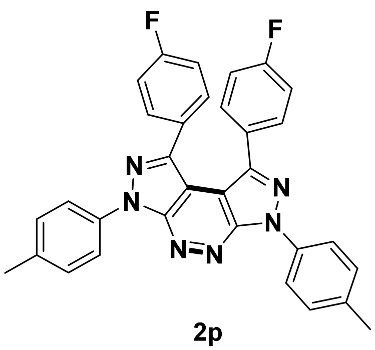

2p

1,8-Bis(4-fluorophenyl)-3,6-di-p-tolyl-3,6-dihydrodipyrazolo[3,4-*c*:4',3'-*e*] pyridazine (**2p**)**,** (silica gel: 200−300 mesh, solvent system: petroleum ether/ethyl acetate = 5:1), 32 mg, 60%, orange solid, m.p. 253-254 °C. ^{1}H-NMR (500 MHz, $CDCl_3$) δ 8.33 (d, J = 8.5 Hz, 4H), 7.40 (d, J = 8.5 Hz, 4H), 7.33 (dd, J = 8.5, 5.5 Hz, 4H), 6.79 (t, J = 8.5 Hz, 4H), 2.47 (s, 6H).^{13}C-NMR (126 MHz, $CDCl_3$) δ 164.3, 162.3, 150.8, 143.1, 137.3, 136.6, 130.4 (d, J_{CF} = 8.3 Hz), 128.5 (d, J_{CF} = 3.2 Hz), 122.2, 115.0, 114.8 (d, J_{CF} = 22.0 Hz), 107.8, 21.1. ^{19}F NMR (470 MHz, $CDCl_3$) δ (ppm) -112.67. HR-MS (ESI): calcd for $[M+H]^+$ $C_{32}H_{23}F_2N_6$: 529.1947; found: 529.1939.

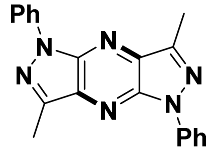

3a

3,7-Dimethyl-1,5-diphenyl-1,5-dihydrodipyrazolo[3,4-*b*:3',4'-*e*] pyrazine (**3a**), (silica gel: 200−300 mesh, solvent system: petroleum ether/ethyl acetate = 10:1), 20 mg, 59%, orange solid, m.p. 276-277 °C. ^{1}H-NMR (500 MHz, $CDCl_3$) δ 8.40 (d, J = 8.0 Hz, 4H), 7.57 (t, J = 8.0 Hz, 4H), 7.32 (t, J = 7.5 Hz, 2H), 2.84 (s, 6H).^{13}C-NMR (126 MHz, $CDCl_3$) δ 143.1, 142.3, 139.5, 134.1, 129.2, 125.6, 119.8, 11.6. HR-MS (ESI): calcd for $[M+H]^+$ $C_{20}H_{17}N_6$: 341.1509; found: 341.1501.

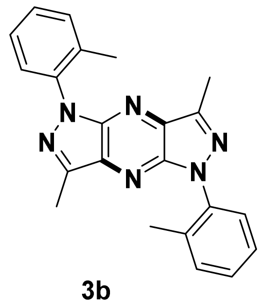

3b

3,7-Dimethyl-1,5-di-o-tolyl-1,5-dihydrodipyrazolo[3,4-*b*:3',4'-*e*] pyrazine (**3b**), (silica gel: 200−300 mesh, solvent system: petroleum ether/ethyl acetate = 10:1), 19

mg, 52%, yellow solid, m.p. 234-235 °C. ^{1}H-NMR (400 MHz, $CDCl_3$) δ 7.50 – 7.47 (m, 2H), 7.44 (td, J = 7.1, 6.6, 2.1 Hz, 4H), 7.41 – 7.36 (m, 2H), 2.74 (s, 6H), 2.28 (s, 6H).^{13}C-NMR (101 MHz, $CDCl_3$) δ 143.3, 142.6, 137.3, 135.5, 133.3, 131.4, 128.7, 127.7, 126.7, 18.7, 11.7. HR-MS (ESI): calcd for $[M+H]^+$ $C_{22}H_{21}N_6$: 369.1822; found: 369.1816.

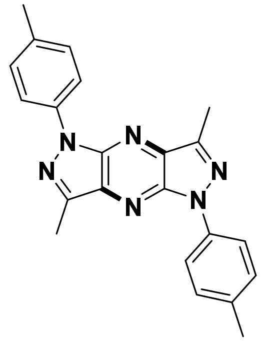

3c

3,7-Dimethyl-1,5-di-p-tolyl-1,5-dihydrodipyrazolo[3,4-*b*:3',4'-*e*] pyrazine (**3c**), (silica gel: 200−300 mesh, solvent system: petroleum ether/ethyl acetate = 10:1), 17 mg, 46%, yellow solid, m.p. 273-274 °C. Due to the low solubility of the product, 0.6 mL of $CDCl_3$/TFA (v:v = 20:1) was used to measure the NMR spectrum. ^{1}H-NMR (400 MHz, $CDCl_3$/TFA=20:1(v:v)) δ 7.71 (d, J = 8.4 Hz, 4H), 7.43 (d, J = 8.0 Hz, 4H), 2.87 (s, 6H), 2.48 (s, 6H).^{13}C-NMR (101 MHz, $CDCl_3$/TFA=20:1(v:v)) δ 162.3 (q, J_{CF}=43.8 Hz, CO), 143.40, 142.11, 139.87, 133.84, 133.62, 130.53, 124.03, 114.3 (q, J_{CF}=285.6 Hz, CF_3), 20.95, 10.76. HR-MS (ESI): calcd for $[M+H]^+$ $C_{22}H_{21}N_6$: 369.1822; found: 369.1813.

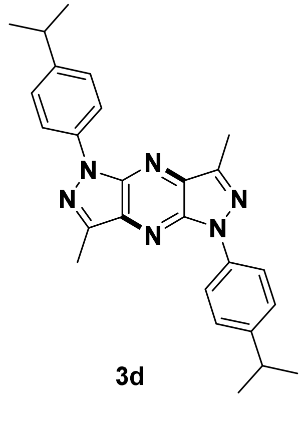

3d

1,5-Bis(4-isopropylphenyl)-3,7-dimethyl-1,5-dihydrodipyrazolo[3,4-*b*:3',4'-*e*] pyrazine (**3d**), (silica gel: 200−300 mesh, solvent system: petroleum ether/ethyl acetate = 10:1), 19 mg, 45%, yellow solid, mp 236-237 °C. ^{1}H-NMR (400 MHz, $CDCl_3$) δ 8.26 (d, J = 8.6 Hz, 4H), 7.42 (d, J = 8.6 Hz, 4H), 3.00 (p, J = 6.9 Hz, 2H), 2.82 (s, 6H), 1.33 (d, J = 6.9 Hz, 12H). ^{13}C-NMR (101 MHz, $CDCl_3$) δ 146.3, 142.6, 142.1, 137.3, 133.9, 127.2, 120.0, 33.8, 24.1, 11.6. HR-MS (ESI): calcd for $[M+H]^+$ $C_{26}H_{29}N_6$: 425.2448; found: 425.2441.

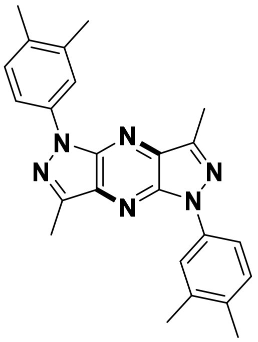

3e

1,5-Bis(3,4-dimethylphenyl)-3,7-dimethyl-1,5-dihydrodipyrazolo[3,4-*b*:3',4'-*e*] pyrazine (**3e**), (silica gel: 200−300 mesh, solvent system: petroleum ether/ethyl acetate = 10:1), 23mg, 59%, yellow solid, m.p. 251-252 °C. Due to the low solubility of the product, 0.6 mL of $CDCl_3$/TFA (v:v = 20:1) was used to measure the NMR spectrum. ^{1}H-NMR (500 MHz, $CDCl_3$/TFA=20:1(v:v)) δ 7.55 (d, J = 8.8 Hz, 1H), 7.37 (d, J = 8.4 Hz, 1H), 2.87 (s, 1H), 2.38 (s, 3H). ^{13}C-NMR (126 MHz, $CDCl_3$/TFA=20:1(v:v)) δ 162.2 (q, J_{CF}=44.1 Hz, CO), 143.1, 142.0, 138.8, 138.5, 133.7, 130.9, 125.1, 121.5, 114.3 (q, J_{CF}=285.4 Hz, CF_3), 19.7, 19.4, 10.8. HR-MS (ESI): calcd for $[M+H]^+$ $C_{24}H_{25}N_6$: 397.2135; found: 397.2125.

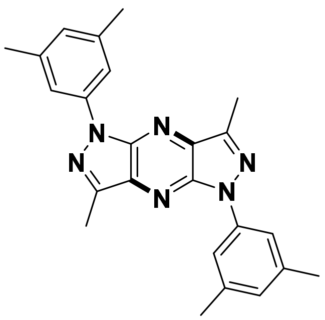

3f

1,5-Bis(3,5-dimethylphenyl)-3,7-dimethyl-1,5-dihydrodipyrazolo[3,4-*b*:3',4'-*e*] pyrazine (**3f**), (silica gel: 200−300 mesh, solvent system: petroleum ether/ethyl acetate = 10:1), 21 mg, 52%, yellow solid, m.p. 254-255 °C. ^{1}H-NMR (400 MHz, $CDCl_3$) δ 7.98 (s, 4H), 6.97 (s, 2H), 2.83 (s, 6H), 2.47 (s, 12H). ^{13}C-NMR (101 MHz, $CDCl_3$) δ 153.3, 142.7, 142.2, 139.3, 139.0, 134.0, 127.4, 117.9, 21.6, 11.6. HR-MS (ESI): calcd for $[M+H]^+$ $C_{24}H_{25}N_6$: 397.2135; found: 397.2123.

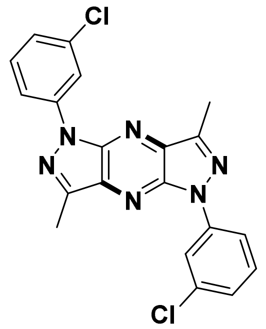

3g

1,5-Bis(3-chlorophenyl)-3,7-dimethyl-1,5-dihydrodipyrazolo[3,4-*b*:3',4'-*e*] pyrazine (**3g**), (silica gel: 200−300 mesh, solvent system: petroleum ether/ethyl acetate = 10:1), 16 mg, 40%, yellow solid, m.p. 238-239°C. ^{1}H-NMR (500 MHz, $CDCl_3$) δ 8.47 (s, 2H), 8.39 (d, J = 8.5 Hz, 2H), 7.49 (t, J = 8.0 Hz, 2H), 7.28 (d, J = 10.0 Hz, 2H), 2.84 (s, 6H). ^{13}C-NMR (126 MHz, $CDCl_3$) δ 143.8, 142.3, 140.4, 135.0, 134.2, 130.3, 125.5, 119.5, 117.3, 11.6. HR-MS (ESI): calcd for $[M+H]^+$ $C_{20}H_{15}Cl_2N_6$: 397.2135; found: 397.2130.

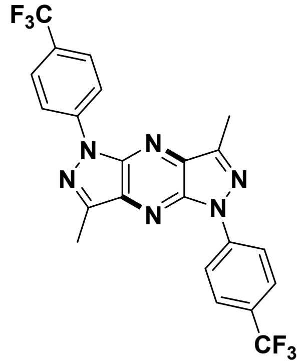

3h

3,7-Dimethyl-1,5-bis(4-(trifluoromethyl)phenyl)-1,5-dihydrodipyrazolo[3,4-*b*:3',4'-*e*] pyrazine (**3h**), (silica gel: 200−300 mesh, solvent system: petroleum ether/ethyl acetate = 10:1), 26 mg, 54%, yellow solid, m.p. 252-253 °C. Due to the low solubility of the product, 0.6 mL of $CDCl_3$/TFA (v:v = 20:1) was used to measure the NMR spectrum. ^{1}H-NMR (500 MHz, $CDCl_3$/TFA=20:1(v:v)) δ 8.34 (d, J = 8.0 Hz, 4H), 7.88 (d, J = 8.5 Hz, 4H), 2.90 (s, 6H).^{13}C-NMR (126 MHz, $CDCl_3$/TFA=20:1(v:v)) δ 162.2 (q, J_{CF}=44.0 Hz, CO), 145.1, 142.6, 140.6, 134.5, 126.9 (q, J_{CF} = 3.7 Hz), 121.2, 114.2 (q, J_{CF}=284.8Hz, CF_3), 11.2. ^{19}F NMR (470 MHz, $CDCl_3$) δ (ppm) -62.13. HR-MS (ESI): calcd for $[M+H]^+$ $C_{22}H_{15}F_6N_6$: 477.1257; found: 477.1249.

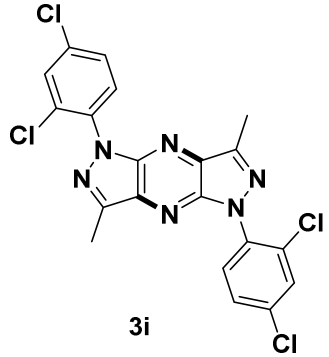

3i

1,5-Bis(2,4-dichlorophenyl)-3,7-dimethyl-1,5-dihydrodipyrazolo[3,4-*b*:3',4'-*e*] pyrazine (**3i**), (silica gel: 200−300 mesh, solvent system: petroleum ether/ethyl acetate = 10:1), 22 mg, 46%, yellow solid, m.p. 256-257 °C. Due to the low solubility of the product, 0.6 mL of $CDCl_3$/TFA (v:v = 20:1) was used to measure the NMR spectrum. ^{1}H-NMR (500 MHz, $CDCl_3$/TFA=20:1(v:v)) δ 7.69 (d, J = 2.5 Hz, 2H), 7.55 (d, J = 8.0 Hz, 2H), 7.51 (dd, J = 8.5, 2.5 Hz, 2H), 2.80 (s, 6H). ^{13}C NMR (126 MHz,

$CDCl_3$/TFA=20:1(v:v)) δ 161.7 (q, J_{CF}=43.5Hz, CO), 144.5, 143.1, 137.4, 133.8, 133.4, 132.3, 130.8, 130.8, 128.4, 114.2(q, J_{CF}=284.8Hz, CF_3), 11.2. HR-MS (ESI): calcd for $[M+H]^+$ $C_{20}H_{13}Cl_4N_6$ $[M+H]^+$: 476.9950; found: 476.9945.

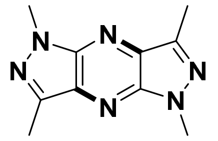

3j

1,3,5,7-Tetramethyl-1,5-dihydrodipyrazolo[3,4-*b*:3',4'-*e*] pyrazine (**3j**), (silica gel: 200−300 mesh, solvent system: petroleum ether/ethyl acetate = 10:1), 12 mg, 57%, yellow solid, m.p. 216-217 °C. ^{1}H-NMR (500 MHz, $CDCl_3$) δ 4.16 (s, 6H), 2.72 (s, 6H).^{13}C-NMR (126 MHz, $CDCl_3$) δ 142.9, 140.1, 132.6, 34.1, 11.5. HR-MS (ESI): calcd for $[M+H]^+$ $C_{10}H_{13}N_6$ $[M+H]^+$: 217.1196; found: 217.1190.

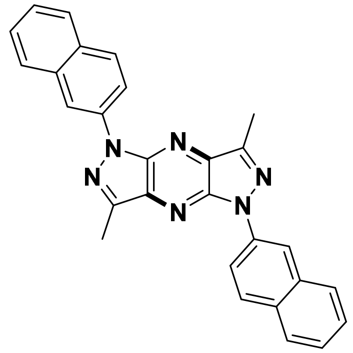

3k

3,7-Dimethyl-1,5-di(naphthalen-2-yl)-1,5-dihydrodipyrazolo[3,4-*b*:3',4'-*e*] pyrazine (**3k**), (silica gel: 200−300 mesh, solvent system: petroleum ether/ethyl acetate = 10:1), 26 mg, 60%, orange solid, m.p. 252-253 °C. Due to the low solubility of the product, 0.6 mL of $CDCl_3$/TFA (v:v = 20:1) was used to measure the NMR spectrum. ^{1}H-NMR (500 MHz, $CDCl_3$/TFA=20:1(v:v)) δ 8.45 (s, 2H), 8.10 (d, J = 1.5 Hz, 4H), 7.97 (ddd, J = 12.0, 7.6, 2.0 Hz, 4H), 7.61 (m, 4H), 2.93 (s, 6H).^{13}C-NMR (126 MHz, $CDCl_3$/TFA=20:1(v:v)) δ 162.1(q, J_{CF}=42.8 Hz, CO), 144.01, 142.41, 134.29, 134.13, 133.45, 132.68, 129.99, 128.27, 128.01, 127.45, 127.09, 121.35, 120.98, 114.2(q,

J_{CF}=284.8 Hz, CF_3), 11.01 . HR-MS (ESI): calcd for $[M+H]^+$ $C_{28}H_{21}N_6$ $[M+H]^+$: 441.1822; found: 441.1816.

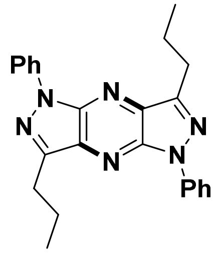

3l

1,5-Diphenyl-3,7-dipropyl-1,5-dihydrodipyrazolo[3,4-*b*:3',4'-*e*] pyrazine (**3l**), (silica gel: 200−300 mesh, solvent system: petroleum ether/ethyl acetate = 10:1), 24 mg, 60%, yellow solid, m.p. 228-229 °C. ^{1}H-NMR (500 MHz, $CDCl_3$) δ 8.42 (d, *J* = 7.4 Hz, 4H), 7.59 – 7.55 (m, 4H), 7.31 (t, *J* = 7.5 Hz, 2H), 3.23 (t, *J* = 7.5 Hz, 4H), 2.07 (m, 4H), 1.12 (t, *J* = 7.5 Hz, 6H).^{13}C-NMR (126 MHz, $CDCl_3$) *δ* 146.8, 142.3, 139.6, 133.8, 129.2, 125.4, 119.7, 28.5, 21.6, 14.1. HR-MS (ESI): calcd for $[M+H]^+$ $C_{24}H_{25}N_6$: 397.2135; found: 397.2127.

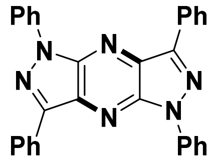

3m

1,3,5,7-Tetraphenyl-1,5-dihydrodipyrazolo[3,4-*b*:3',4'-*e*] pyrazine (**3m**), (silica gel: 200−300 mesh, solvent system: petroleum ether/ethyl acetate = 10:1), 27 mg, 58%, orange solid, m.p. 275-276 °C. Due to the low solubility of the product, 0.6 mL of $CDCl_3$/TFA (v:v = 20:1) was used to measure the NMR spectrum. ^{1}H-NMR (400 MHz, $CDCl_3$/TFA=20:1 (v:v)) *δ* 8.45 (d, *J* = 6.4 Hz, 4H), 8.17 (d, *J* = 7.6 Hz, 4H), 7.71 – 7.51 (m, 12H).^{13}C-NMR (101 MHz, $CDCl_3$/TFA=20:1 (v:v)) *δ* 162.7(q, J_{CF}=43.4 Hz, CO), 158.1, 137.3, 130.8, 129.9, 129.5, 128.63, 128.1, 123.2, 114.4(q, J_{CF}=285.8 Hz, CF_3). HR-MS (ESI): calcd for $[M+H]^+$ $C_{30}H_{21}N_6$: 465.1822; found: 465.1816.

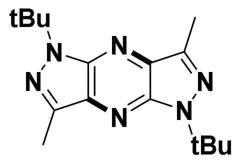

3q

1,5-Di-tert-butyl-3,7-dimethyl-1,5-dihydrodipyrazolo[3,4-*b*:3',4'-*e*] pyrazine (3q), (silica gel: 200−300 mesh, solvent system: petroleum ether/ethyl acetate = 10:1), 12 mg, 40%, yellow solid, m.p. 228-229 °C. ^{1}H-NMR (400 MHz, $CDCl_3$) δ 2.68 (s, 6H), 1.85 (s, 18H).^{13}C-NMR (101 MHz, $CDCl_3$) δ 142.3, 138.8, 132.7, 59.9, 29.1, 11.3. HR-MS (ESI): calcd for $[M+H]^+$ $C_{16}H_{25}N_6$: 301.2135; found: 301.2134.

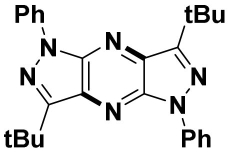

3r

3,7-Di-tert-butyl-1,5-diphenyl-1,5-dihydrodipyrazolo[3,4-*b*:3',4'-*e*] pyrazine (3r), (silica gel: 200−300 mesh, solvent system: petroleum ether/ethyl acetate = 10:1), 19 mg, 45%, yellow solid, m.p. 252-253 °C. ^{1}H-NMR (400 MHz, $CDCl_3$) δ 8.49 – 8.44 (m, 4H), 7.59 – 7.54 (m, 4H), 7.29 (m, 2H), 1.72 (s, 18H).^{13}C-NMR (101 MHz, $CDCl_3$) δ 153.1, 142.0, 139.9, 132.4, 129.1, 125.1, 119.4, 34.3, 29.2. HR-MS (ESI): calcd for $[M+H]^+$ $C_{26}H_{29}N_6$: 425.2448; found: 425.2441.

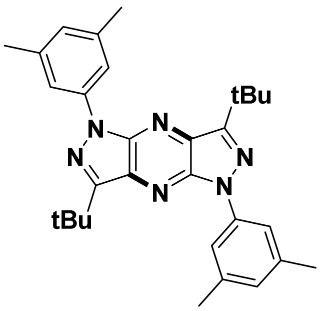

3s

3,7-Di-tert-butyl-1,5-bis(3,5-dimethylphenyl)-1,5-dihydrodipyrazolo[3,4-*b*:3',4'-*e*] pyrazine (3s), (silica gel: 200−300 mesh, solvent system: petroleum ether/ethyl acetate = 10:1), 20 mg, 42%, orange solid, m.p. 262-263 °C. ^{1}H-NMR (500 MHz, $CDCl_3$) δ

8.10 (s, 4H), 6.94 (s, 2H), 2.47 (s, 12H), 1.72 (s, 18H).^{13}C-NMR (126 MHz, $CDCl_3$) δ 152.8, 141.9, 139.7, 138.8, 132.2, 126.8, 117.3, 34.2, 29.1, 21.7. HR-MS (ESI): calcd for $[M+H]^+$ $C_{30}H_{37}N_6$: 481.3074; found: 481.3070.

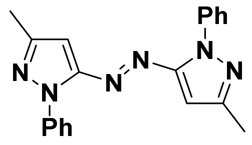

4a

(*E*)-1,2-bis(3-methyl-1-phenyl-1*H*-pyrazol-5-yl)diazene (4a), (silica gel: 200−300 mesh, solvent system: petroleum ether/ethyl acetate = 8:1), 14 mg, 40%,orange solid; m.p. 198-199 °C. ^{1}H-NMR (500 MHz, $CDCl_3$) δ 7.70 (d, J = 7.5 Hz, 4H), 7.50 (t, J = 7.5 Hz, 4H), 7.40 (t, J = 7.5 Hz, 2H), 6.40 (s, 2H), 2.39 (s, 6H).^{13}C-NMR (126 MHz, $CDCl_3$) δ 154.3, 150.0, 139.0, 128.8, 127.5, 124.8, 94.6, 14.0. HR-MS (ESI): calcd for $[M+H]^+$ $C_{20}H_{19}N_6$: 343.1666; found: 343.1663.

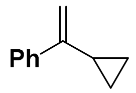

5

(1-Cyclopropylvinyl)benzene (5), (silica gel: 200−300 mesh, solvent system: petroleum ether), 0.68 g, 94%, clear liquid. ^{1}H-NMR (500 MHz, $CDCl_3$) δ 7.58 (d, J = 6.0 Hz, 2H), 7.31 (t, J = 7.0 Hz, 2H), 7.26 (d, J = 7.5 Hz, 1H), 5.26 (s, 1H), 4.92 (s, 1H), 1.63 (m, 1H), 0.83 – 0.79 (m, 2H), 0.58 (dd, J = 5.5, 2.2 Hz, 2H).^{13}C-NMR (126 MHz, $CDCl_3$) δ 149.3, 141.6, 128.1, 127.4, 126.1, 109.0, 15.6, 6.6. HR-MS (ESI): calcd for $[M+H]^+$ $C_{11}H_{13}$: 145.1012; found: 145.1008.

8. References

1. Cao, J.; Lv, D.; Yu, F.; Chiou, M.-F.; Li, Y.; Bao, H., Regioselective Three-Component Synthesis of Vicinal Diamines via 1,2-Diamination of Styrenes. *Org. Lett.* **2021***, 23,* 3184−3189.

2. Jiang, B.; Ning, Y.; Fan, W.; Tu, S.-J.; Li, G., Oxidative Dehydrogenative Couplings of Pyrazol-5-amines Selectively Forming Azopyrroles. *J. Org. Chem.* **2014***, 79,* 4018−4024.

3. Rastogi, G. K.; Deb, M. L.; Baruah, P. K., Copper-catalysed dehydrogenative self-coupling/cyclization of 5-aminopyrazoles: synthesis and photophysical study of pyridazines. *Chem. Commun.* **2023**, *59,* 9642–9645.

4. (a) Yan, M.; Zhu, L.; Zhang, X.; Yin, S.-F.; Kambe, N.; Qiu, R., Nickel-Catalyzed N, N-Diarylation of 8-Aminoquinoline with Large Steric Aryl Bromides and Fluorescence of Products. *Org. Lett.* **2021**, *23,* 2514−2520. (b) Yang, T.; Cao, X.; Zhang, X.-X.; Ou, Y.; Au, C.-T.; Yin, S.-F.; Qiu, R., Iodine-Catalyzed Synthesis of N, N′-Chelate Organoboron Aminoquinolate. *J. Org. Chem.* **2020**, *85,* 12430−12443.

9. Copy of ^{1}H and ^{13}C NMR Spectra of Products

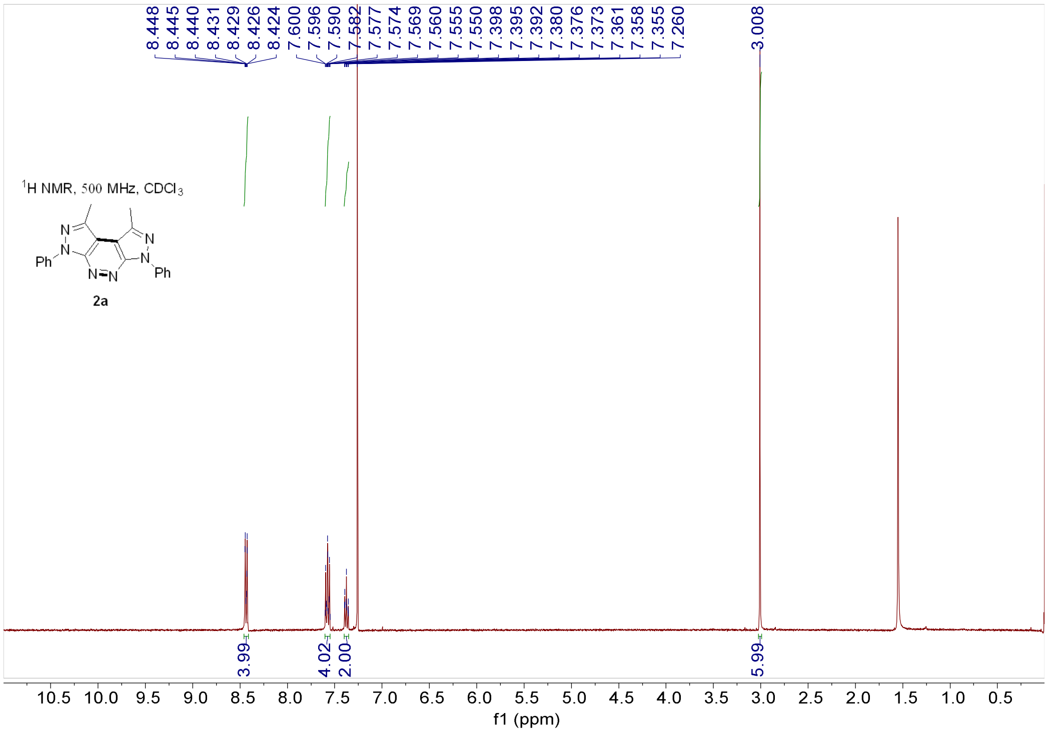

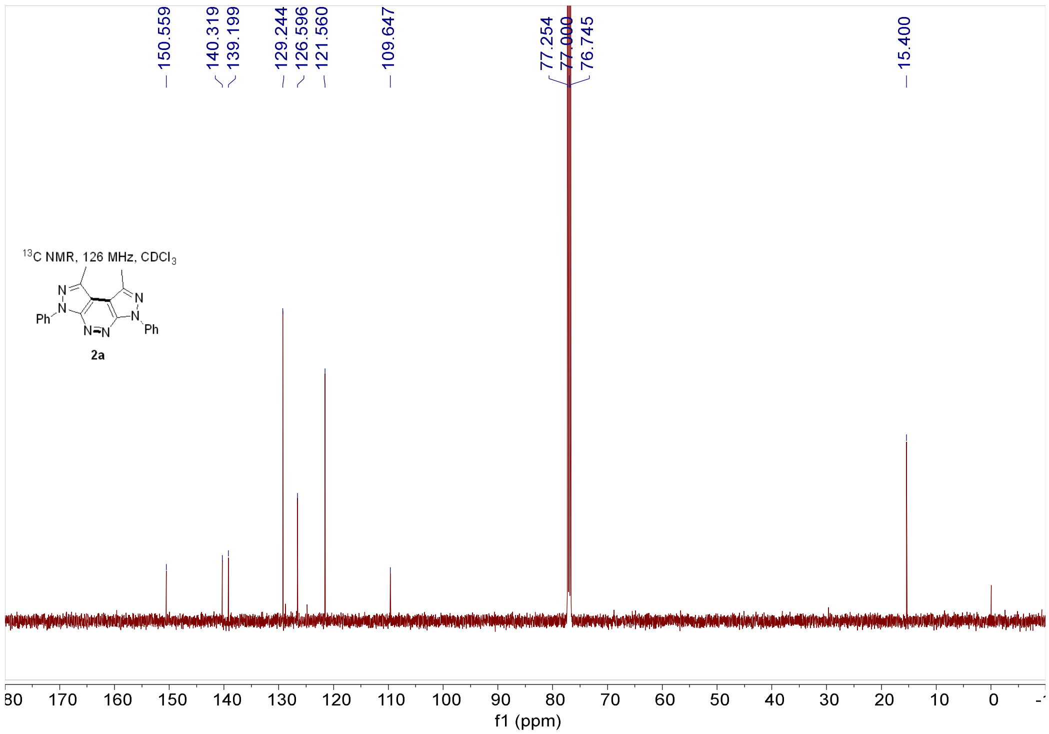

150.559
140.319
139.199
129.244
126.596
121.560
109.647
77.254
77.000
76.745
15.400
13C NMR, 126 MHz, CDCl3
2a
f1 (ppm)

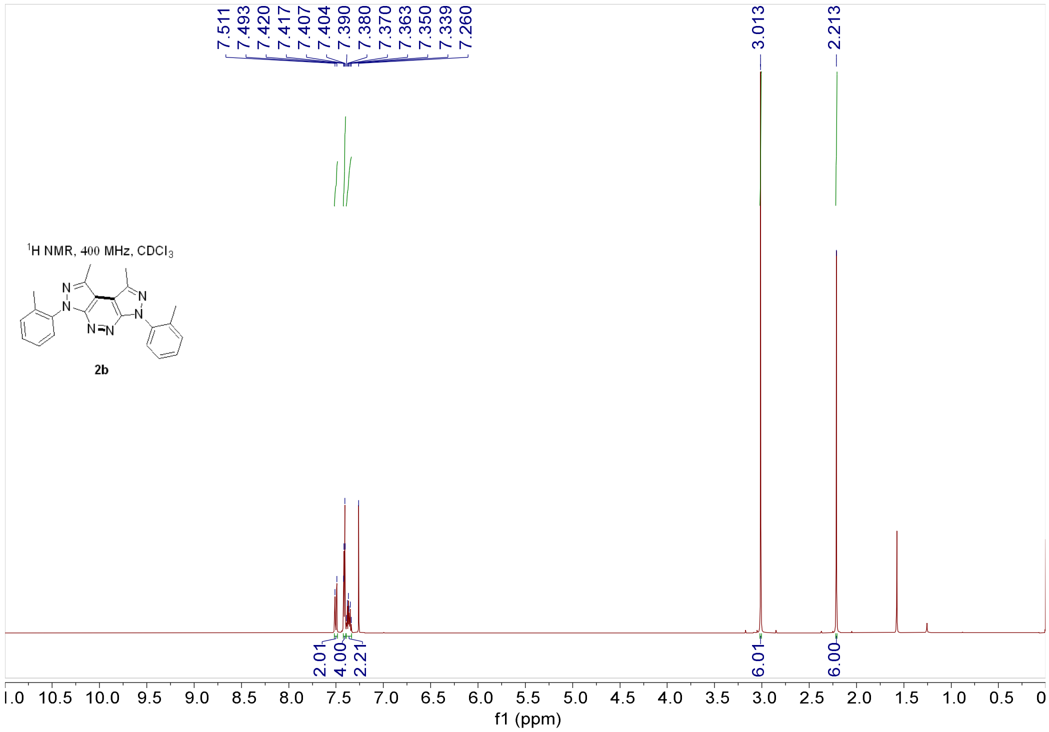

7.511
7.493
7.420
7.417
7.407
7.404
7.390
7.380
7.370
7.363
7.350
7.339
7.260
3.013
2.213
1H NMR, 400 MHz, CDCl3
2b
2.01
4.00
2.21
6.01
6.00
f1 (ppm)

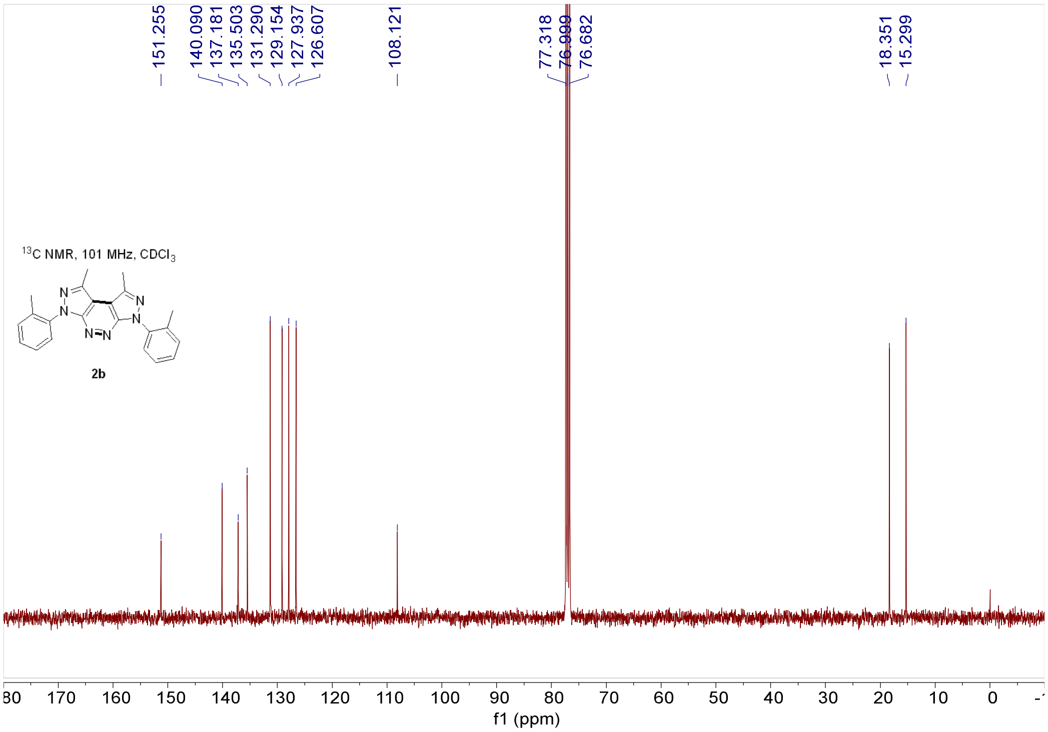
151.255
140.090
137.181
135.503
131.290
129.154
127.937
126.607
108.121
77.318
76.999
76.682
18.351
15.299
^{13}C NMR, 101 MHz, $CDCl_3$
2b
f1 (ppm)

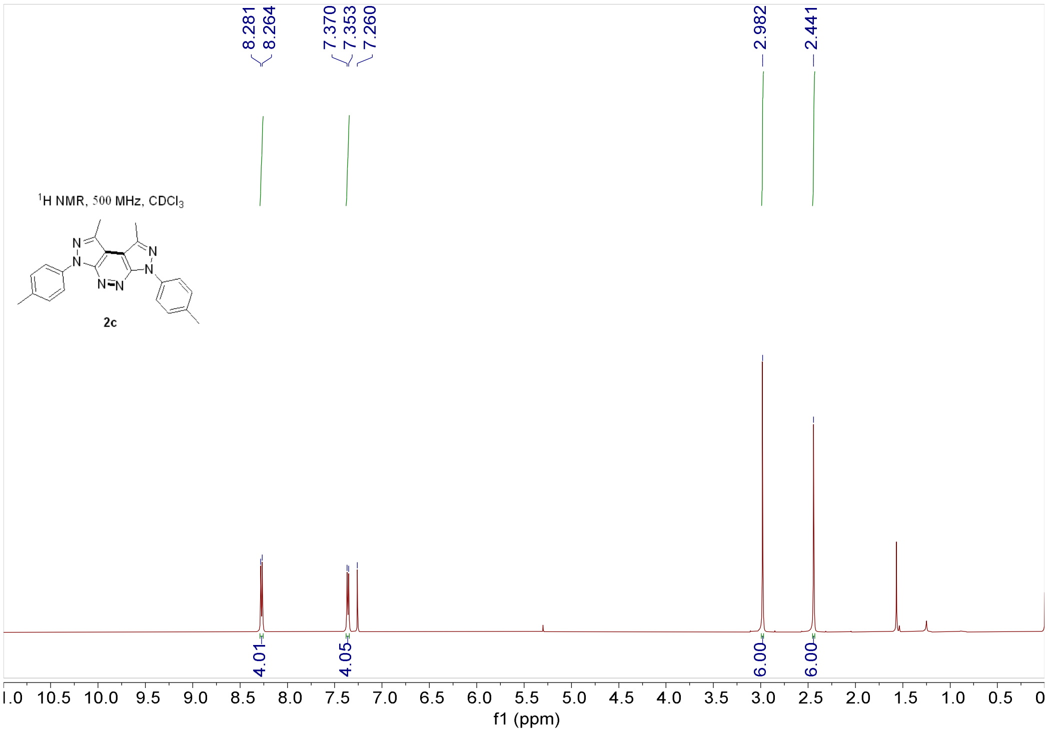
8.281
8.264
7.370
7.353
7.260
2.982
2.441
^{1}H NMR, 500 MHz, $CDCl_3$
2c
4.01
4.05
6.00
6.00
f1 (ppm)

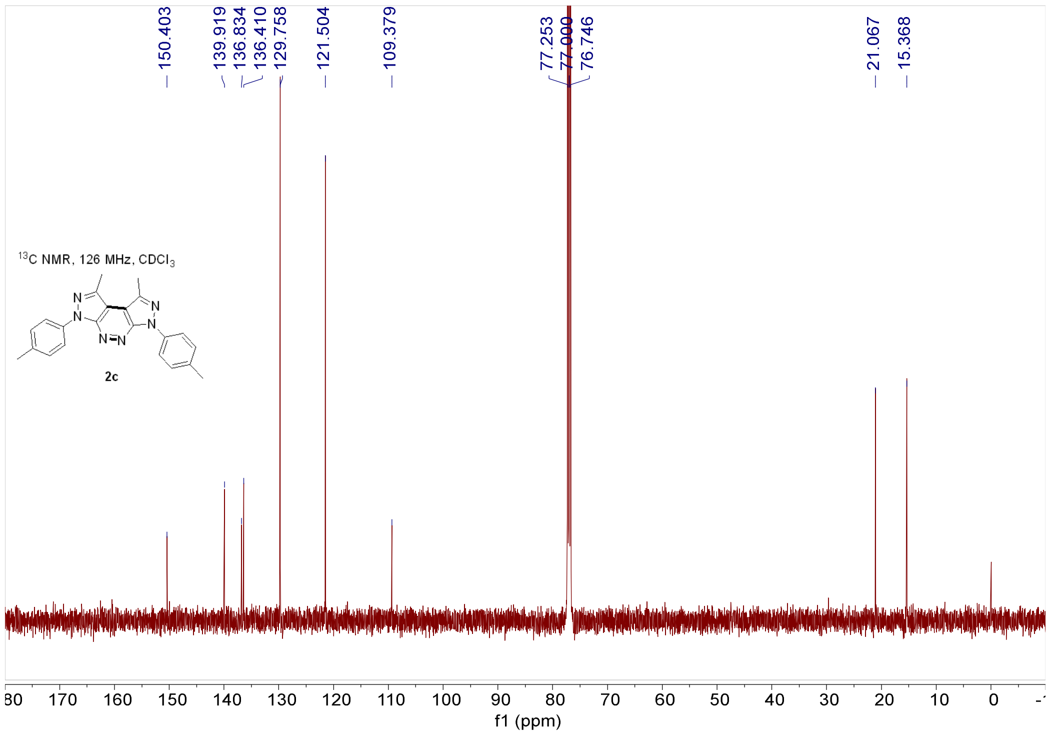
150.403
139.919
136.834
136.410
129.758
121.504
109.379
77.253
77.000
76.746
21.067
15.368
13C NMR, 126 MHz, CDCl3
2c
f1 (ppm)

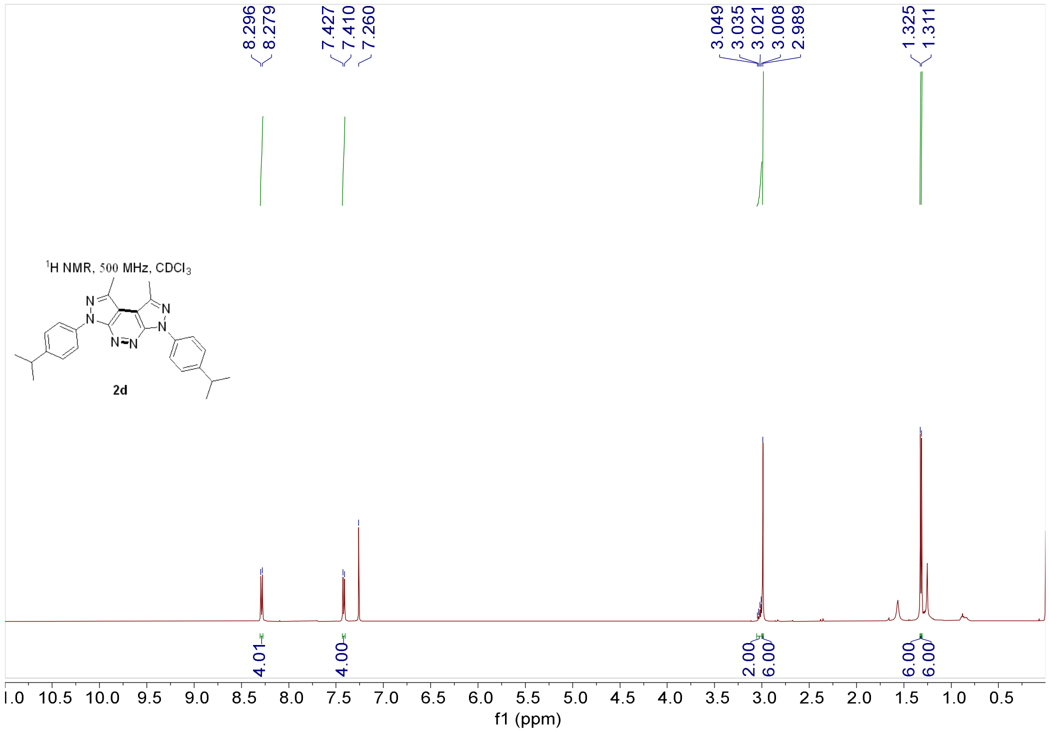
8.296
8.279
7.427
7.410
7.260
3.049
3.035
3.021
3.008
2.989
1.325
1.311
1H NMR, 500 MHz, CDCl3
2d
4.01
4.00
2.00
6.00
6.00
6.00
f1 (ppm)

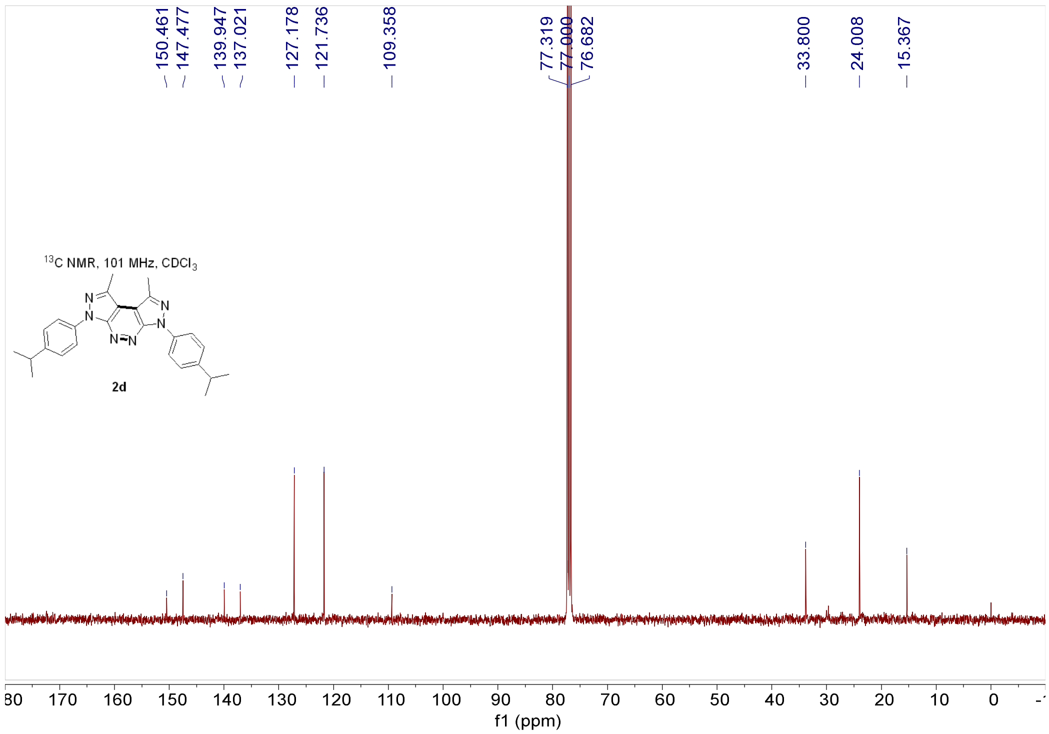

150.461
147.477
139.947
137.021
127.178
121.736
109.358
77.319
77.000
76.682
33.800
24.008
15.367
^{13}C NMR, 101 MHz, CDCl$_3$
2d
f1 (ppm)

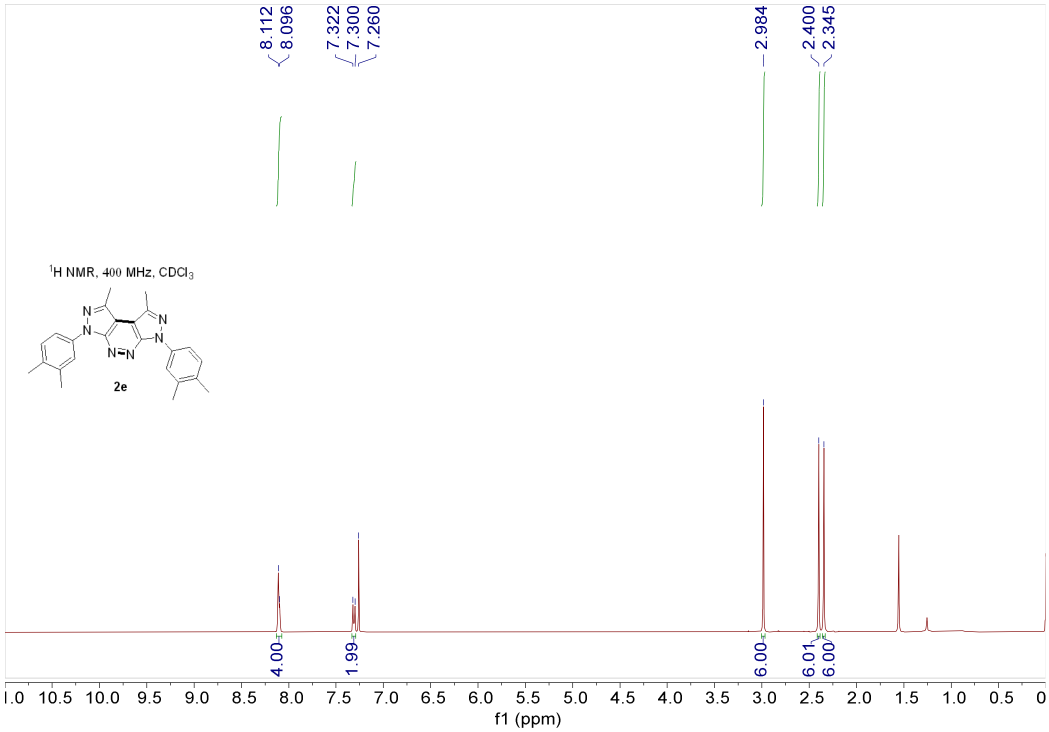

8.112
8.096
7.322
7.300
7.260
2.984
2.400
2.345
^{1}H NMR, 400 MHz, CDCl$_3$
2e
4.00
1.99
6.00
6.01
6.00
f1 (ppm)

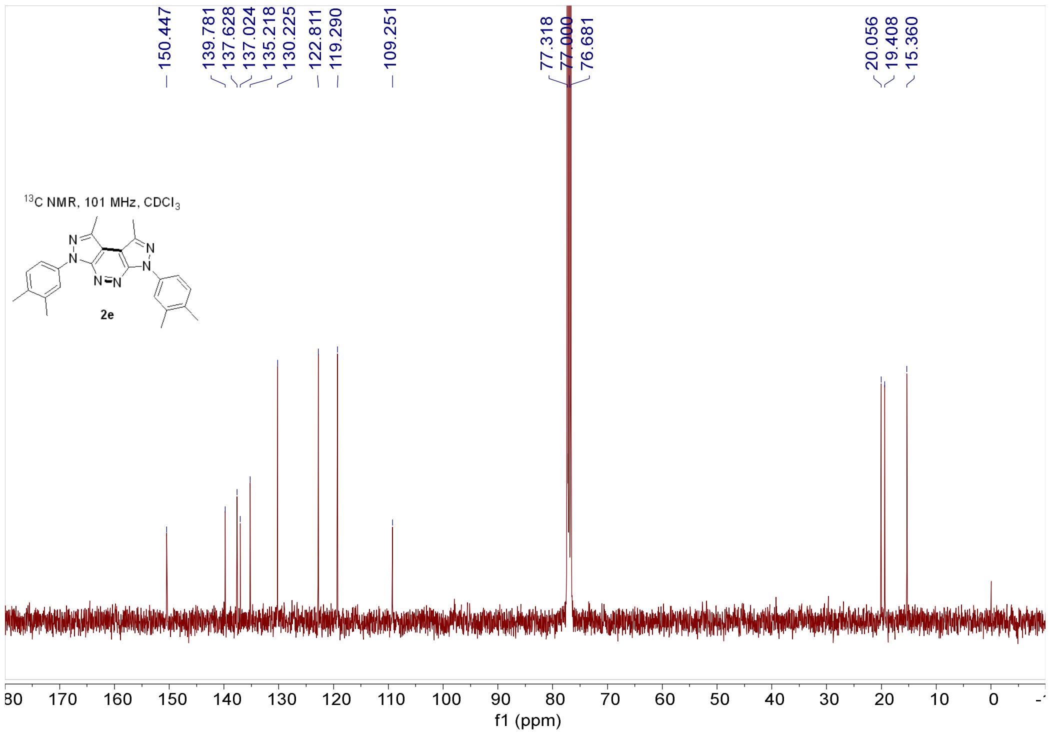
150.447
139.781
137.628
137.024
135.218
130.225
122.811
119.290
109.251
77.318
77.000
76.681
20.056
19.408
15.360
^{13}C NMR, 101 MHz, CDCl$_3$
2e
f1 (ppm)

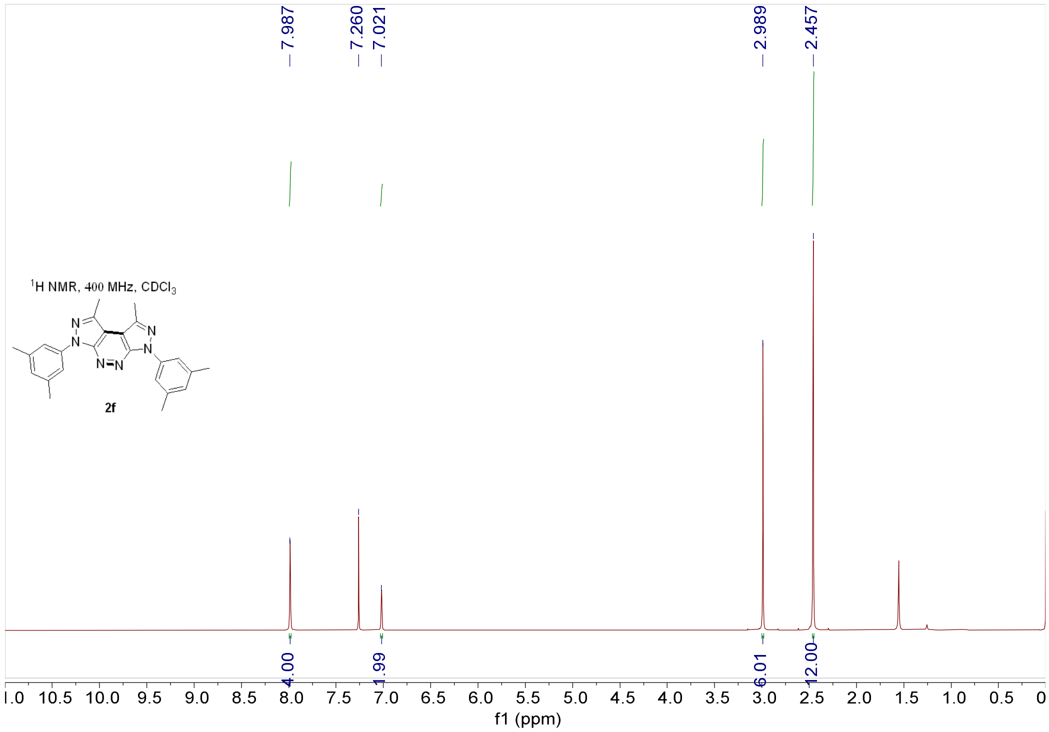
7.987
7.260
7.021
2.989
2.457
^{1}H NMR, 400 MHz, CDCl$_3$
2f
4.00
1.99
6.01
12.00
f1 (ppm)

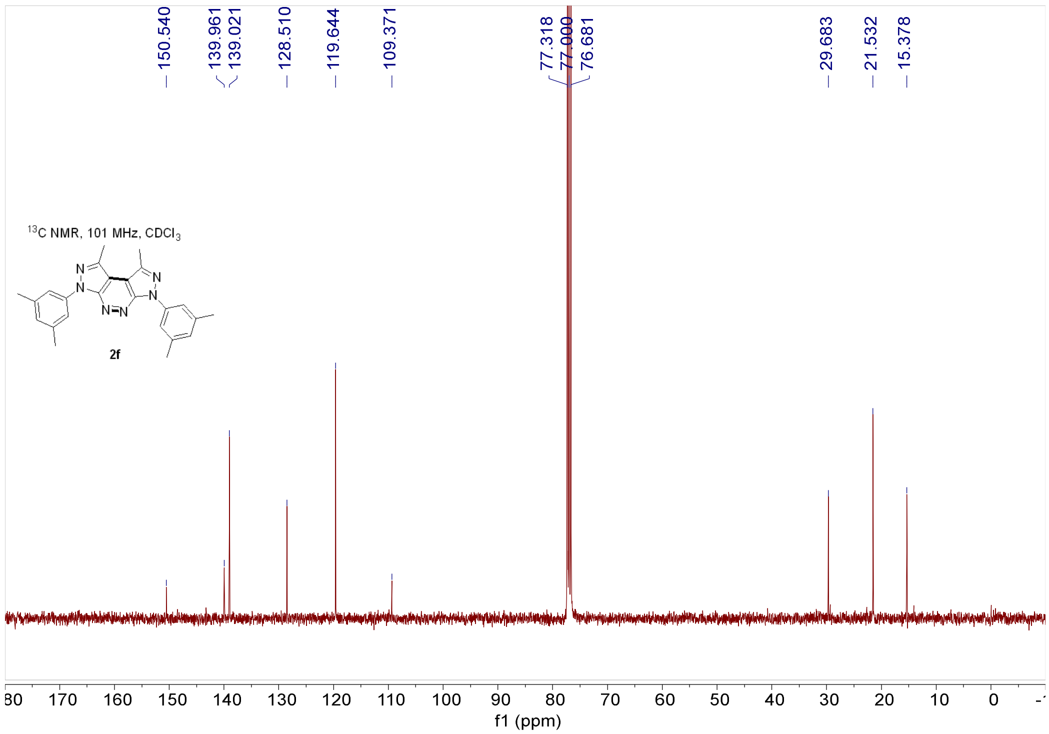
150.540
139.961
139.021
128.510
119.644
109.371
77.318
77.000
76.681
29.683
21.532
15.378
13C NMR, 101 MHz, CDCl3
2f
f1 (ppm)

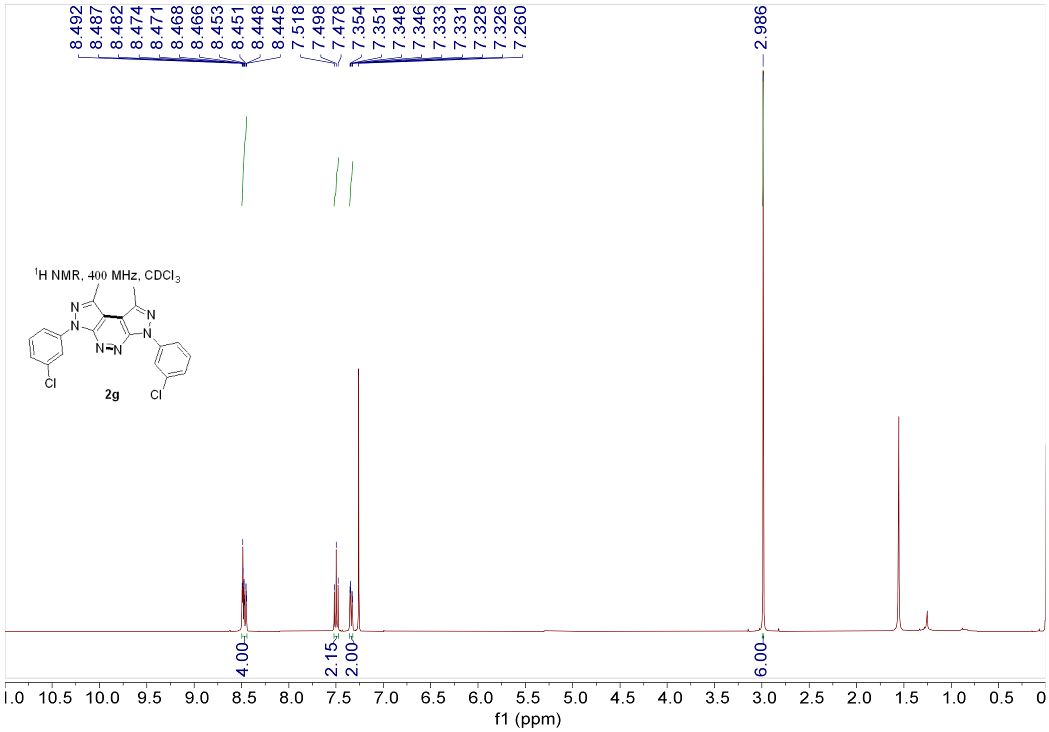
8.492
8.487
8.482
8.474
8.471
8.468
8.466
8.453
8.451
8.448
8.445
7.518
7.498
7.478
7.354
7.351
7.348
7.346
7.333
7.331
7.328
7.326
7.260
2.986
1H NMR, 400 MHz, CDCl3
2g
Cl
Cl
4.00
2.15
2.00
6.00
f1 (ppm)

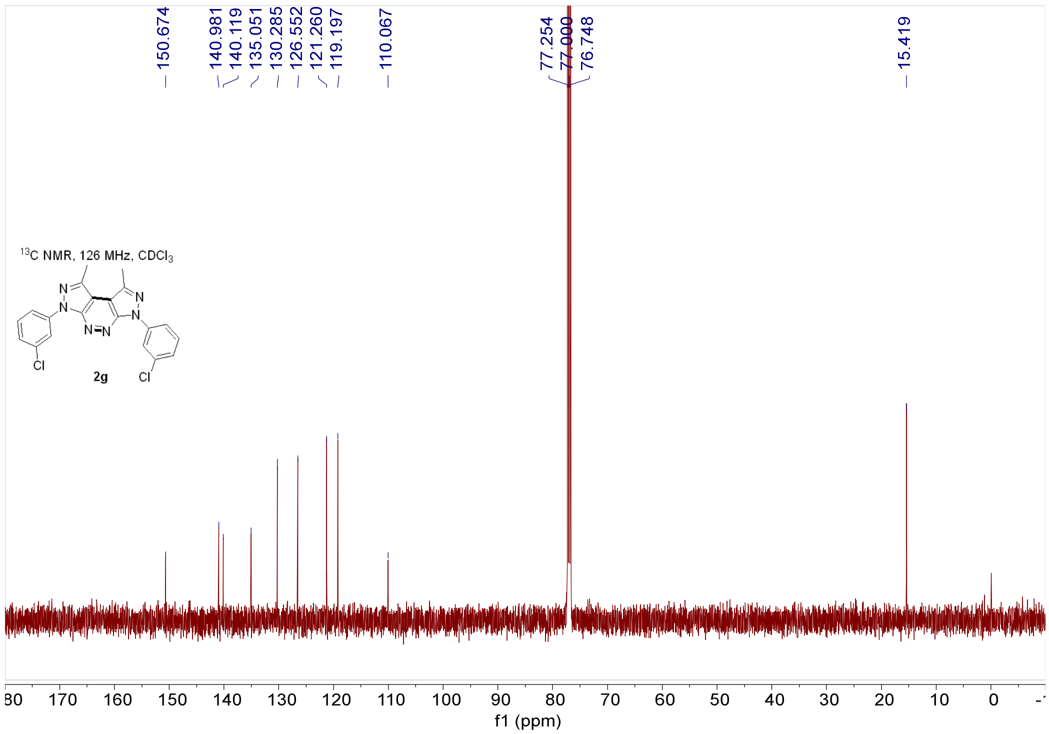

150.674
140.981
140.119
135.051
130.285
126.552
121.260
119.197
110.067
77.254
77.000
76.748
15.419
13C NMR, 126 MHz, CDCl3
2g
Cl
Cl
f1 (ppm)

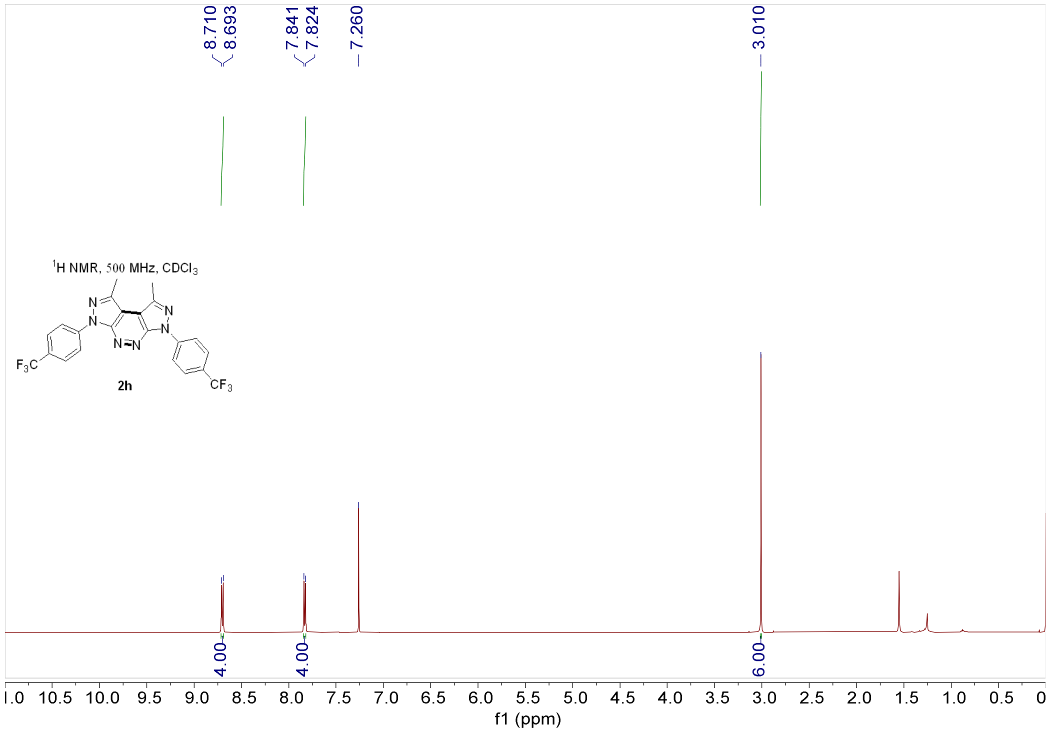

8.710
8.693
7.841
7.824
7.260
3.010
1H NMR, 500 MHz, CDCl3
2h
F3C
CF3
4.00
4.00
6.00
f1 (ppm)

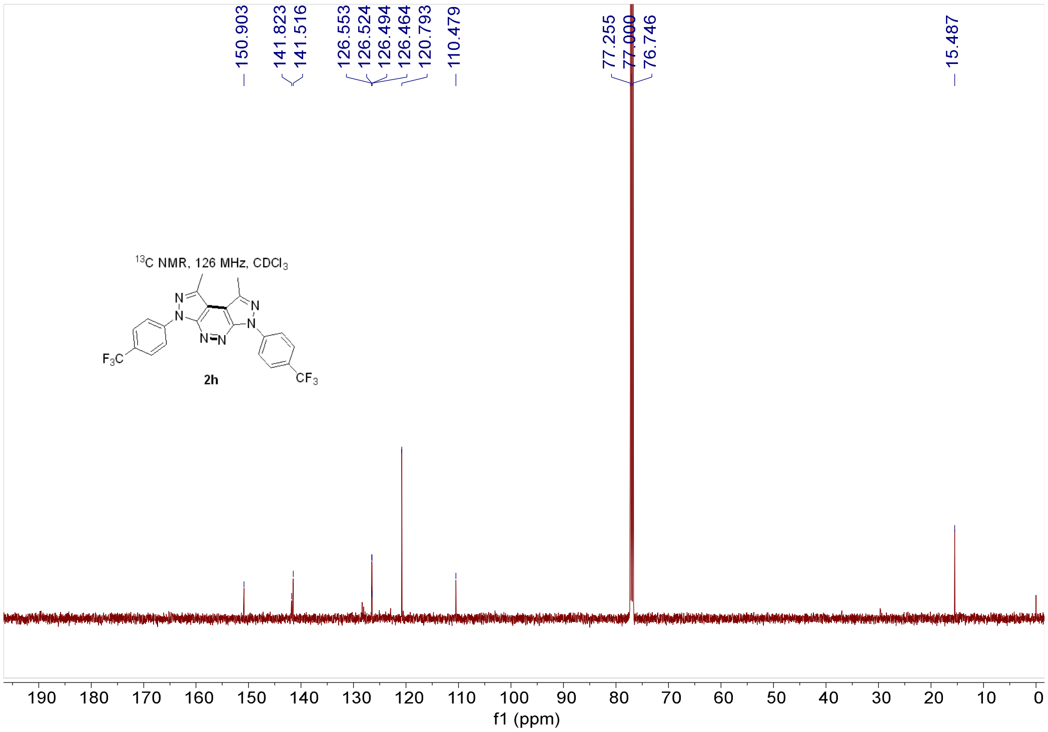
150.903
141.823
141.516
126.553
126.524
126.494
126.464
120.793
110.479
77.255
77.000
76.746
15.487
13C NMR, 126 MHz, CDCl3
2h
190 180 170 160 150 140 130 120 110 100 90 80 70 60 50 40 30 20 10 0
f1 (ppm)

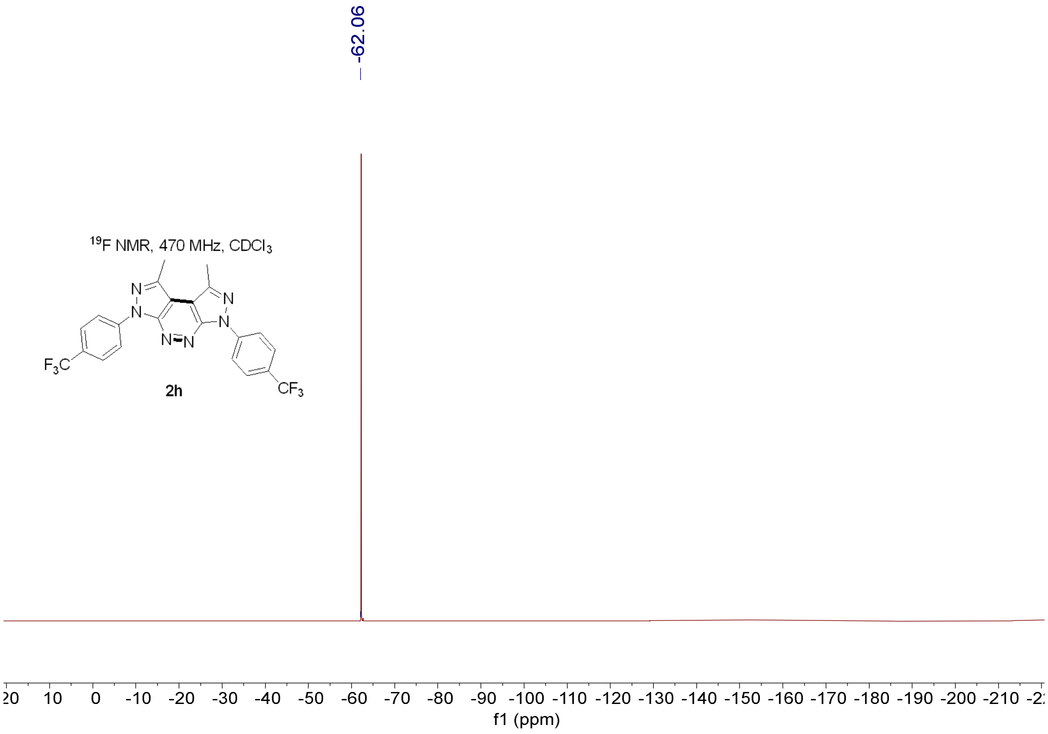
-62.06
19F NMR, 470 MHz, CDCl3
2h
20 10 0 -10 -20 -30 -40 -50 -60 -70 -80 -90 -100 -110 -120 -130 -140 -150 -160 -170 -180 -190 -200 -210
f1 (ppm)

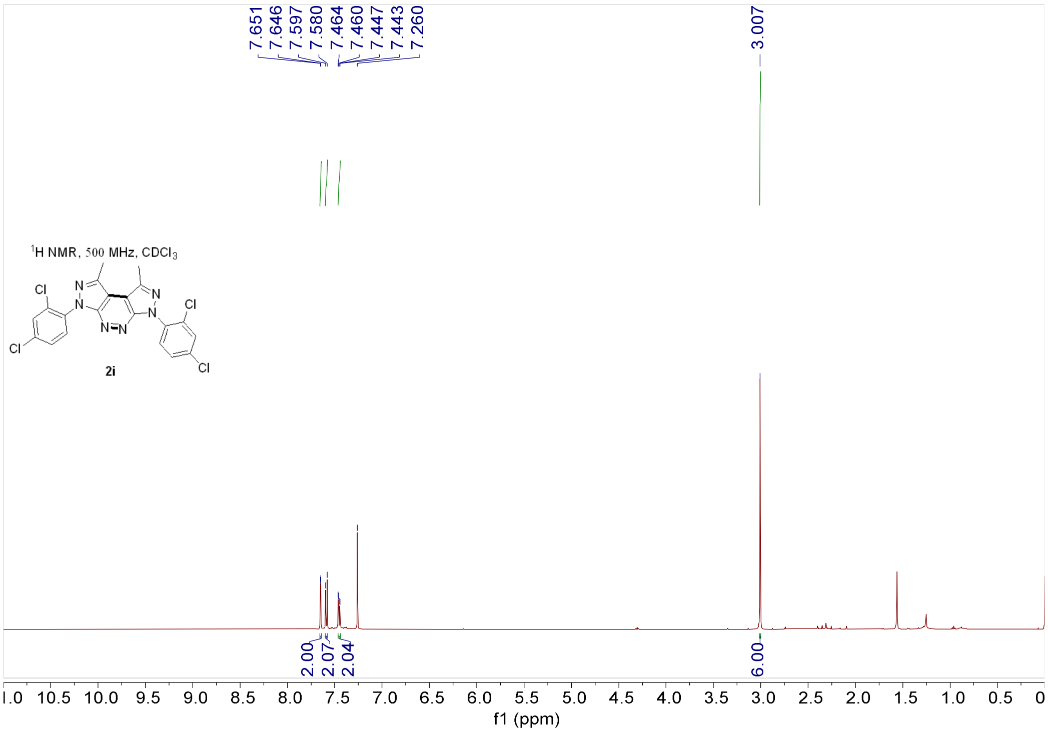
7.651
7.646
7.597
7.580
7.464
7.460
7.447
7.443
7.260
3.007
1H NMR, 500 MHz, CDCl3
2i
2.00
2.07
2.04
6.00
f1 (ppm)

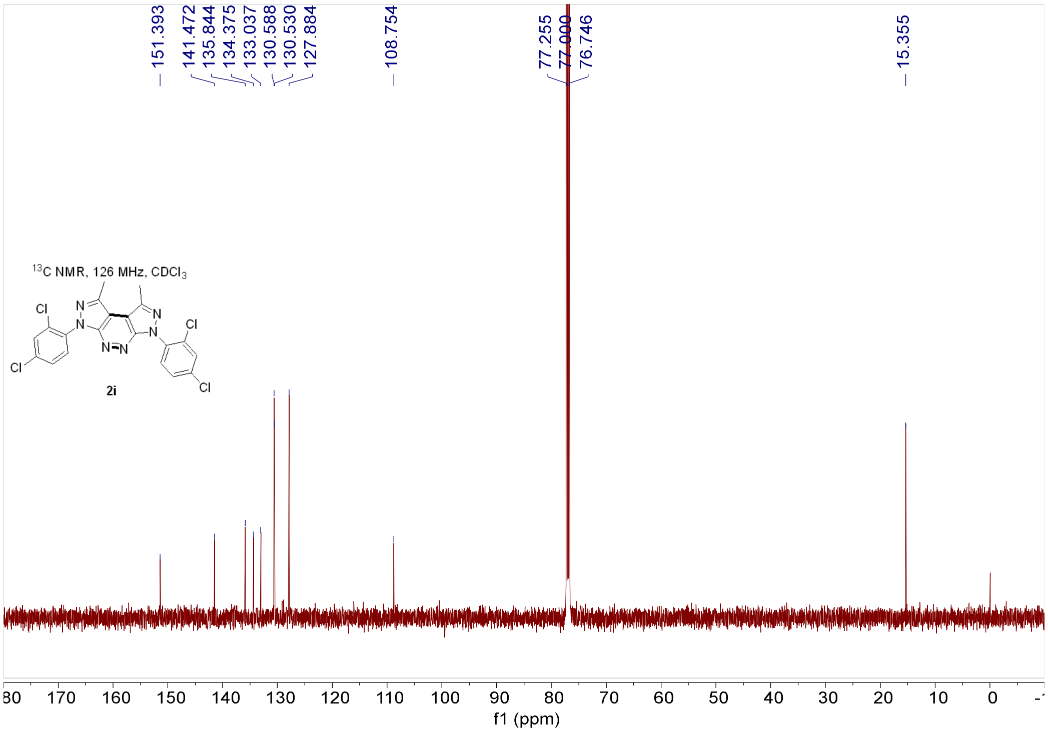
151.393
141.472
135.844
134.375
133.037
130.588
130.530
127.884
108.754
77.255
77.000
76.746
15.355
13C NMR, 126 MHz, CDCl3
2i
f1 (ppm)

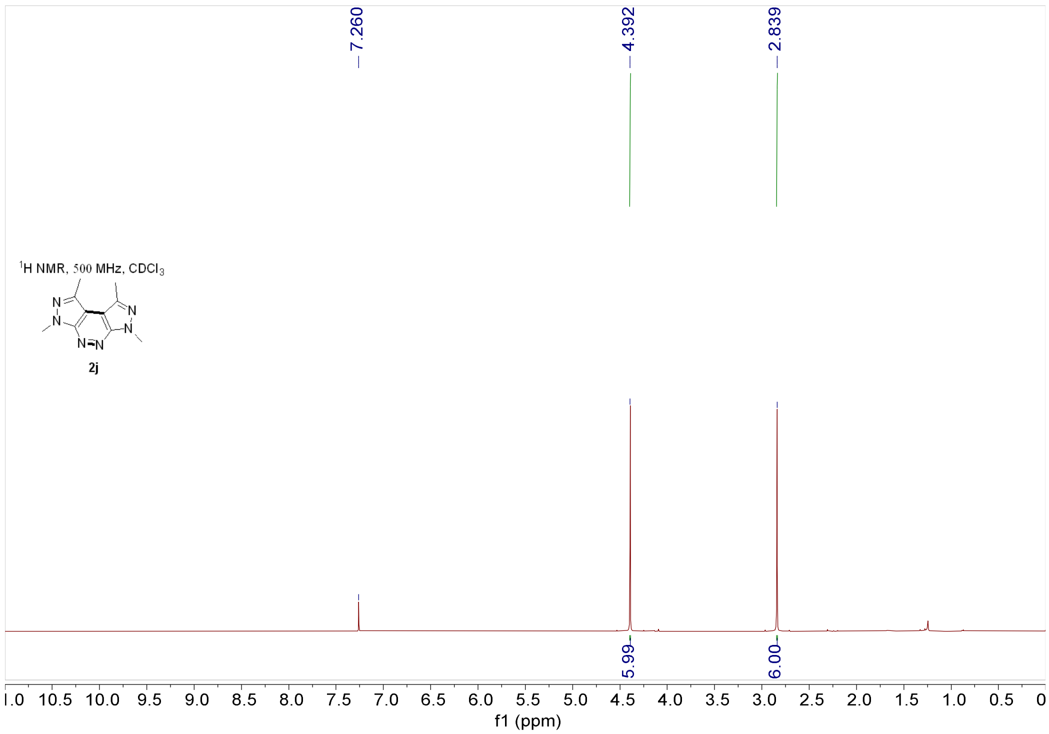

7.260
4.392
2.839
^{1}H NMR, 500 MHz, CDCl$_3$
2j
5.99
6.00
f1 (ppm)

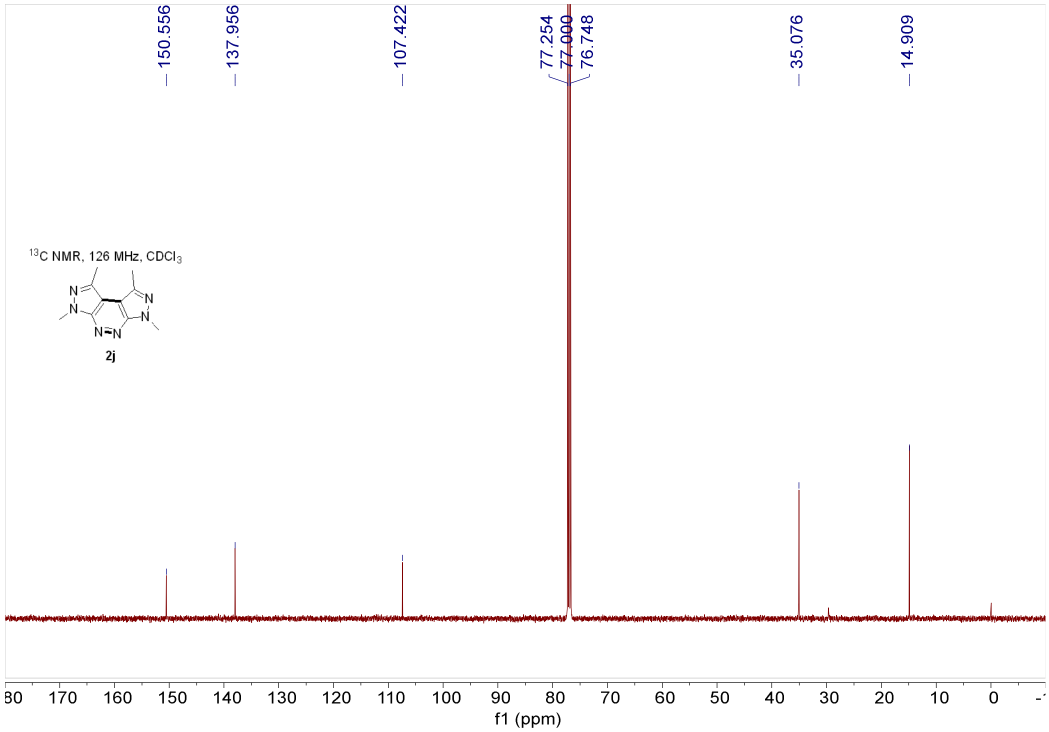

150.556
137.956
107.422
77.254
77.000
76.748
35.076
14.909
^{13}C NMR, 126 MHz, CDCl$_3$
2j
f1 (ppm)

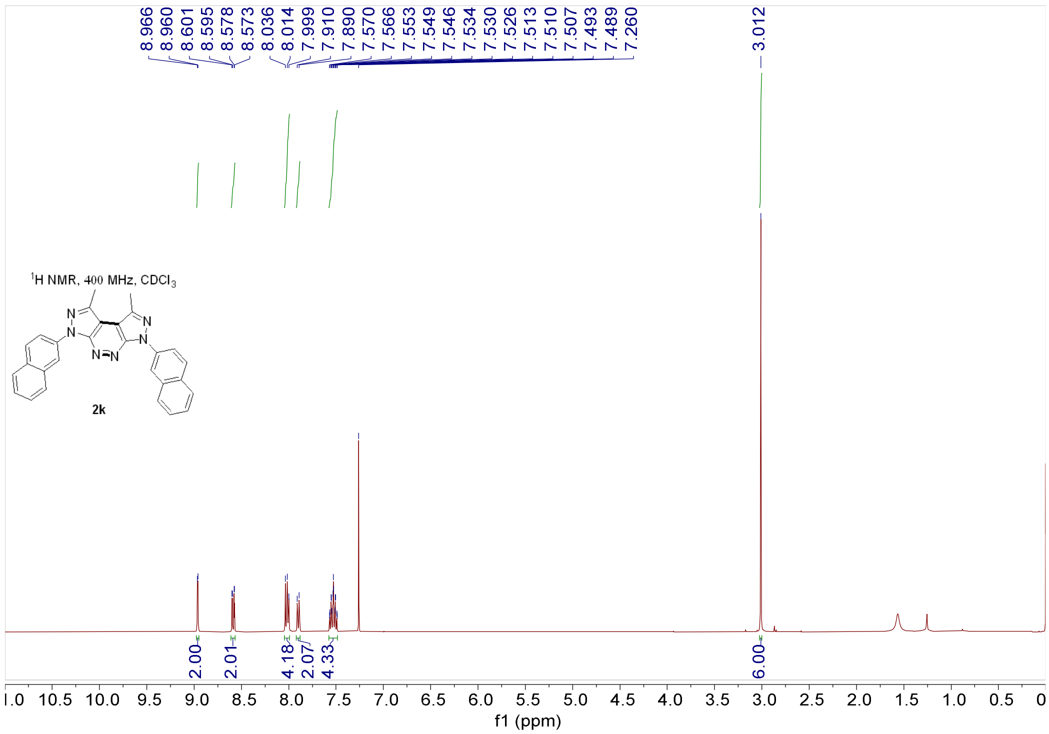
8.966
8.960
8.601
8.595
8.578
8.573
8.036
8.014
7.999
7.910
7.890
7.570
7.566
7.553
7.549
7.546
7.534
7.530
7.526
7.513
7.510
7.507
7.493
7.489
7.260
3.012
^{1}H NMR, 400 MHz, $CDCl_3$
2k
2.00
2.01
4.18
2.07
4.33
6.00
f1 (ppm)

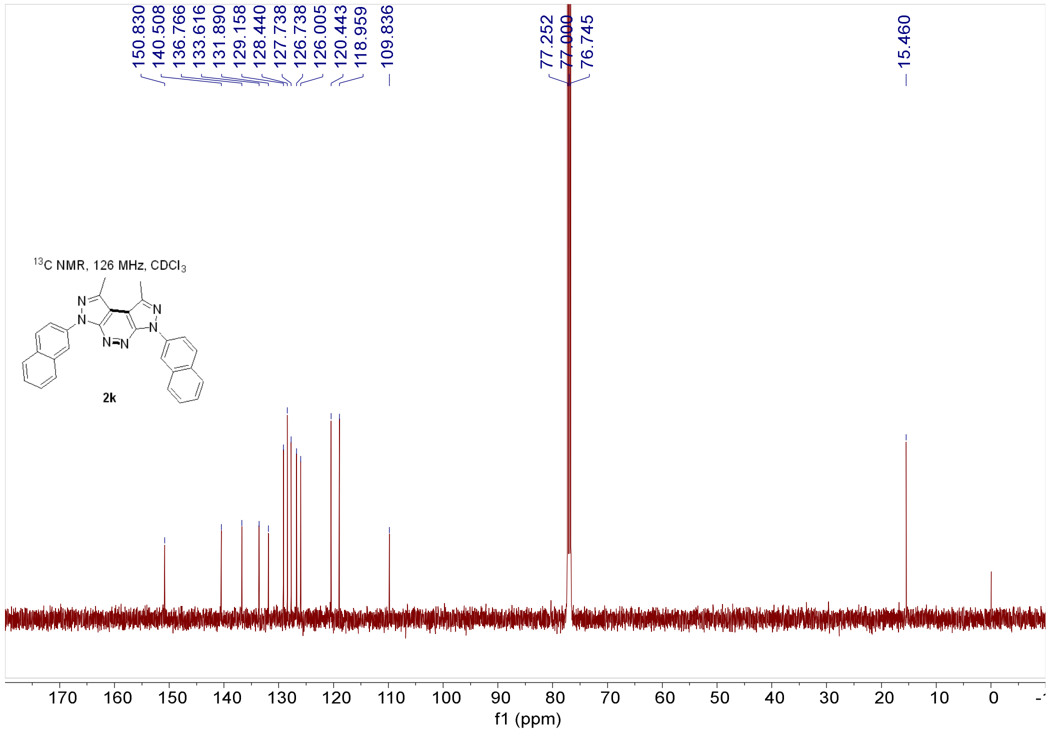
150.830
140.508
136.766
133.616
131.890
129.158
128.440
127.738
126.738
126.005
120.443
118.959
109.836
77.252
77.000
76.745
15.460
^{13}C NMR, 126 MHz, $CDCl_3$
2k
f1 (ppm)

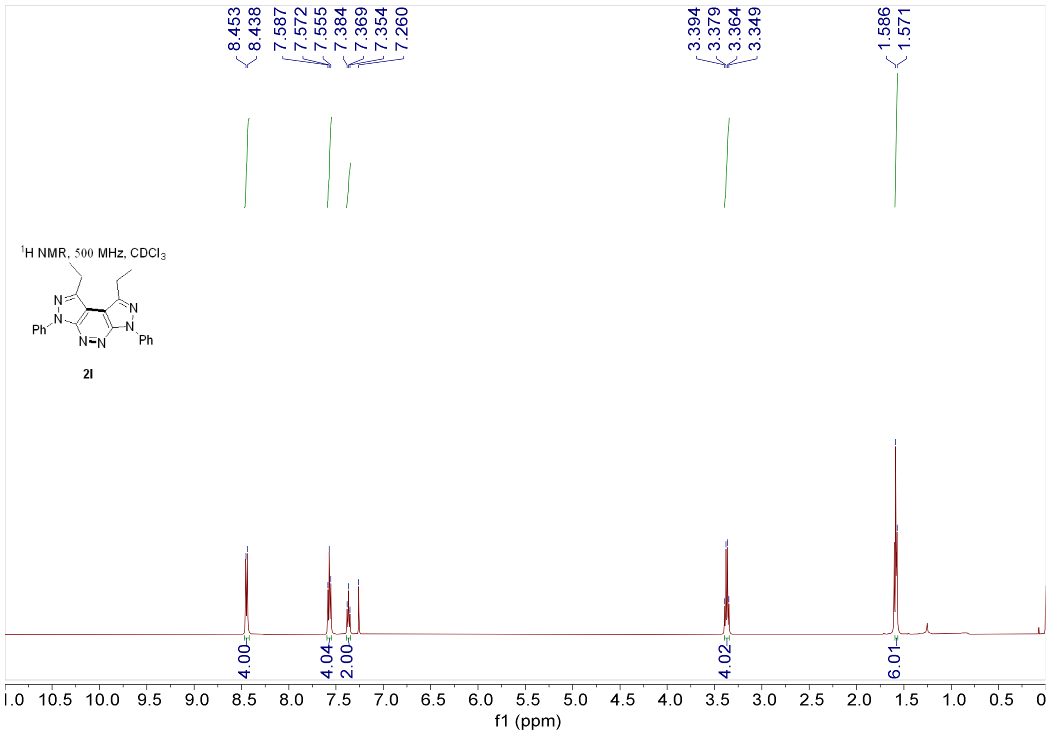
8.453
8.438
7.587
7.572
7.555
7.384
7.369
7.354
7.260
3.394
3.379
3.364
3.349
1.586
1.571
1H NMR, 500 MHz, CDCl3
N
N
Ph
N
N
Ph
N=N
2l
4.00
4.04
2.00
4.02
6.01
f1 (ppm)

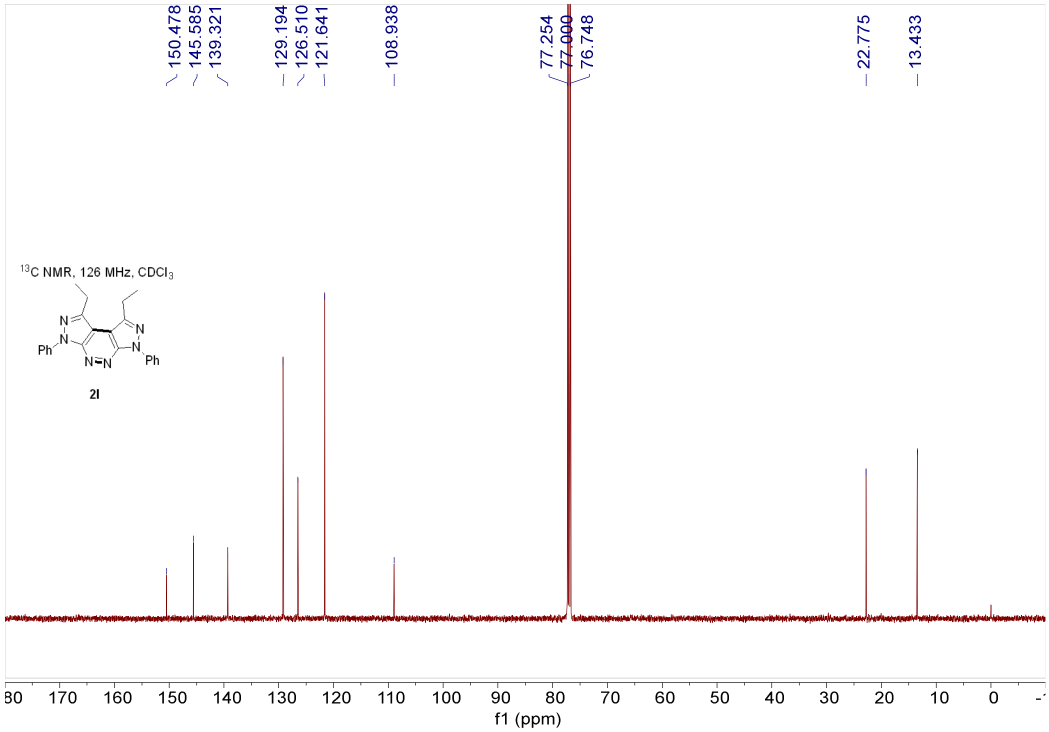
150.478
145.585
139.321
129.194
126.510
121.641
108.938
77.254
77.000
76.748
22.775
13.433
13C NMR, 126 MHz, CDCl3
N
N
Ph
N
N
Ph
N=N
2l
f1 (ppm)

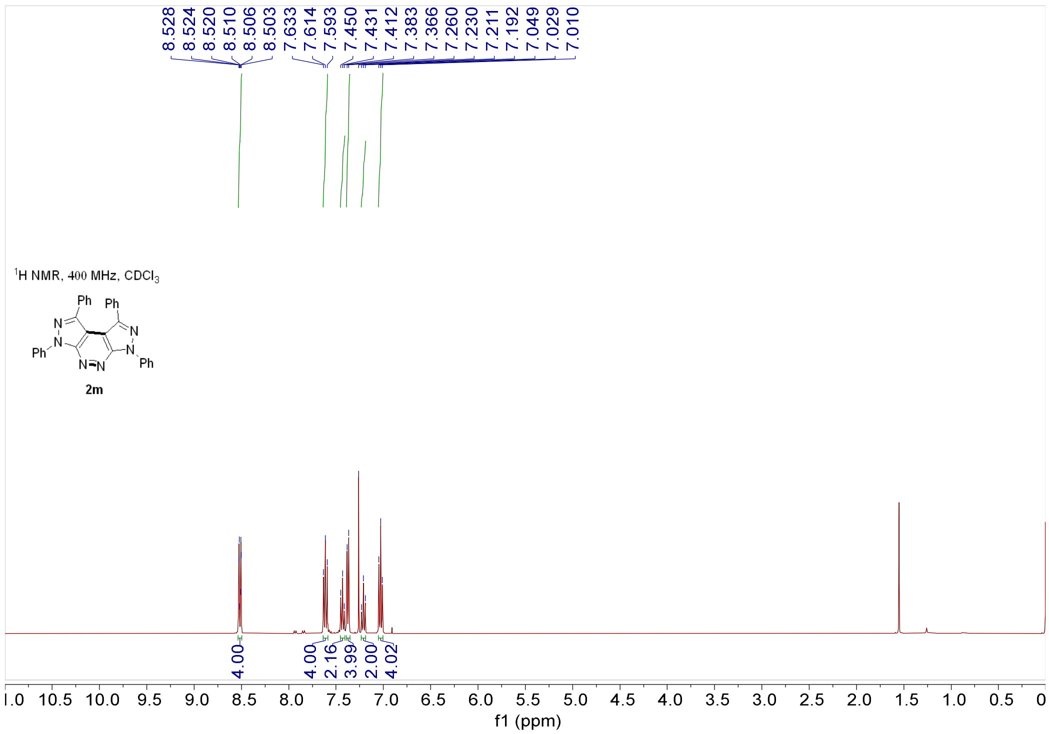
8.528
8.524
8.520
8.510
8.506
8.503
7.633
7.614
7.593
7.450
7.431
7.412
7.383
7.366
7.260
7.230
7.211
7.192
7.049
7.029
7.010
1H NMR, 400 MHz, CDCl3
Ph
Ph
N
N
N
N
Ph
N
N
N
N
Ph
2m
4.00
4.00
2.16
3.99
2.00
4.02
f1 (ppm)

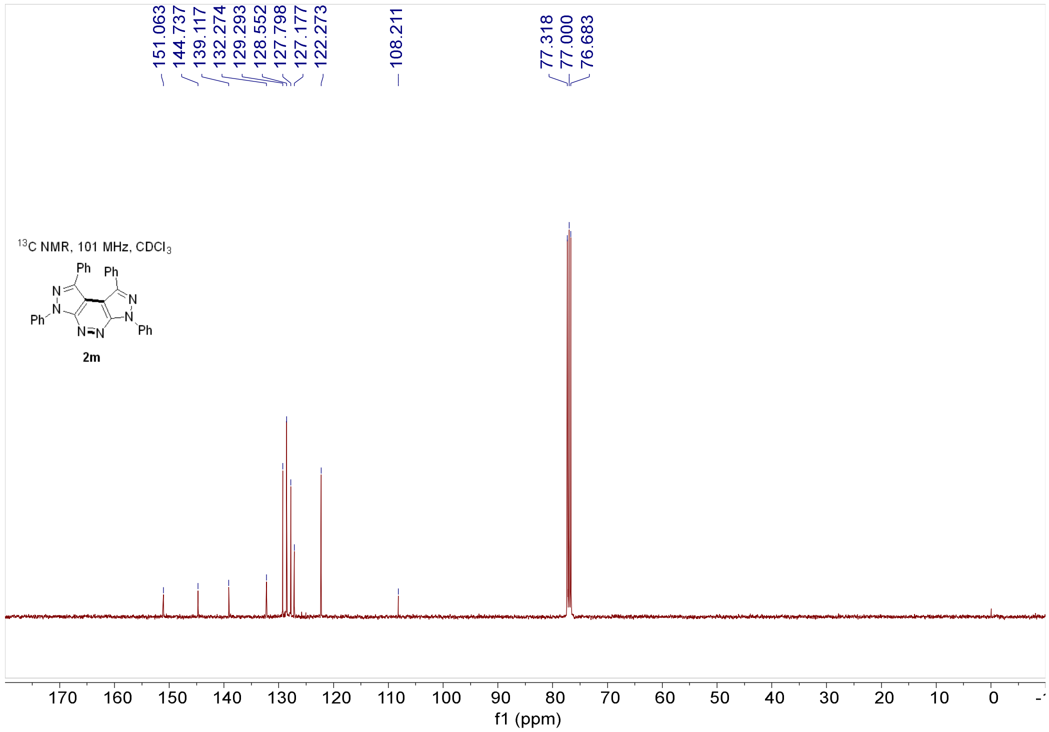
151.063
144.737
139.117
132.274
129.293
128.552
127.798
127.177
122.273
108.211
77.318
77.000
76.683
13C NMR, 101 MHz, CDCl3
Ph
Ph
N
N
N
N
Ph
N
N
N
N
Ph
2m
f1 (ppm)

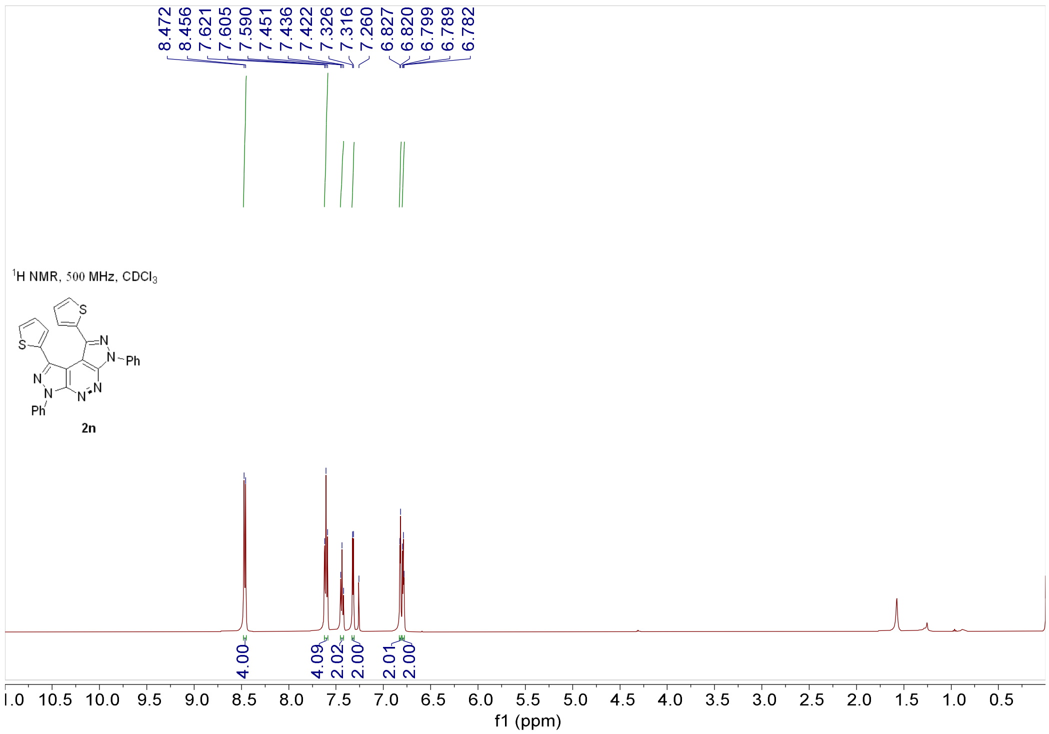
8.472
8.456
7.621
7.605
7.590
7.451
7.436
7.422
7.326
7.316
7.260
6.827
6.820
6.799
6.789
6.782
1H NMR, 500 MHz, CDCl3
S
N
Ph
2n
4.00
4.09
2.02
2.00
2.01
2.00
f1 (ppm)

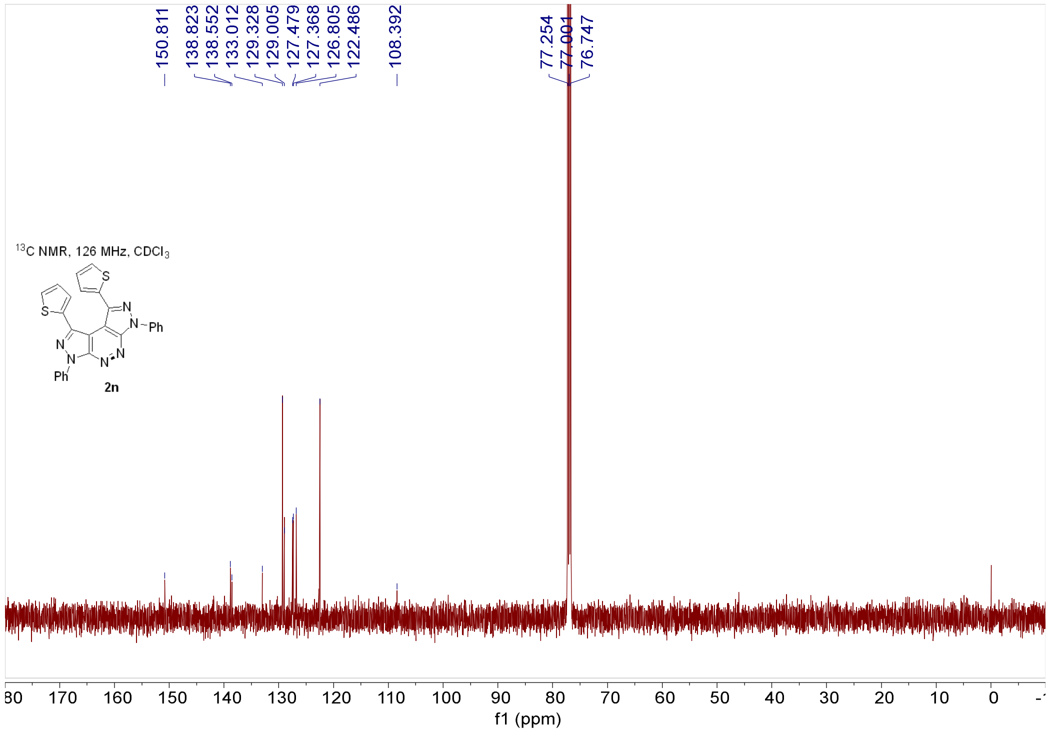
150.811
138.823
138.552
133.012
129.328
129.005
127.479
127.368
126.805
122.486
108.392
77.254
77.001
76.747
13C NMR, 126 MHz, CDCl3
2n
f1 (ppm)

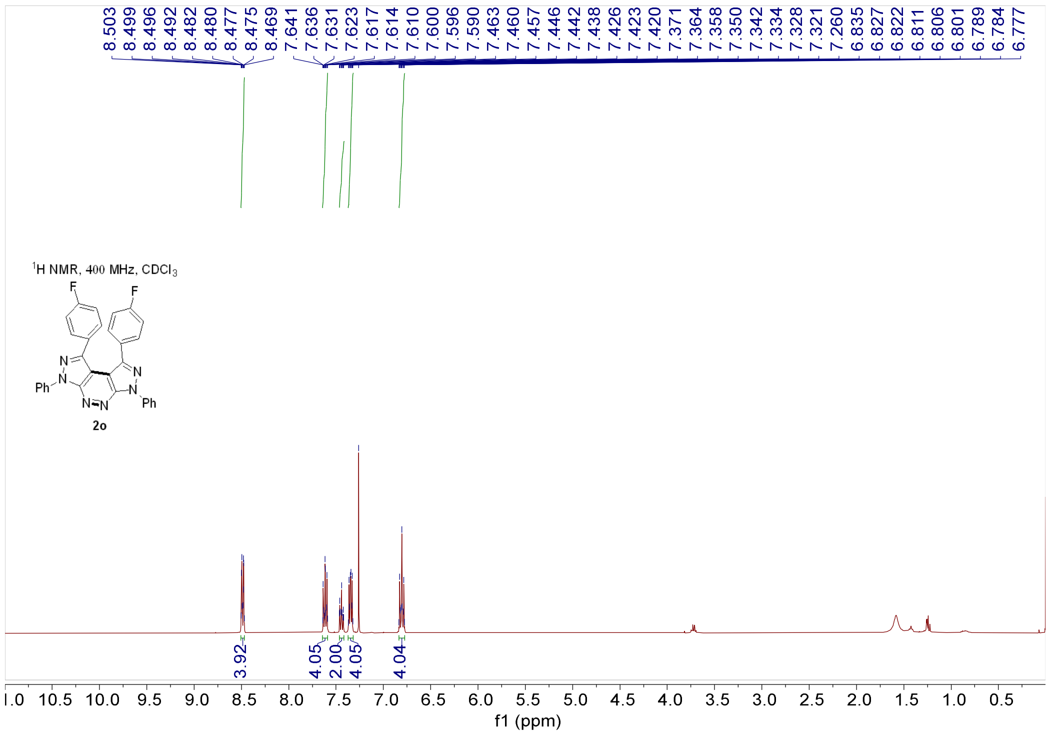

8.503
8.499
8.496
8.492
8.482
8.480
8.477
8.475
8.469
7.641
7.636
7.631
7.623
7.617
7.614
7.610
7.600
7.596
7.590
7.463
7.460
7.457
7.446
7.442
7.438
7.426
7.423
7.420
7.371
7.364
7.358
7.350
7.342
7.334
7.328
7.321
7.260
6.835
6.827
6.822
6.811
6.806
6.801
6.789
6.784
6.777
1H NMR, 400 MHz, CDCl3
2o
3.92
4.05
2.00
4.05
4.04
f1 (ppm)

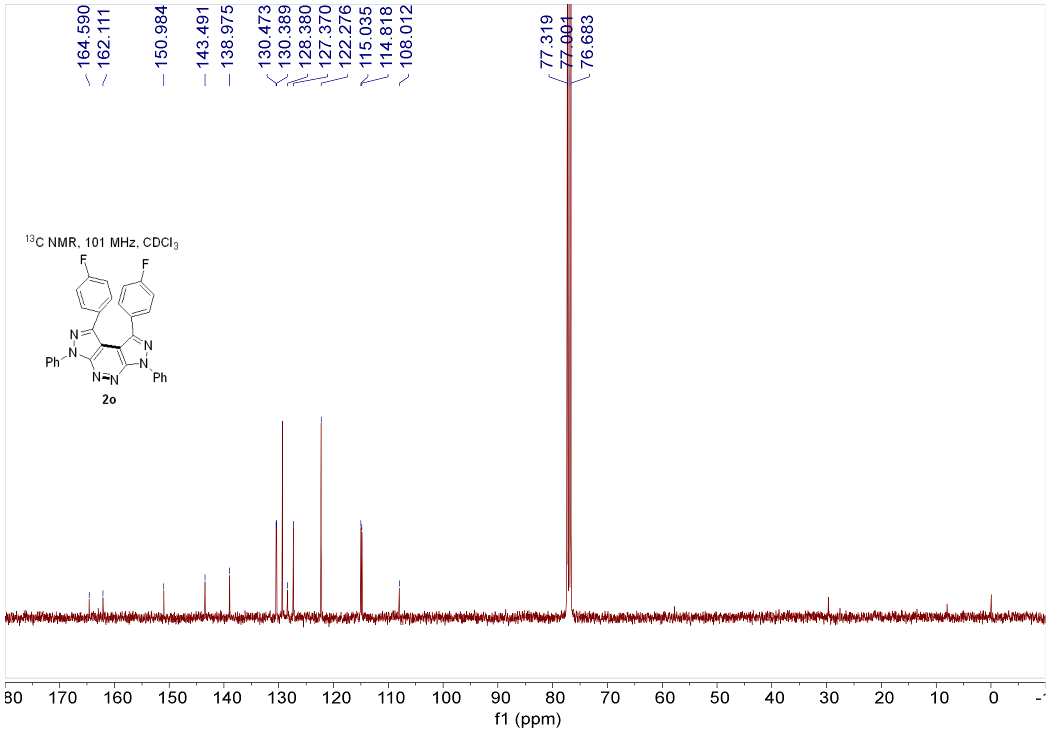

164.590
162.111
150.984
143.491
138.975
130.473
130.389
128.380
127.370
122.276
115.035
114.818
108.012
77.319
77.001
76.683
13C NMR, 101 MHz, CDCl3
2o
f1 (ppm)

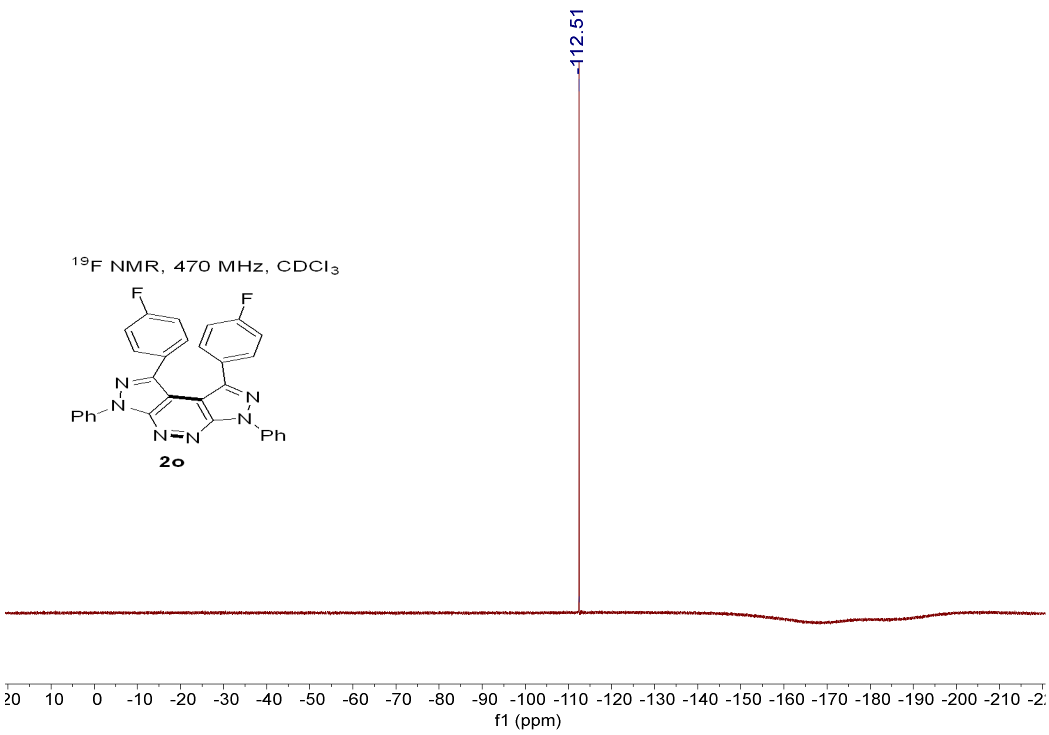

-112.51
¹⁹F NMR, 470 MHz, CDCl₃
F
F
N
N
N
N
Ph
Ph
N
N
2o
20
10
0
-10
-20
-30
-40
-50
-60
-70
-80
-90
-100
-110
-120
-130
-140
-150
-160
-170
-180
-190
-200
-210
f1 (ppm)

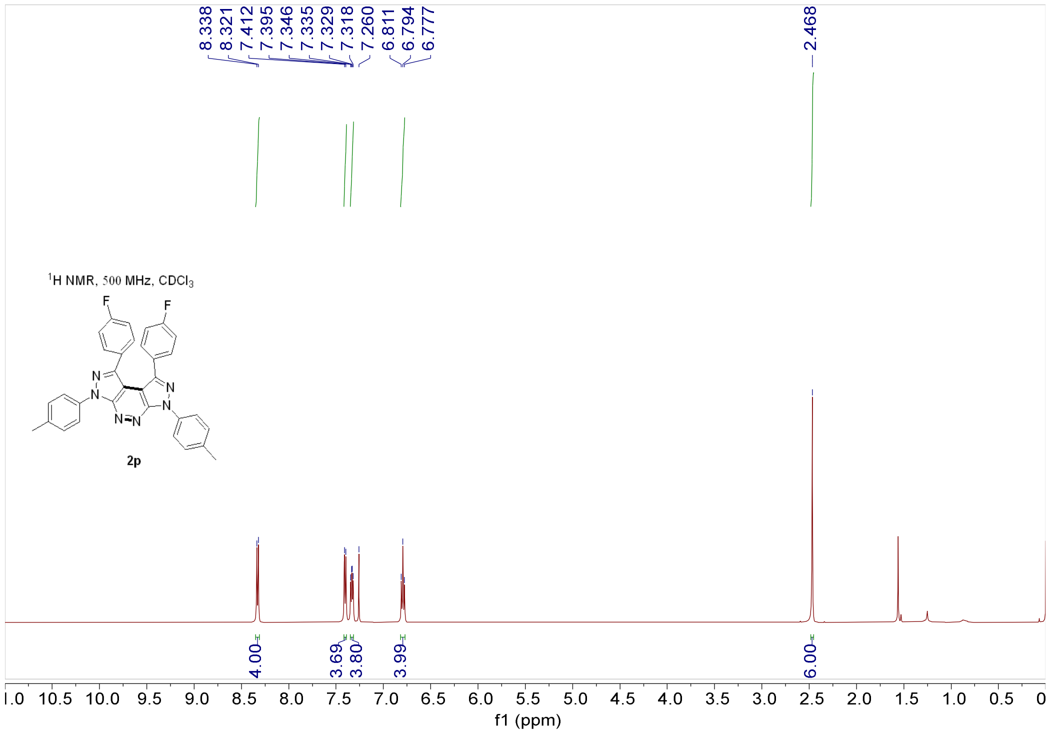

8.338
8.321
7.412
7.395
7.346
7.335
7.329
7.318
7.260
6.811
6.794
6.777
2.468
¹H NMR, 500 MHz, CDCl₃
F
F
N
N
N
N
N
N
2p
4.00
3.69
3.80
3.99
6.00
10.5
10.0
9.5
9.0
8.5
8.0
7.5
7.0
6.5
6.0
5.5
5.0
4.5
4.0
3.5
3.0
2.5
2.0
1.5
1.0
0.5
0
f1 (ppm)

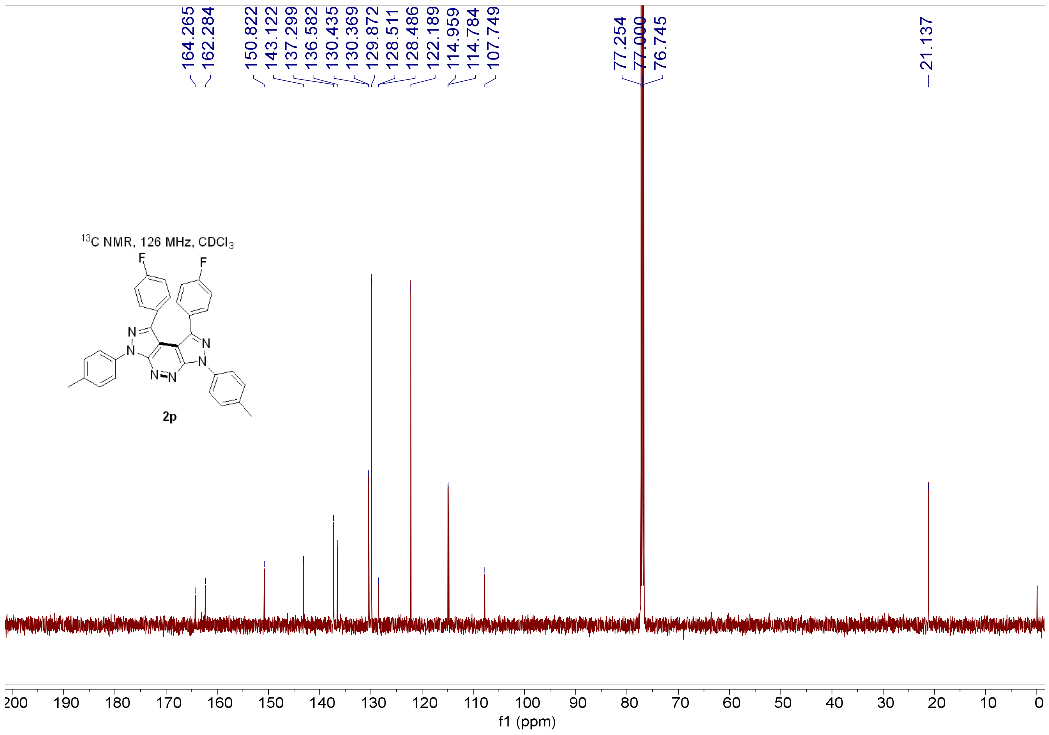
164.265
162.284
150.822
143.122
137.299
136.582
130.435
130.369
129.872
128.511
128.486
122.189
114.959
114.784
107.749
77.254
77.000
76.745
21.137
^{13}C NMR, 126 MHz, $CDCl_3$
2p
f1 (ppm)

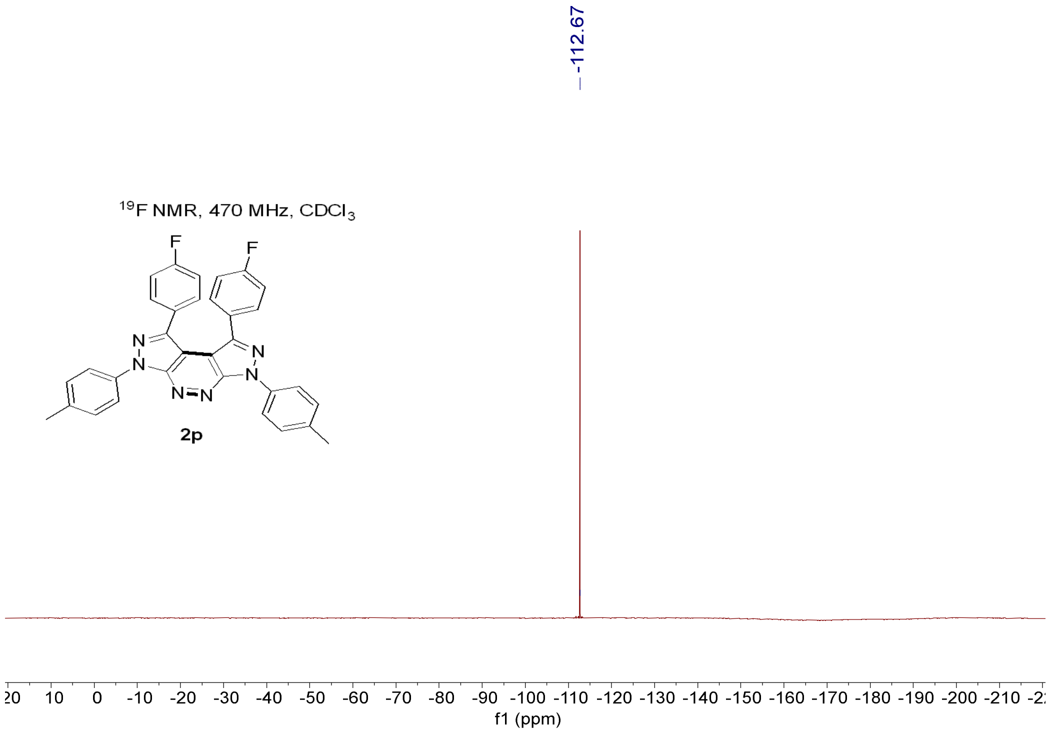
-112.67
^{19}F NMR, 470 MHz, $CDCl_3$
2p
f1 (ppm)

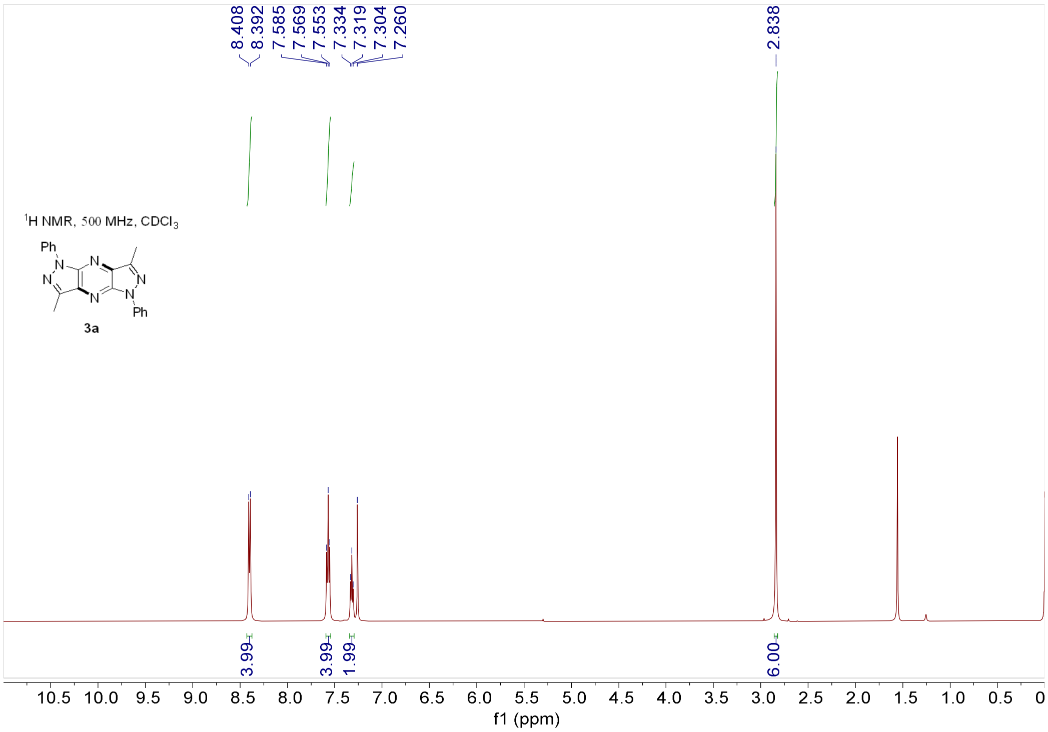
8.408
8.392
7.585
7.569
7.553
7.334
7.319
7.304
7.260
2.838
^{1}H NMR, 500 MHz, CDCl$_3$
3a
3.99
3.99
1.99
6.00
10.5 10.0 9.5 9.0 8.5 8.0 7.5 7.0 6.5 6.0 5.5 5.0 4.5 4.0 3.5 3.0 2.5 2.0 1.5 1.0 0.5 0
f1 (ppm)

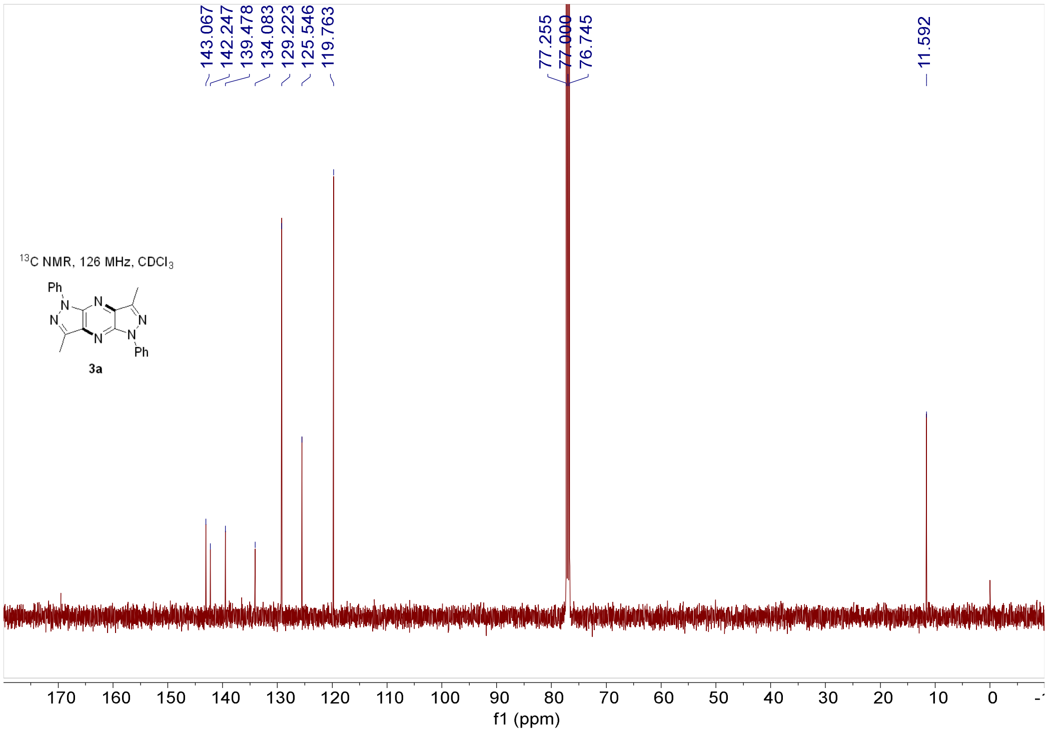
143.067
142.247
139.478
134.083
129.223
125.546
119.763
77.255
77.000
76.745
11.592
^{13}C NMR, 126 MHz, CDCl$_3$
3a
170 160 150 140 130 120 110 100 90 80 70 60 50 40 30 20 10 0
f1 (ppm)

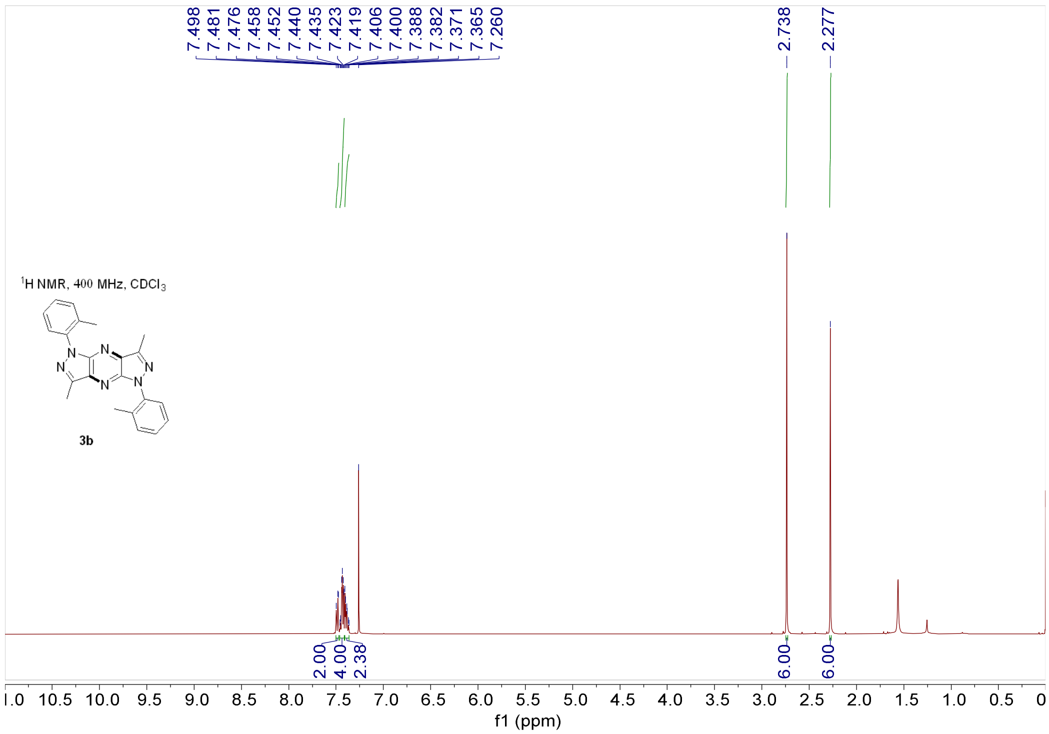
7.498
7.481
7.476
7.458
7.452
7.440
7.435
7.423
7.419
7.406
7.400
7.388
7.382
7.371
7.365
7.260
2.738
2.277
1H NMR, 400 MHz, CDCl3
3b
2.00
4.00
2.38
6.00
6.00
f1 (ppm)

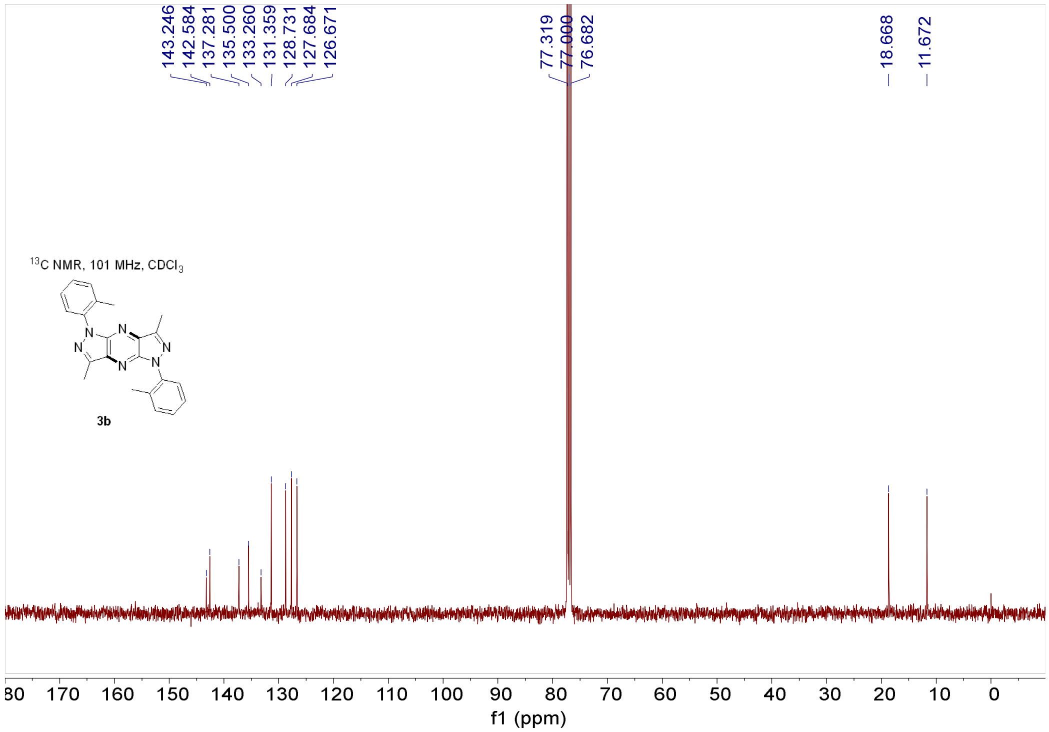
143.246
142.584
137.281
135.500
133.260
131.359
128.731
127.684
126.671
77.319
77.000
76.682
18.668
11.672
13C NMR, 101 MHz, CDCl3
3b
f1 (ppm)

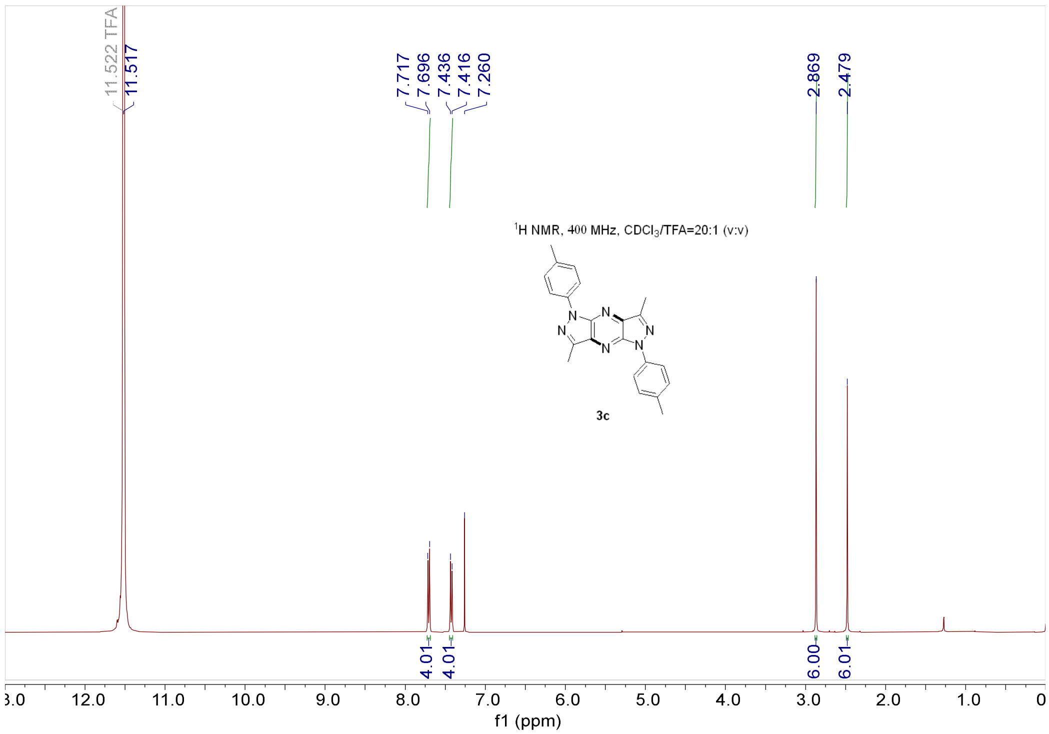
11.522 TFA
11.517
7.717
7.696
7.436
7.416
7.260
2.869
2.479
1H NMR, 400 MHz, CDCl3/TFA=20:1 (v:v)
3c
4.01
4.01
6.00
6.01
f1 (ppm)

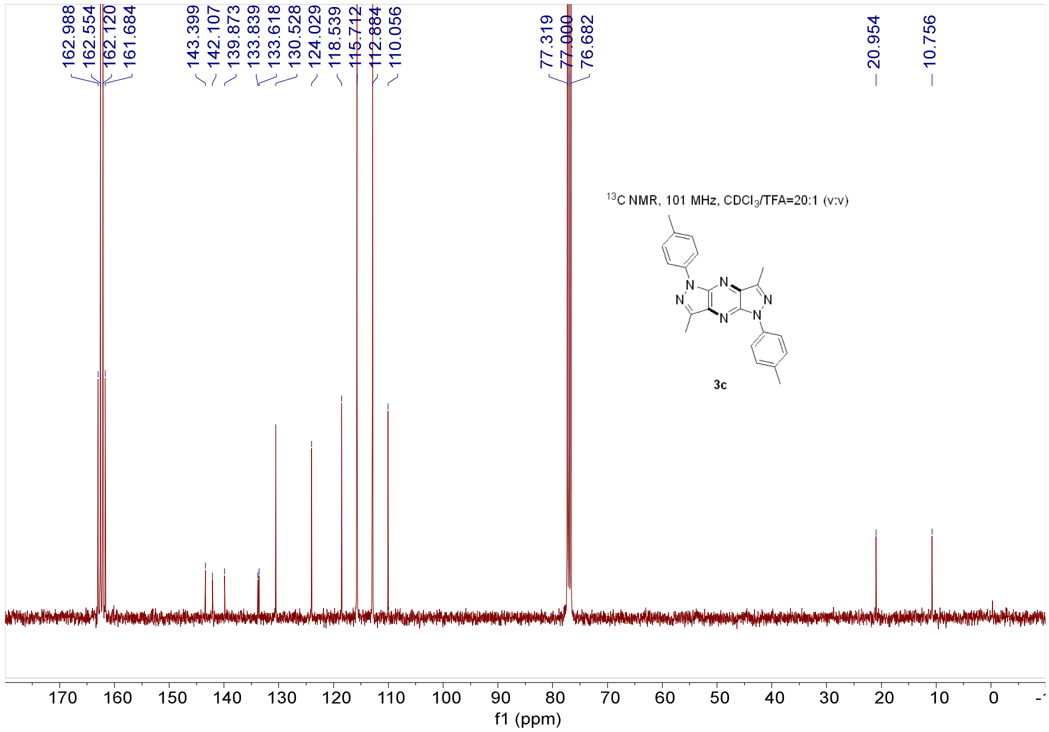
162.988
162.554
162.120
161.684
143.399
142.107
139.873
133.839
133.618
130.528
124.029
118.539
115.712
112.884
110.056
77.319
77.000
76.682
20.954
10.756
13C NMR, 101 MHz, CDCl3/TFA=20:1 (v:v)
3c
f1 (ppm)

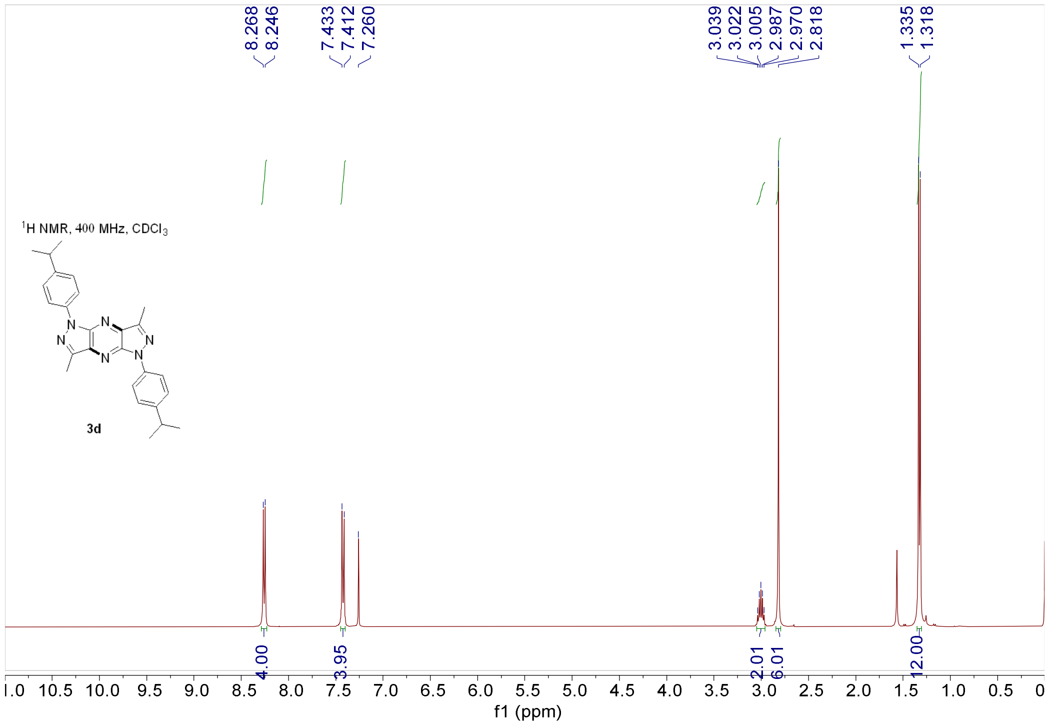
8.268
8.246
7.433
7.412
7.260
3.039
3.022
3.005
2.987
2.970
2.818
1.335
1.318
1H NMR, 400 MHz, CDCl3
3d
4.00
3.95
2.01
6.01
12.00
f1 (ppm)

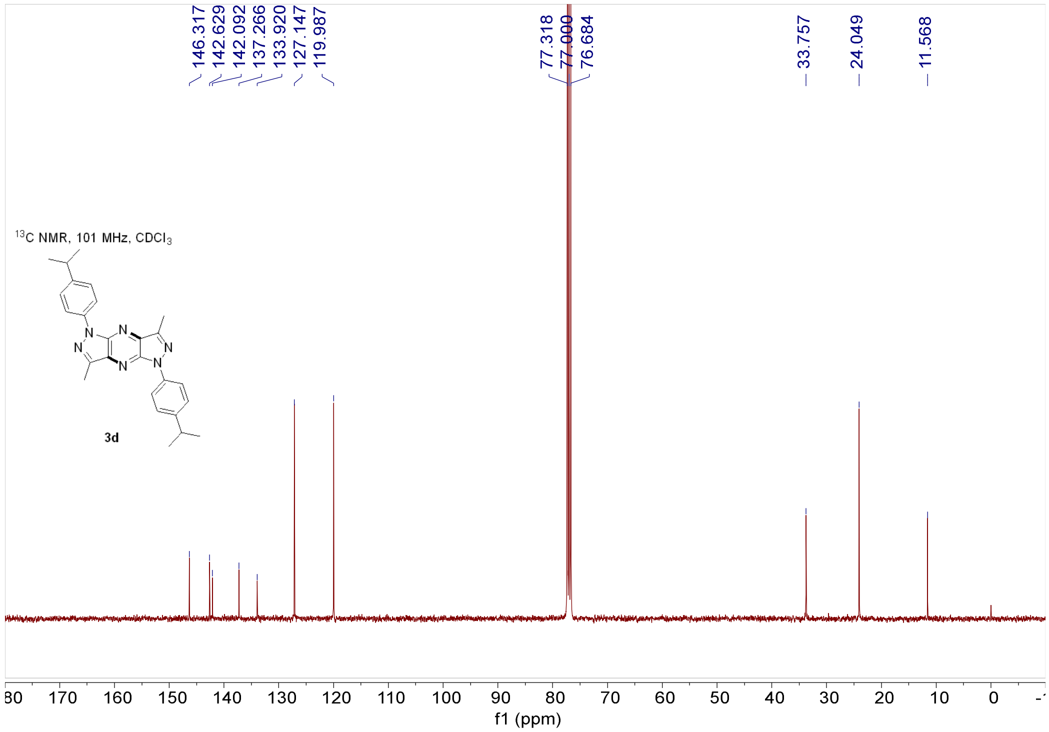
146.317
142.629
142.092
137.266
133.920
127.147
119.987
77.318
77.000
76.684
33.757
24.049
11.568
13C NMR, 101 MHz, CDCl3
3d
f1 (ppm)

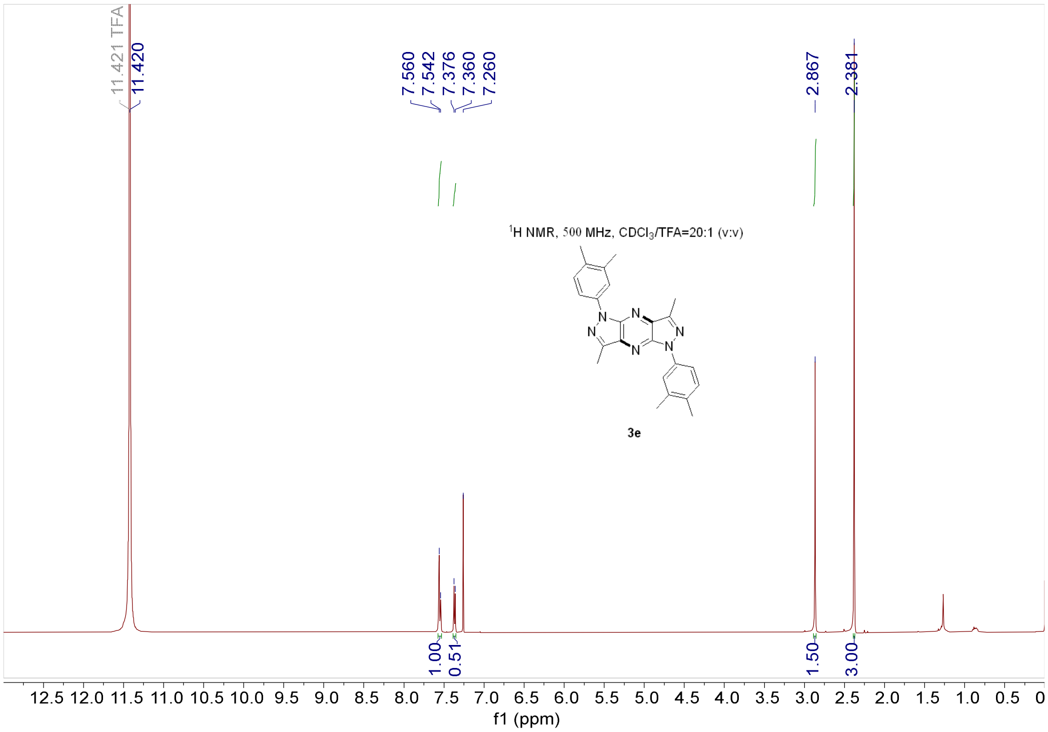
11.421 TFA
11.420
7.560
7.542
7.376
7.360
7.260
2.867
2.381
1H NMR, 500 MHz, CDCl3/TFA=20:1 (v:v)
3e
1.00
0.51
1.50
3.00
f1 (ppm)

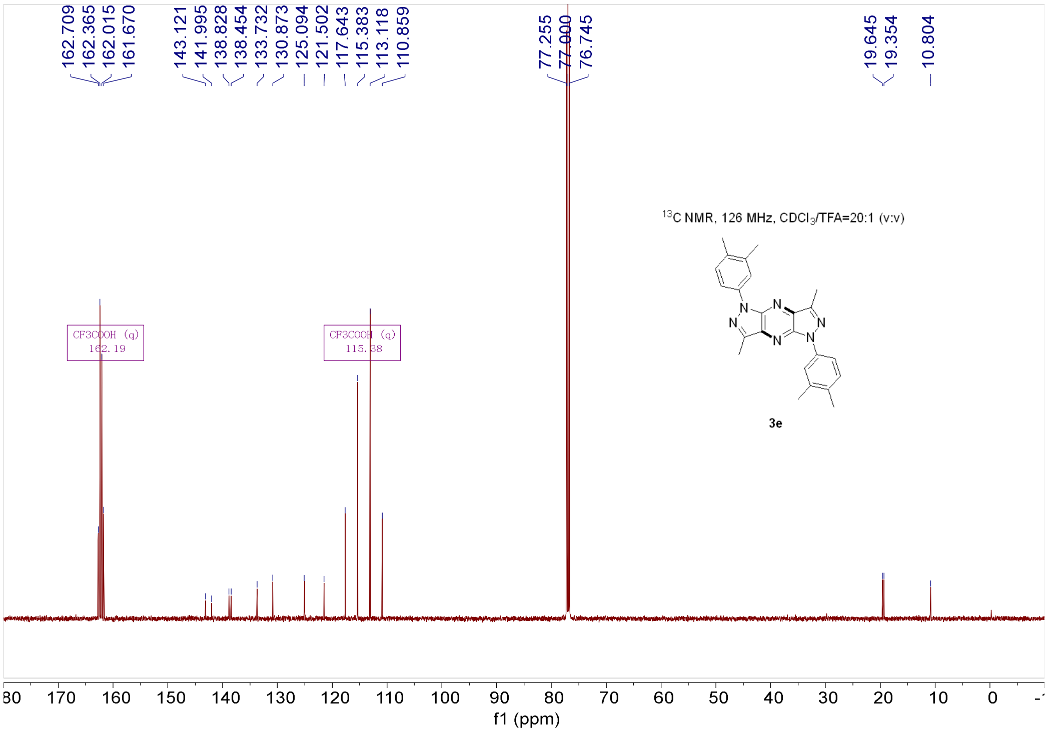
162.709
162.365
162.015
161.670
143.121
141.995
138.828
138.454
133.732
130.873
125.094
121.502
117.643
115.383
113.118
110.859
77.255
77.000
76.745
19.645
19.354
10.804
13C NMR, 126 MHz, CDCl3/TFA=20:1 (v:v)
CF3COOH (q)
162.19
CF3COOH (q)
115.38
3e
f1 (ppm)

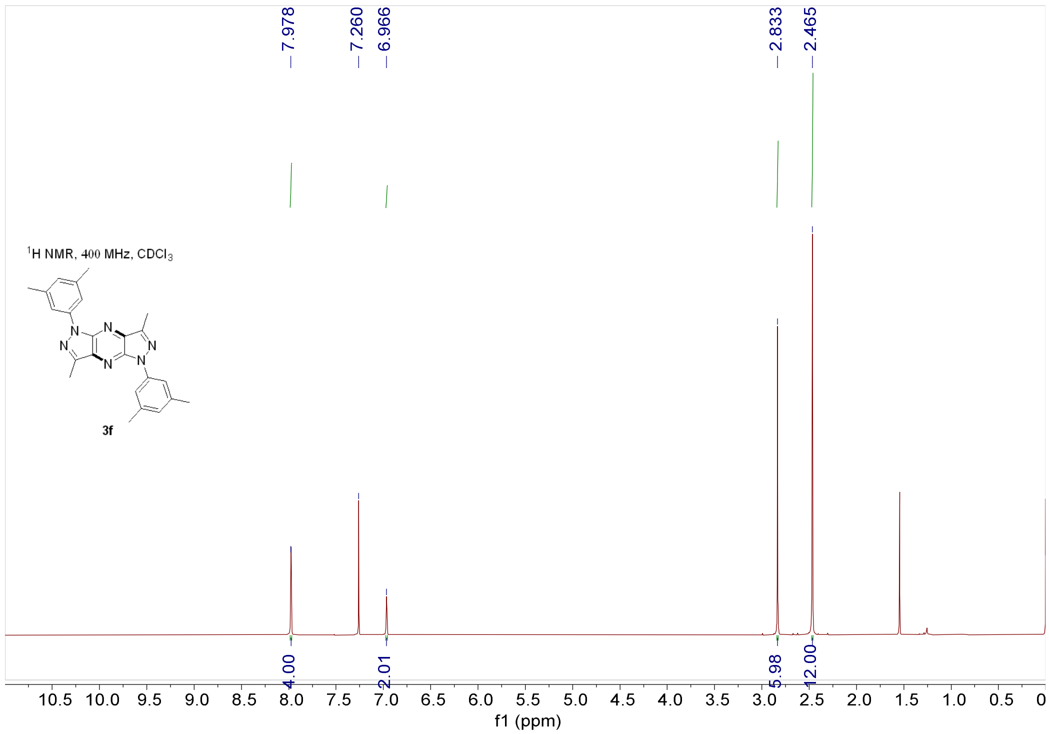

7.978
7.260
6.966
2.833
2.465
1H NMR, 400 MHz, CDCl3
3f
4.00
2.01
5.98
12.00
10.5 10.0 9.5 9.0 8.5 8.0 7.5 7.0 6.5 6.0 5.5 5.0 4.5 4.0 3.5 3.0 2.5 2.0 1.5 1.0 0.5 0
f1 (ppm)

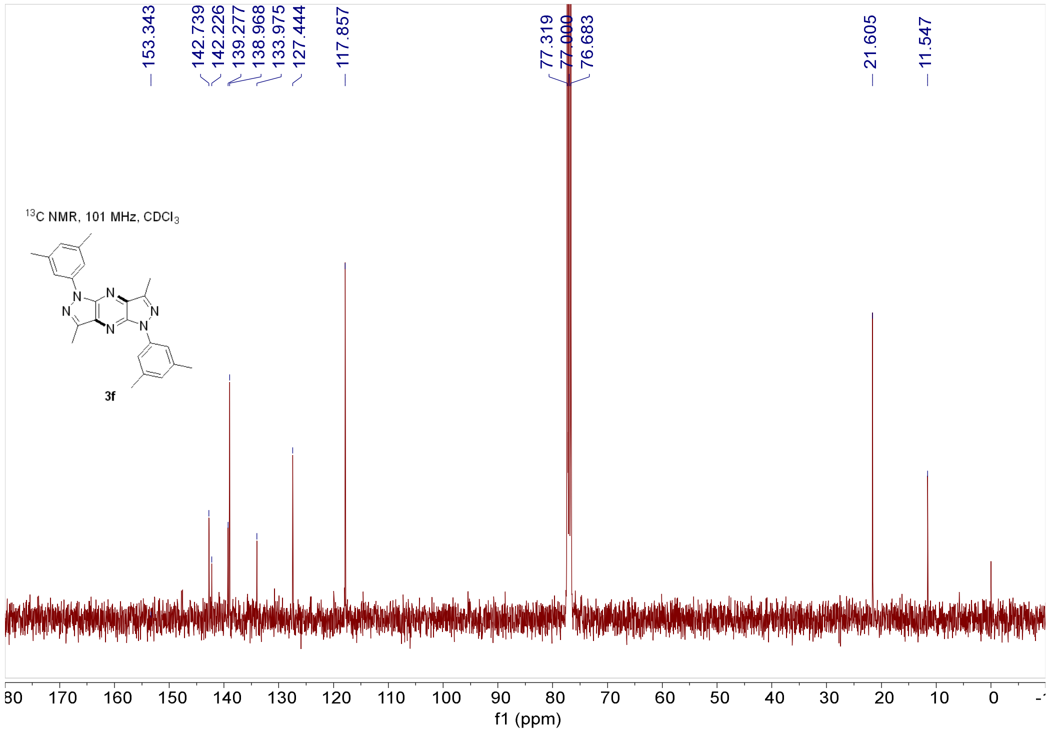

153.343
142.739
142.226
139.277
138.968
133.975
127.444
117.857
77.319
77.000
76.683
21.605
11.547
13C NMR, 101 MHz, CDCl3
3f
170 160 150 140 130 120 110 100 90 80 70 60 50 40 30 20 10 0
f1 (ppm)

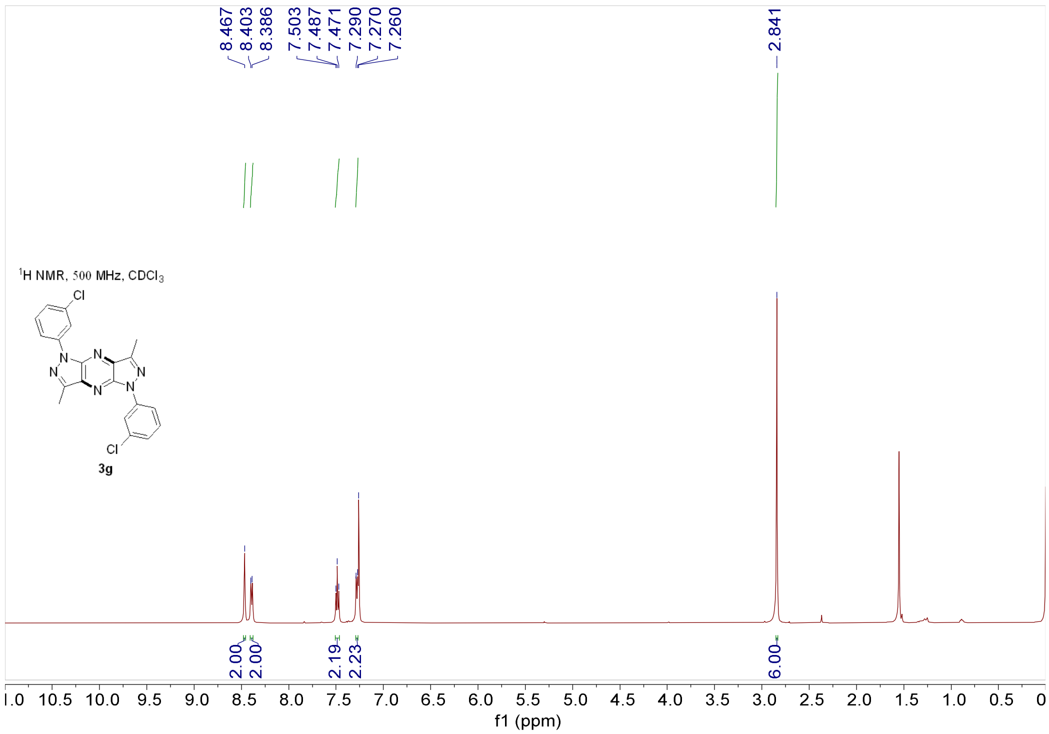
8.467
8.403
8.386
7.503
7.487
7.471
7.290
7.270
7.260
2.841
1H NMR, 500 MHz, CDCl3
Cl
N
N
N
N
N
N
Cl
3g
2.00
2.00
2.19
2.23
6.00
f1 (ppm)

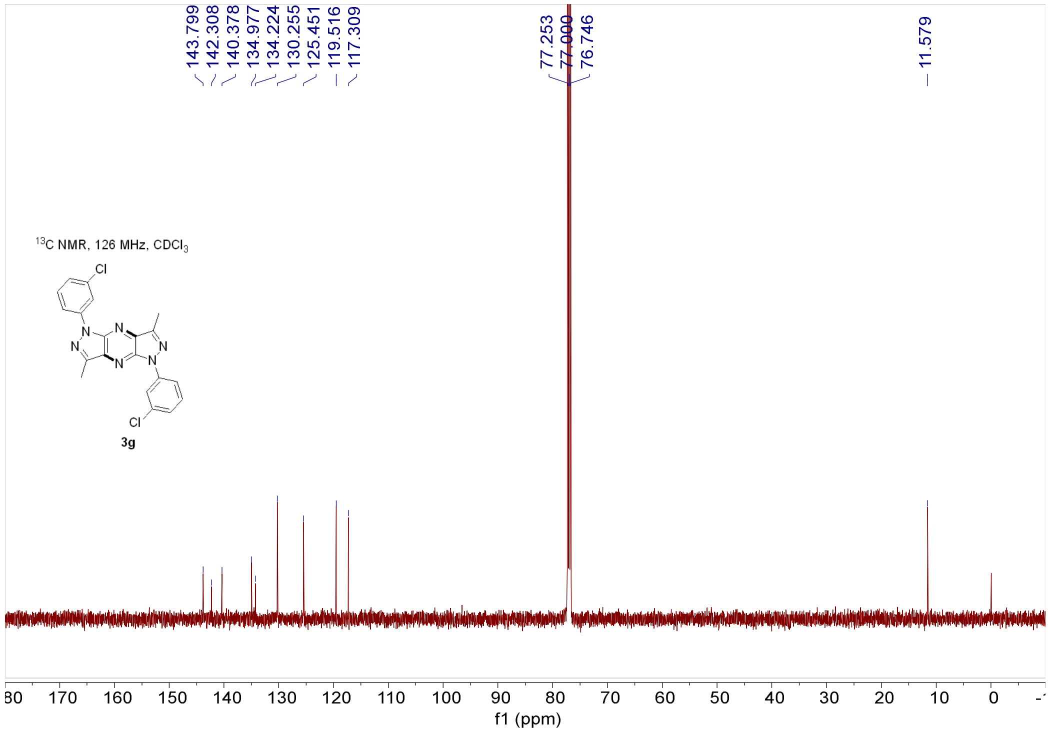
143.799
142.308
140.378
134.977
134.224
130.255
125.451
119.516
117.309
77.253
77.000
76.746
11.579
13C NMR, 126 MHz, CDCl3
Cl
N
N
N
N
N
N
Cl
3g
f1 (ppm)

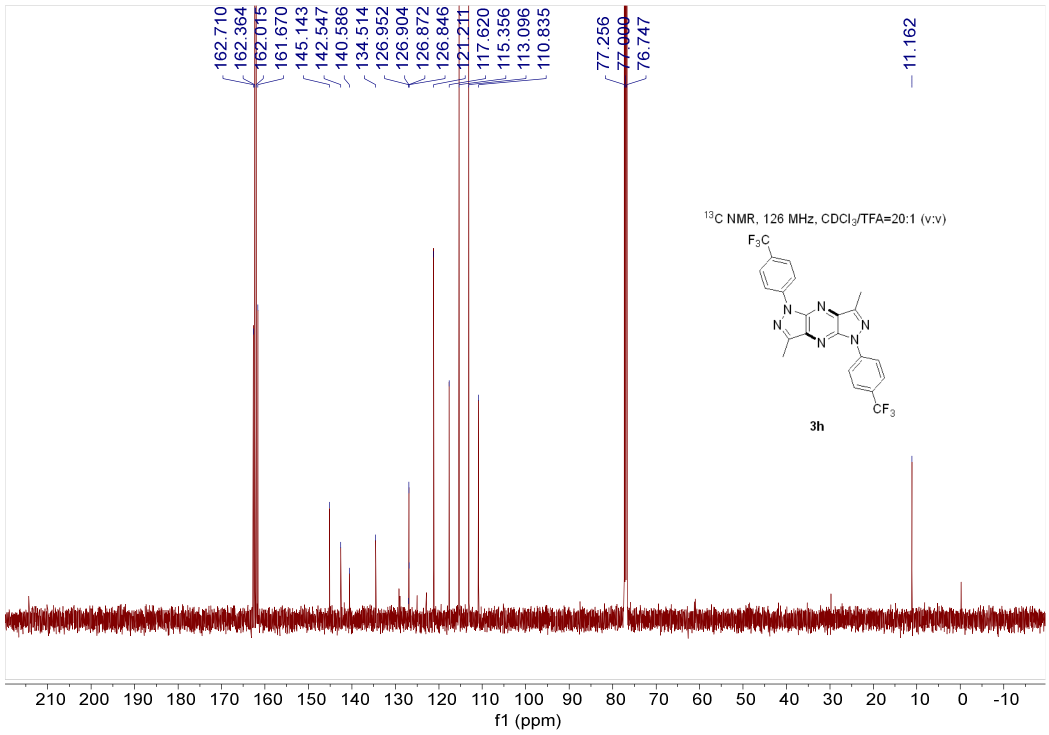
162.710
162.364
162.015
161.670
145.143
142.547
140.586
134.514
126.952
126.904
126.872
126.846
121.211
117.620
115.356
113.096
110.835
77.256
77.000
76.747
11.162
^{13}C NMR, 126 MHz, CDCl$_3$/TFA=20:1 (v:v)
F$_3$C
CF$_3$
3h
210 200 190 180 170 160 150 140 130 120 110 100 90 80 70 60 50 40 30 20 10 0 -10
f1 (ppm)

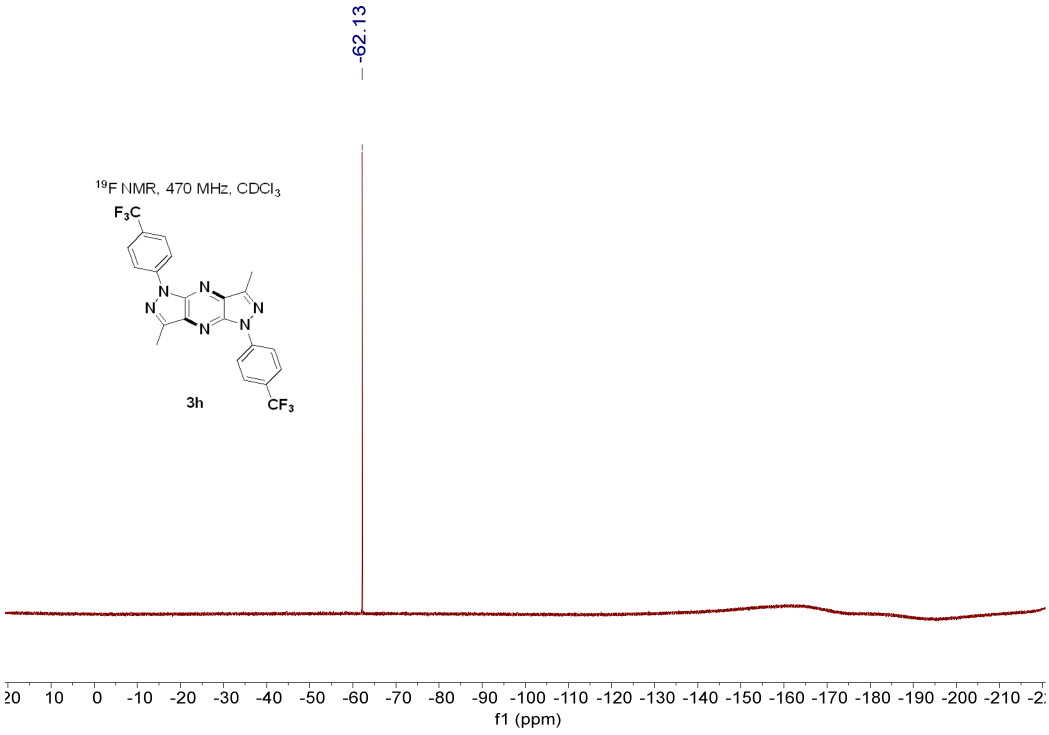
-62.13
^{19}F NMR, 470 MHz, CDCl$_3$
F$_3$C
CF$_3$
3h
20 10 0 -10 -20 -30 -40 -50 -60 -70 -80 -90 -100 -110 -120 -130 -140 -150 -160 -170 -180 -190 -200 -210
f1 (ppm)

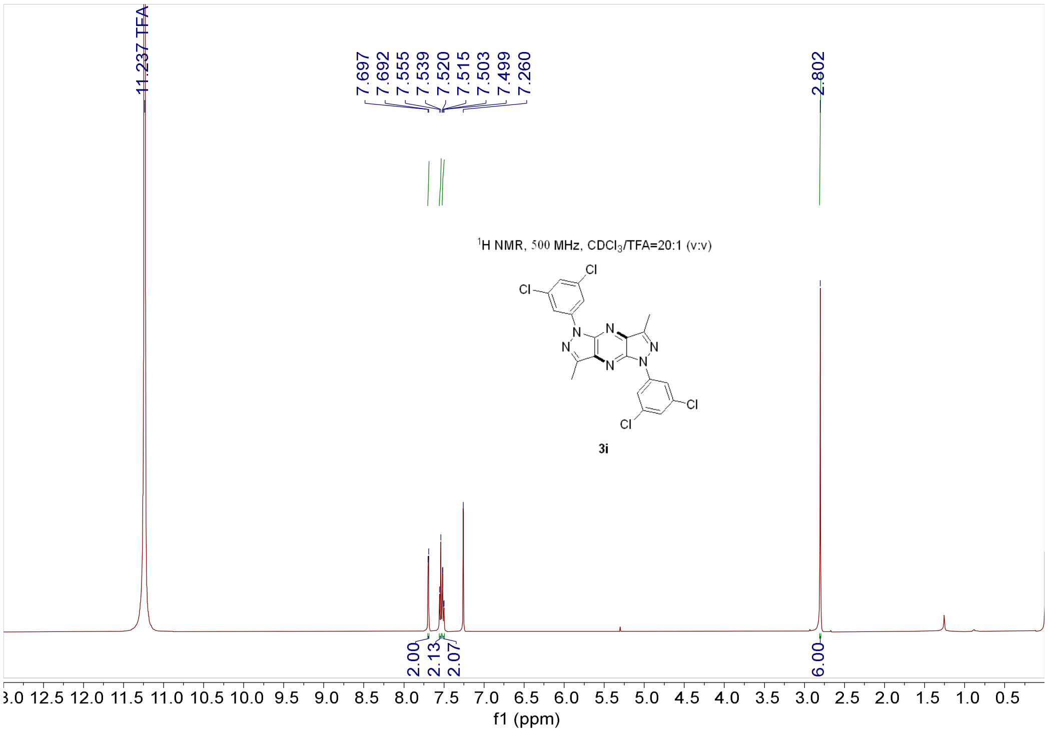

11.237 TFA
7.697
7.692
7.555
7.539
7.520
7.515
7.503
7.499
7.260
2.802
^{1}H NMR, 500 MHz, CDCl$_3$/TFA=20:1 (v:v)
Cl
Cl
N
N
N
N
N
N
N
N
Cl
Cl
3i
2.00
2.13
2.07
6.00
3.0 12.5 12.0 11.5 11.0 10.5 10.0 9.5 9.0 8.5 8.0 7.5 7.0 6.5 6.0 5.5 5.0 4.5 4.0 3.5 3.0 2.5 2.0 1.5 1.0 0.5
f1 (ppm)

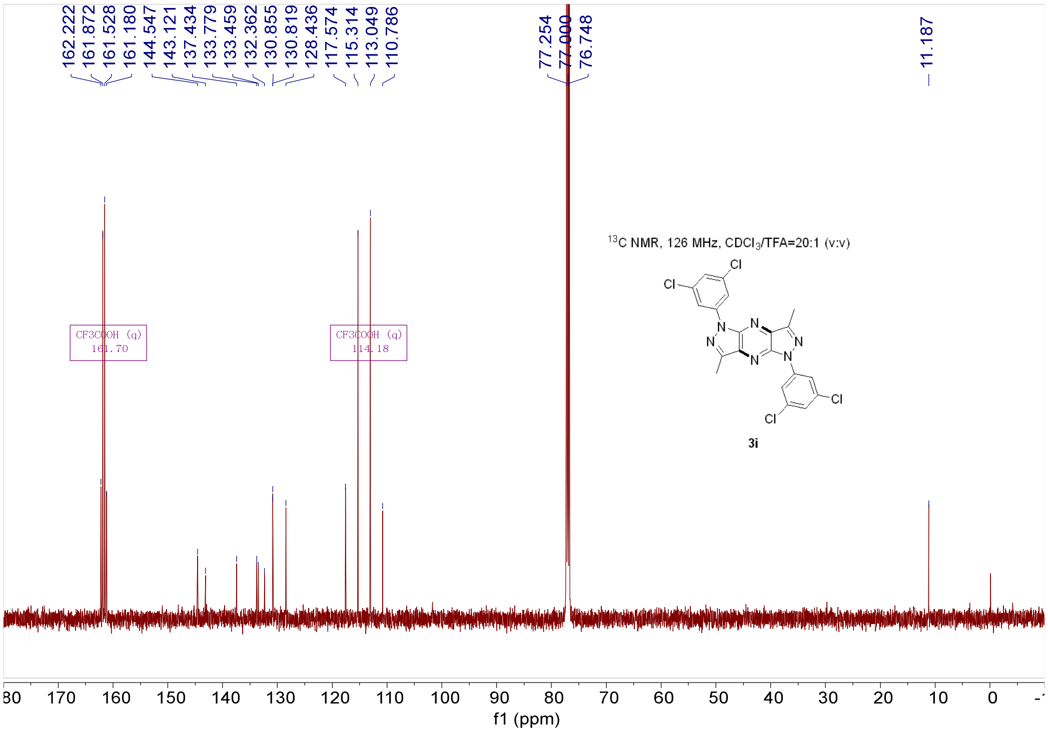

162.222
161.872
161.528
161.180
144.547
143.121
137.434
133.779
133.459
132.362
130.855
130.819
128.436
117.574
115.314
113.049
110.786
77.254
77.000
76.748
11.187
^{13}C NMR, 126 MHz, CDCl$_3$/TFA=20:1 (v:v)
CF3COOH (q)
161.70
CF3COOH (q)
114.18
Cl
Cl
N
N
N
N
N
N
N
N
Cl
Cl
3i
80 170 160 150 140 130 120 110 100 90 80 70 60 50 40 30 20 10 0
f1 (ppm)

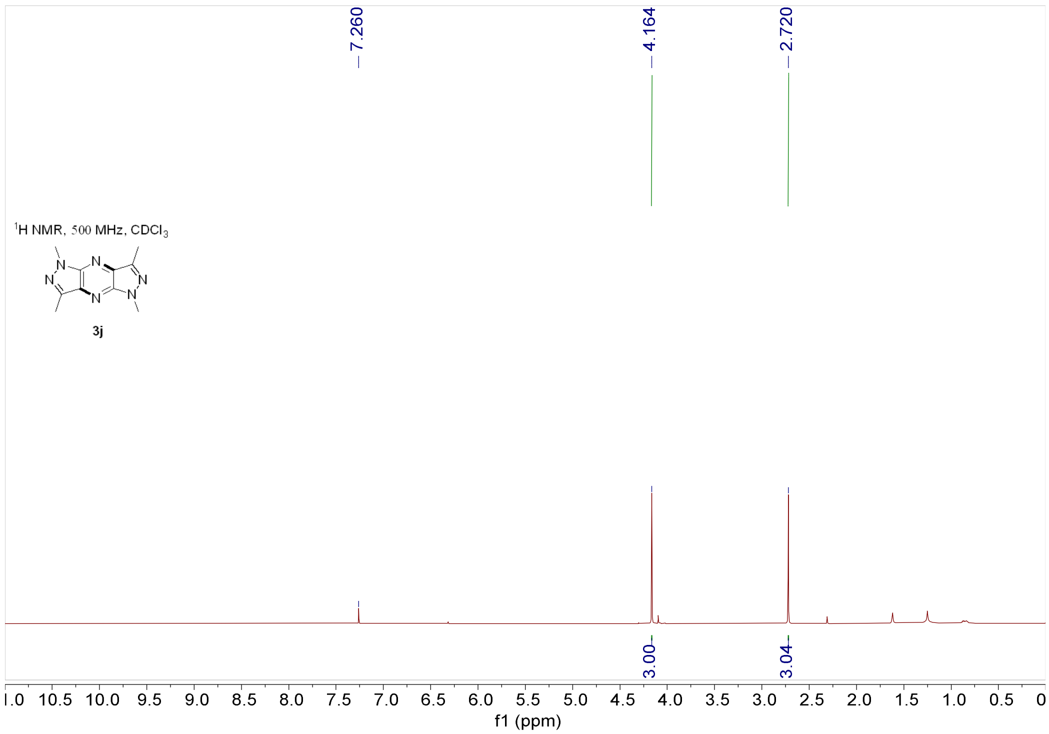
7.260
4.164
2.720
1H NMR, 500 MHz, CDCl3
3j
3.00
3.04
f1 (ppm)

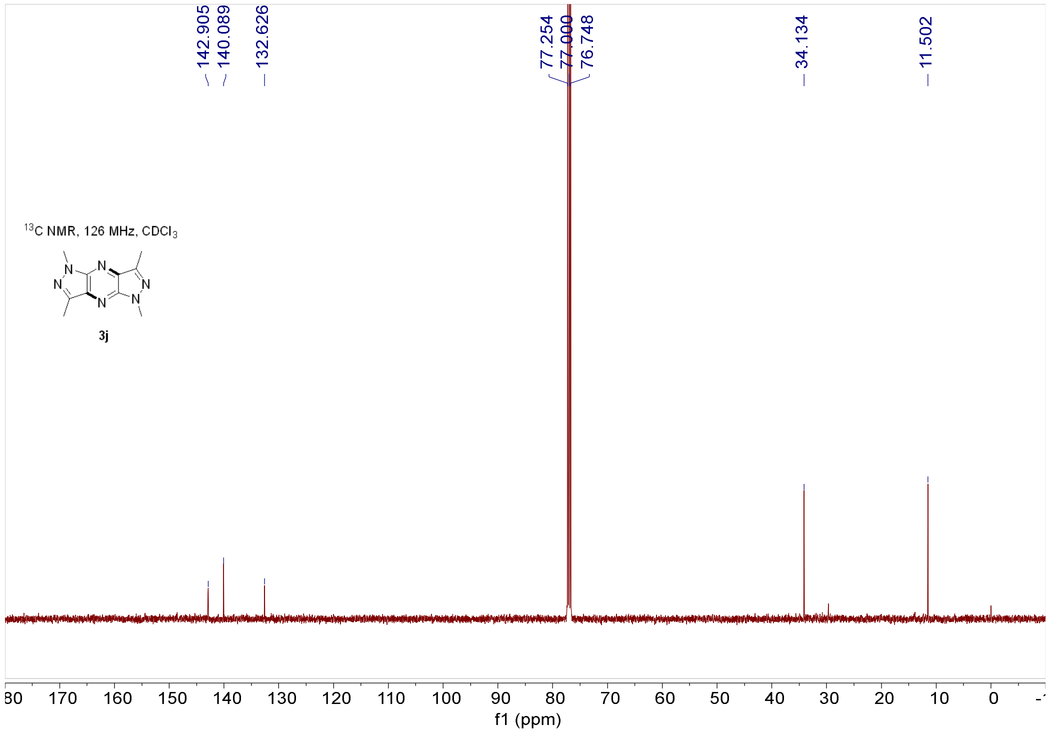
142.905
140.089
132.626
77.254
77.000
76.748
34.134
11.502
13C NMR, 126 MHz, CDCl3
3j
f1 (ppm)

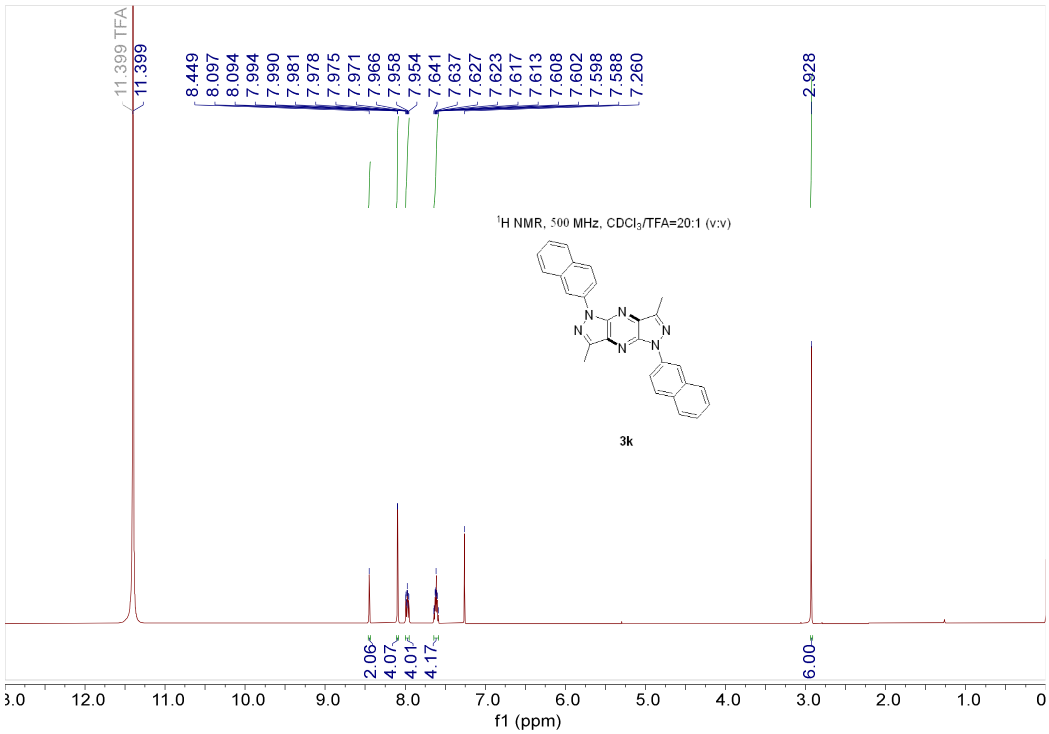

11.399 TFA
11.399
8.449 8.097 8.094 7.994 7.990 7.981 7.978 7.975 7.971 7.966 7.958 7.954 7.641 7.637 7.627 7.623 7.617 7.613 7.608 7.602 7.598 7.588 7.260
2.928
¹H NMR, 500 MHz, CDCl3/TFA=20:1 (v:v)
3k
2.06 4.07 4.01 4.17
6.00
f1 (ppm)

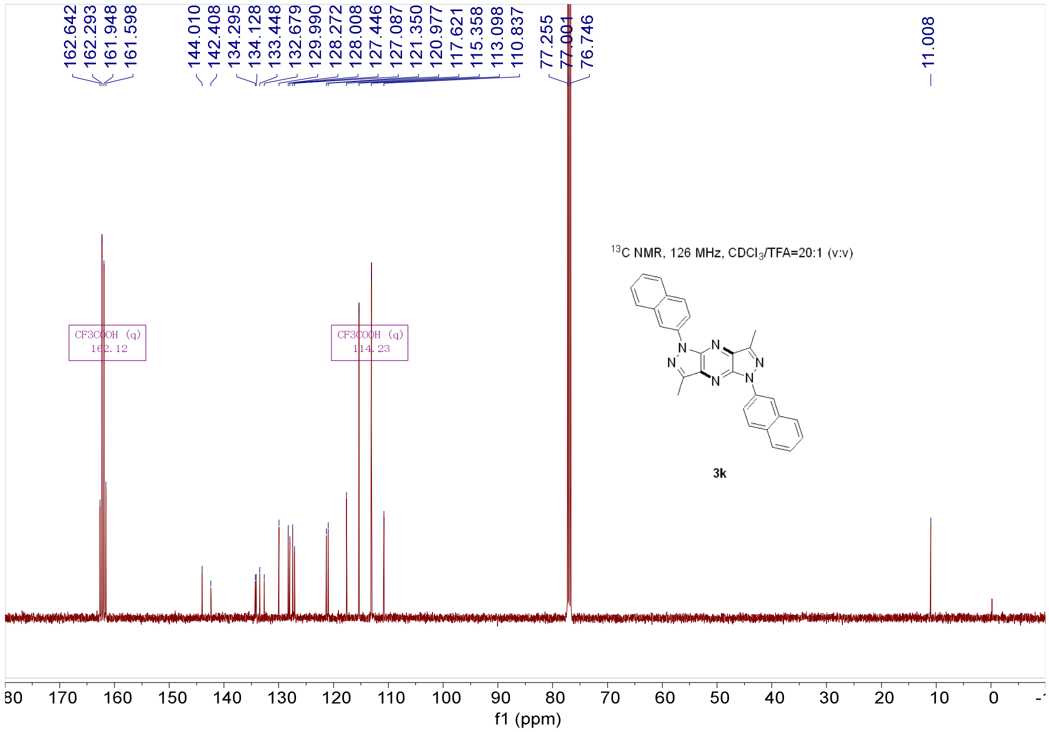

162.642 162.293 161.948 161.598
144.010 142.408 134.295 134.128 133.448 132.679 129.990 128.272 128.008 127.446 127.087 121.350 120.977 117.621 115.358 113.098 110.837
77.255 77.001 76.746
11.008
¹³C NMR, 126 MHz, CDCl3/TFA=20:1 (v:v)
3k
CF3COOH (q) 162.12
CF3COOH (q) 114.23
f1 (ppm)

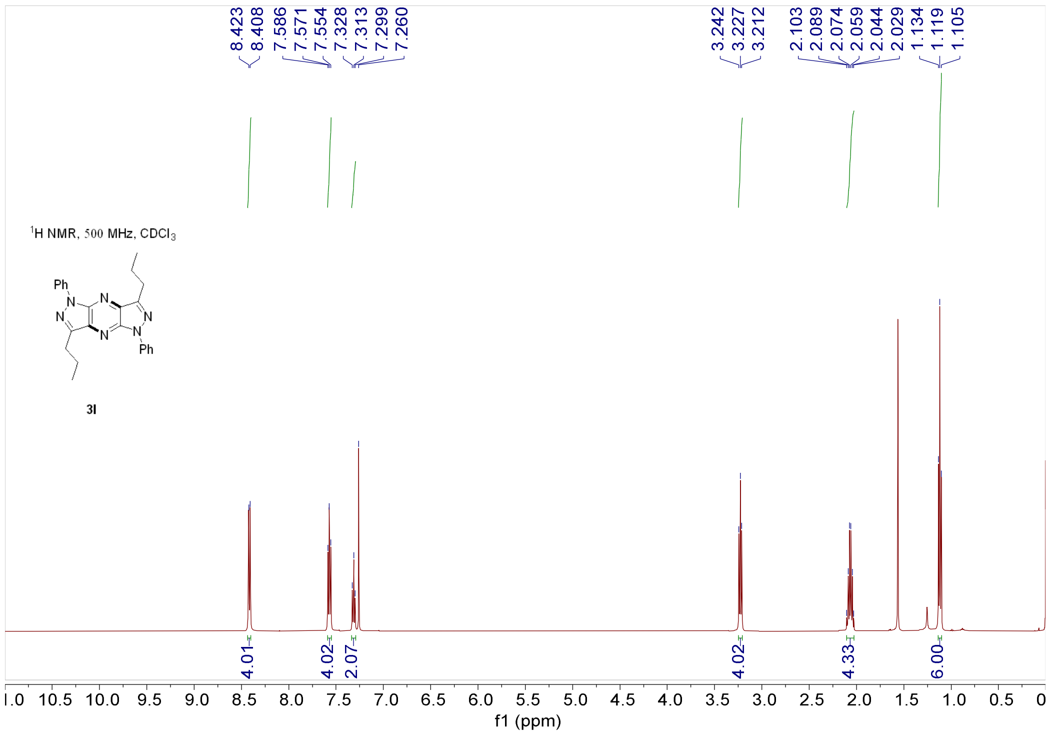
8.423
8.408
7.586
7.571
7.554
7.328
7.313
7.299
7.260
3.242
3.227
3.212
2.103
2.089
2.074
2.059
2.044
2.029
1.134
1.119
1.105
^{1}H NMR, 500 MHz, CDCl$_3$
Ph
N
N
N
N
N
N
Ph
3l
4.01
4.02
2.07
4.02
4.33
6.00
f1 (ppm)

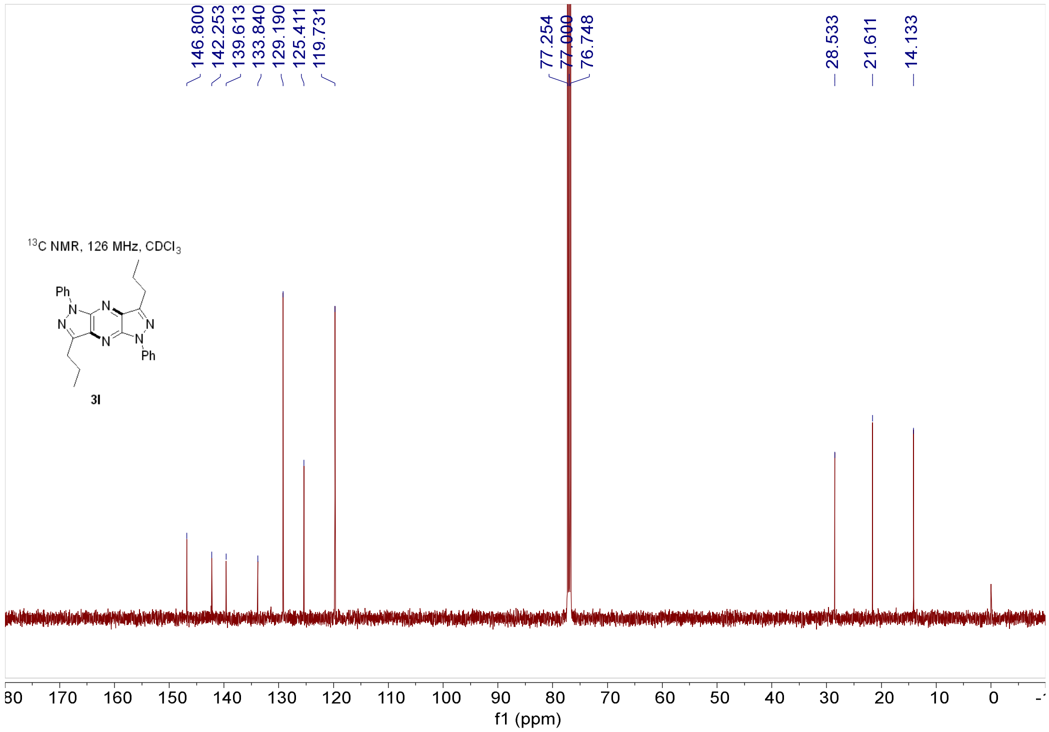
146.800
142.253
139.613
133.840
129.190
125.411
119.731
77.254
77.000
76.748
28.533
21.611
14.133
^{13}C NMR, 126 MHz, CDCl$_3$
Ph
N
N
N
N
N
N
Ph
3l
f1 (ppm)

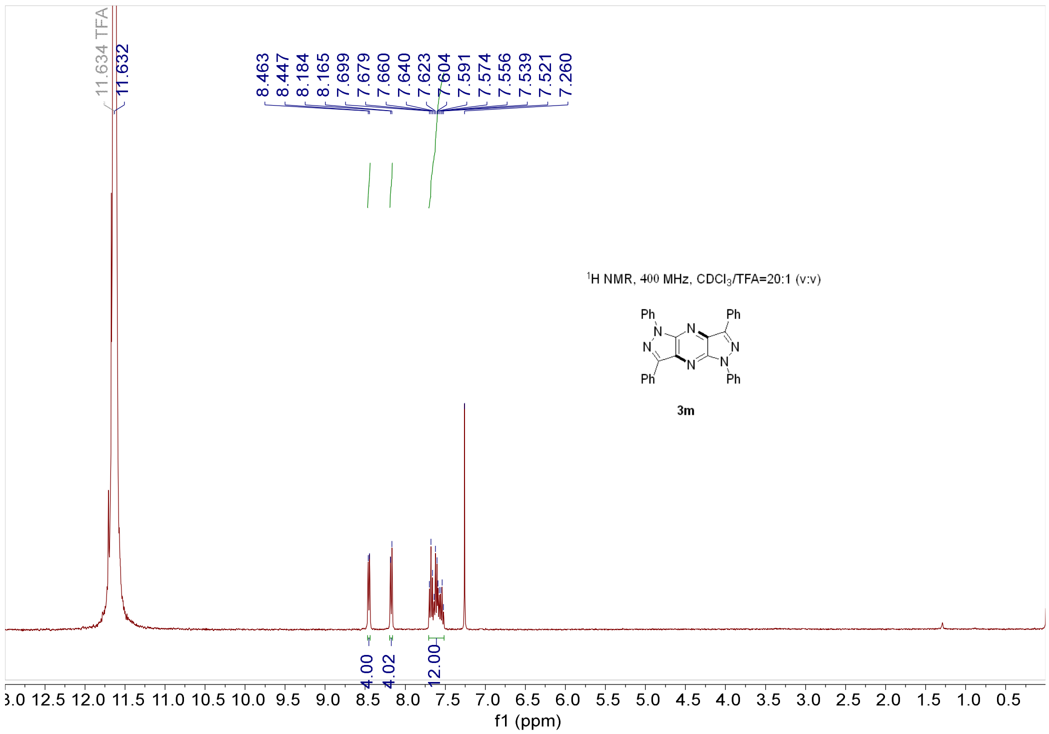
11.634 TFA
11.632
8.463
8.447
8.184
8.165
7.699
7.679
7.660
7.640
7.623
7.604
7.591
7.574
7.556
7.539
7.521
7.260
1H NMR, 400 MHz, CDCl3/TFA=20:1 (v:v)
3m
4.00
4.02
12.00
f1 (ppm)

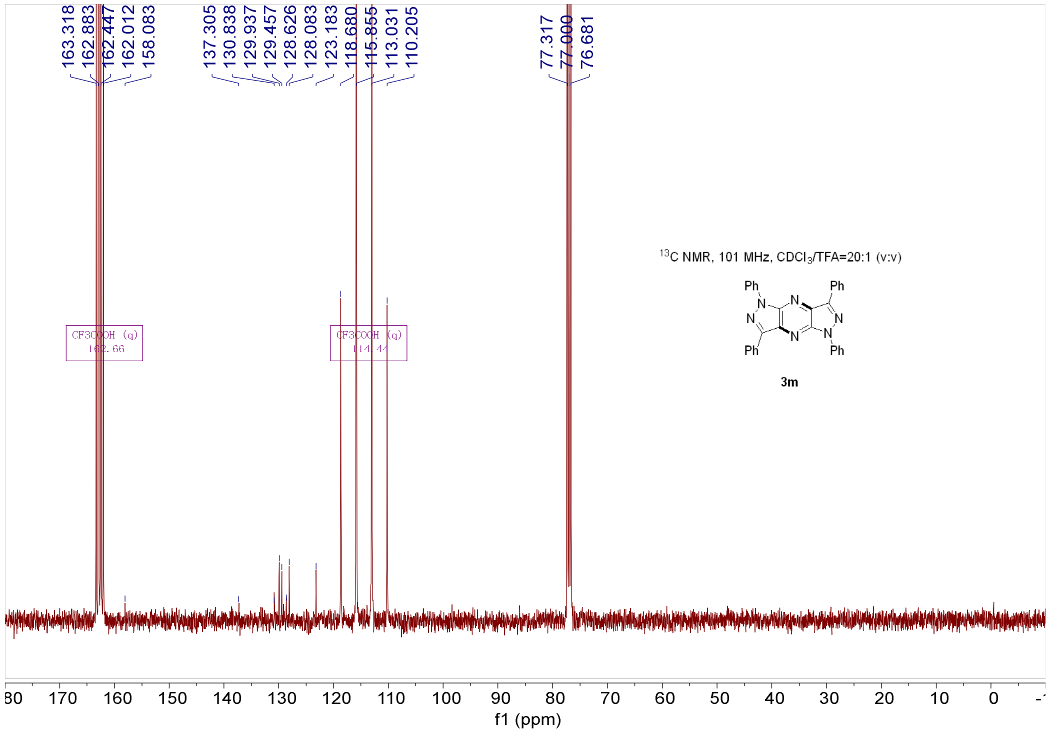
163.318
162.883
162.447
162.012
158.083
137.305
130.838
129.937
129.457
128.626
128.083
123.183
118.680
115.855
113.031
110.205
77.317
77.000
76.681
13C NMR, 101 MHz, CDCl3/TFA=20:1 (v:v)
CF3COOH (q)
162.66
CF3COOH (q)
114.44
3m
f1 (ppm)

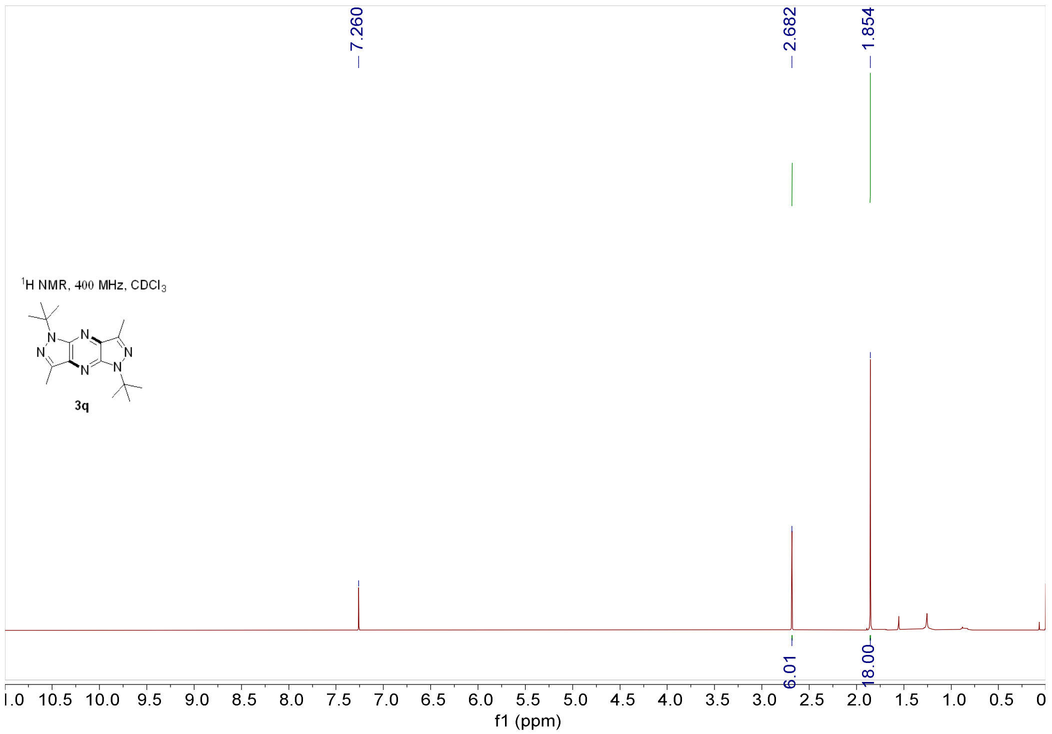
7.260
2.682
1.854
^{1}H NMR, 400 MHz, CDCl3
3q
6.01
18.00
f1 (ppm)

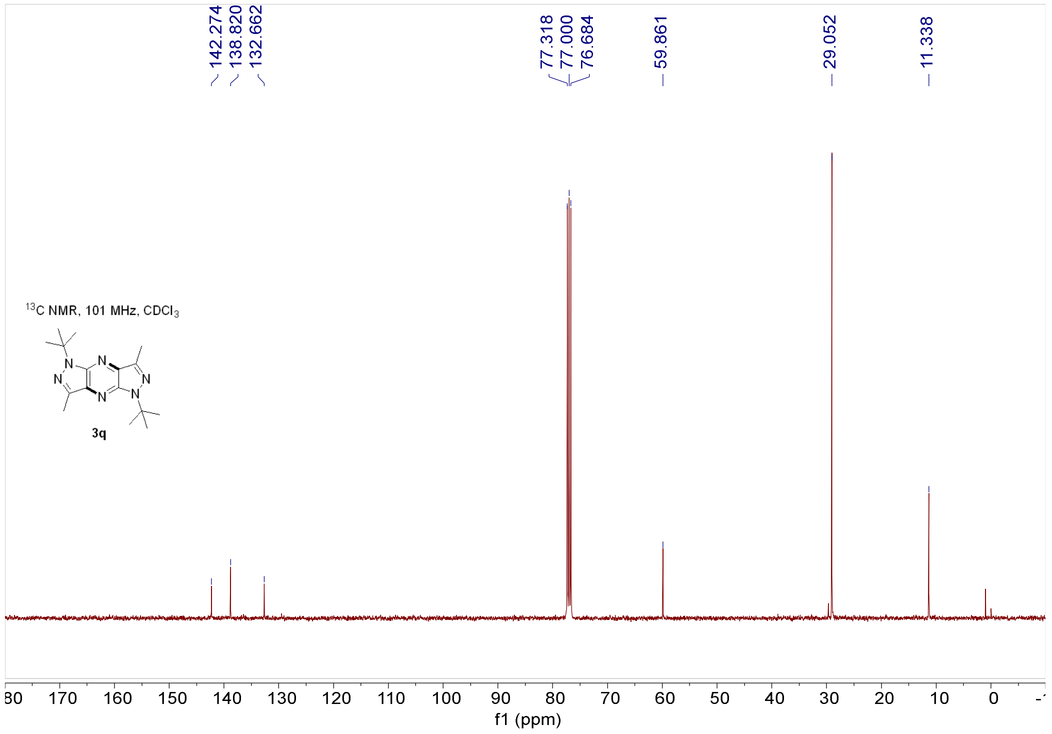
142.274
138.820
132.662
77.318
77.000
76.684
59.861
29.052
11.338
^{13}C NMR, 101 MHz, CDCl3
3q
f1 (ppm)

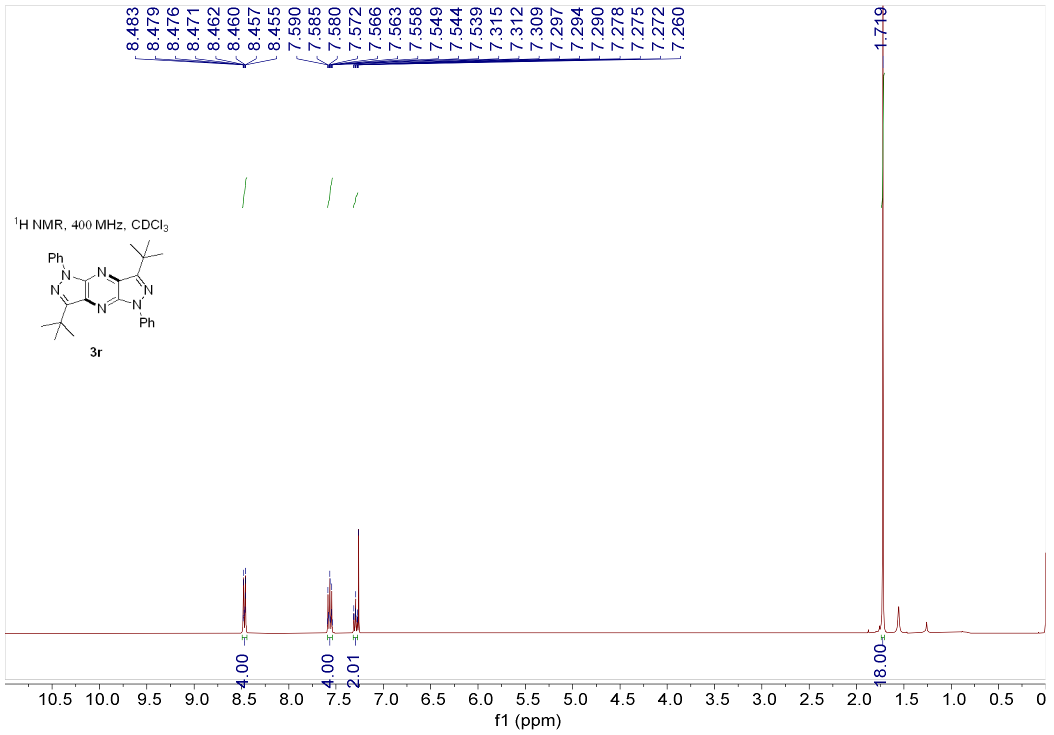
8.483
8.479
8.476
8.471
8.462
8.460
8.457
8.455
7.590
7.585
7.580
7.572
7.566
7.563
7.558
7.549
7.544
7.539
7.315
7.312
7.309
7.297
7.294
7.290
7.278
7.275
7.272
7.260
1.719
^{1}H NMR, 400 MHz, CDCl$_3$
Ph
N
N
N
N
N
N
N
Ph
3r
4.00
4.00
2.01
18.00
10.5 10.0 9.5 9.0 8.5 8.0 7.5 7.0 6.5 6.0 5.5 5.0 4.5 4.0 3.5 3.0 2.5 2.0 1.5 1.0 0.5 0
f1 (ppm)

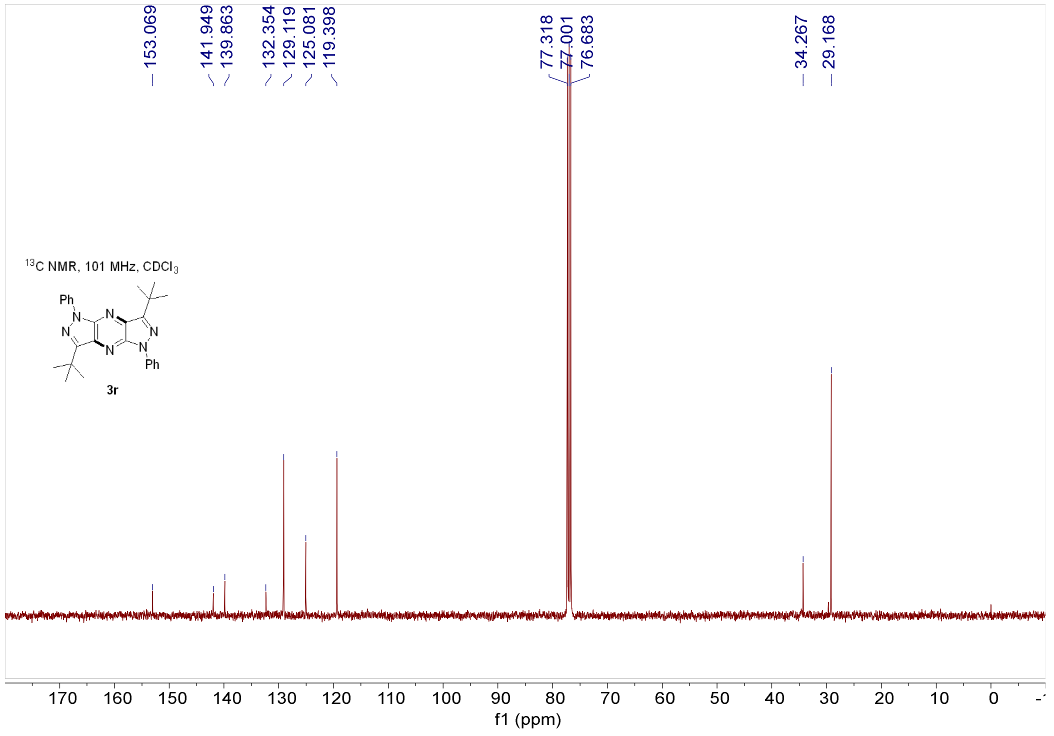
153.069
141.949
139.863
132.354
129.119
125.081
119.398
77.318
77.001
76.683
34.267
29.168
^{13}C NMR, 101 MHz, CDCl$_3$
Ph
N
N
N
N
N
N
N
Ph
3r
170 160 150 140 130 120 110 100 90 80 70 60 50 40 30 20 10 0
f1 (ppm)

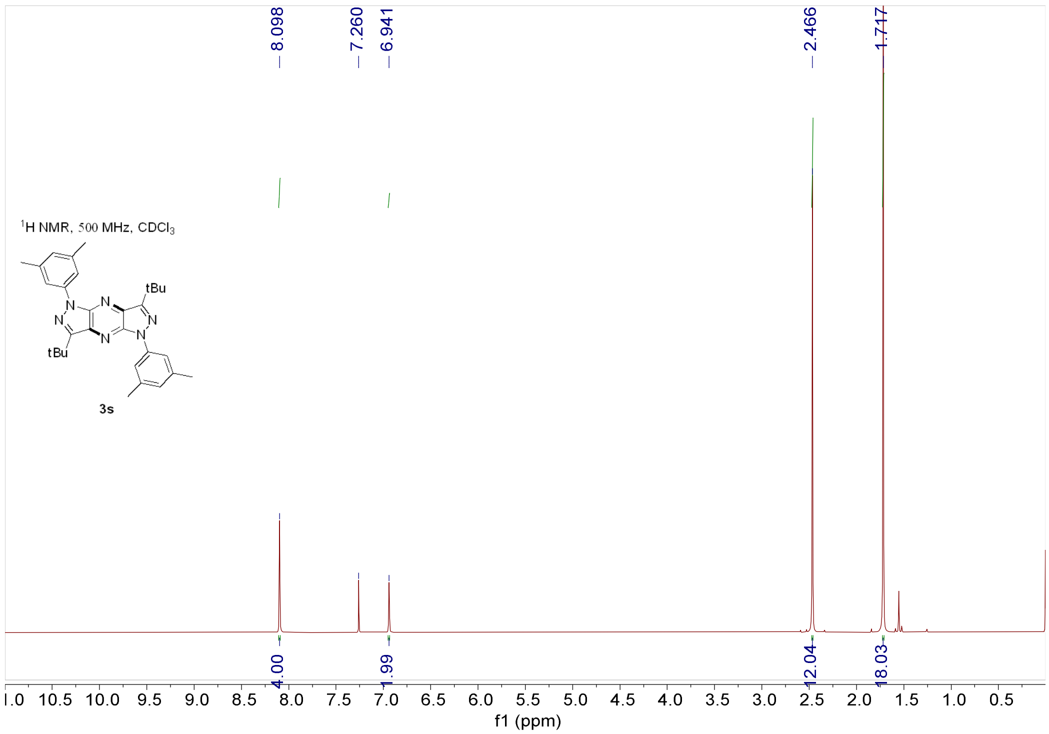
8.098
7.260
6.941
2.466
1.717
1H NMR, 500 MHz, CDCl3
tBu
tBu
N
3s
4.00
1.99
12.04
18.03
f1 (ppm)

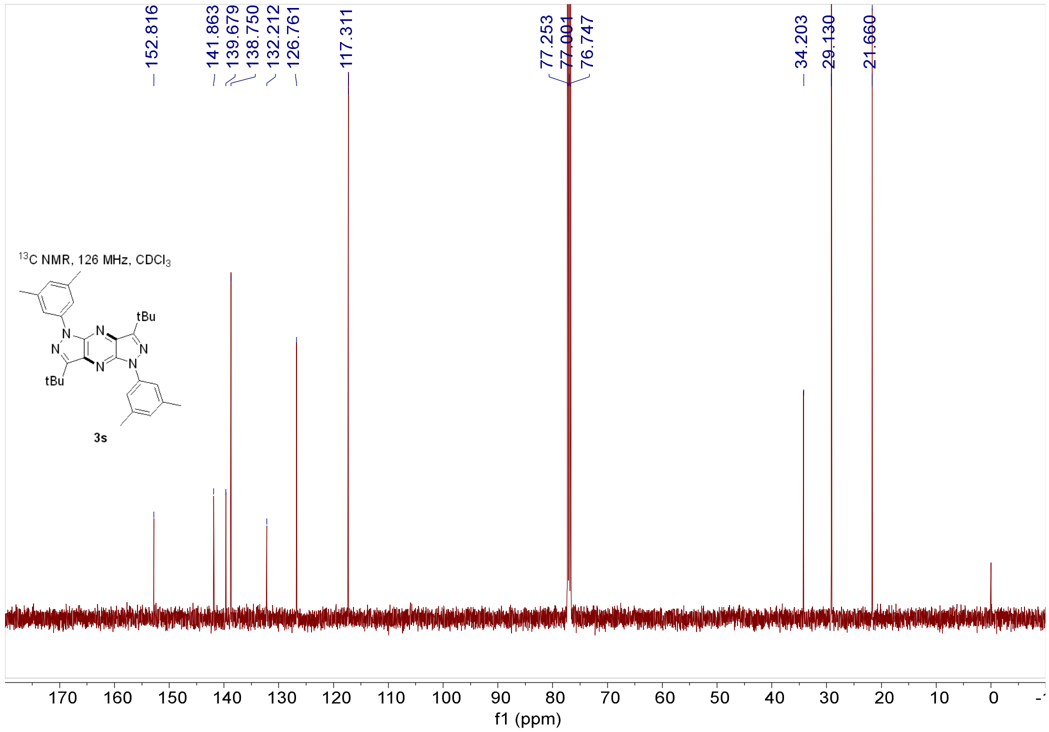
152.816
141.863
139.679
138.750
132.212
126.761
117.311
77.253
77.001
76.747
34.203
29.130
21.660
13C NMR, 126 MHz, CDCl3
tBu
tBu
N
3s
f1 (ppm)

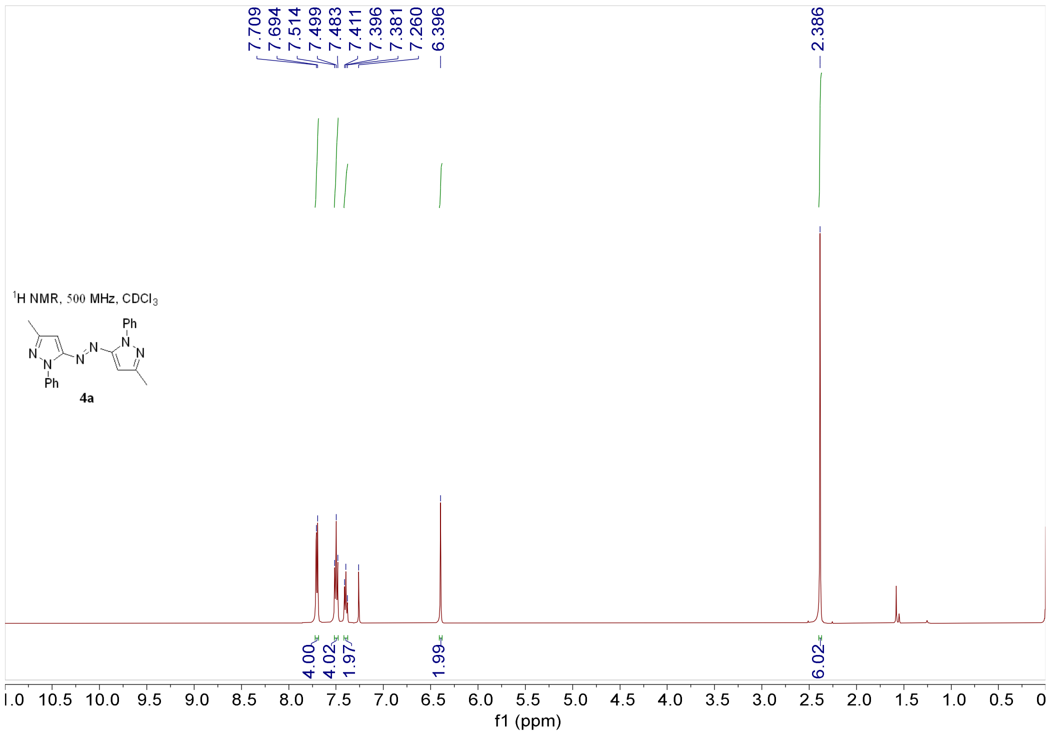
7.709
7.694
7.514
7.499
7.483
7.411
7.396
7.381
7.260
6.396
2.386
1H NMR, 500 MHz, CDCl3
Ph
N
N
N
N
N
N
Ph
4a
4.00
4.02
1.97
1.99
6.02
f1 (ppm)

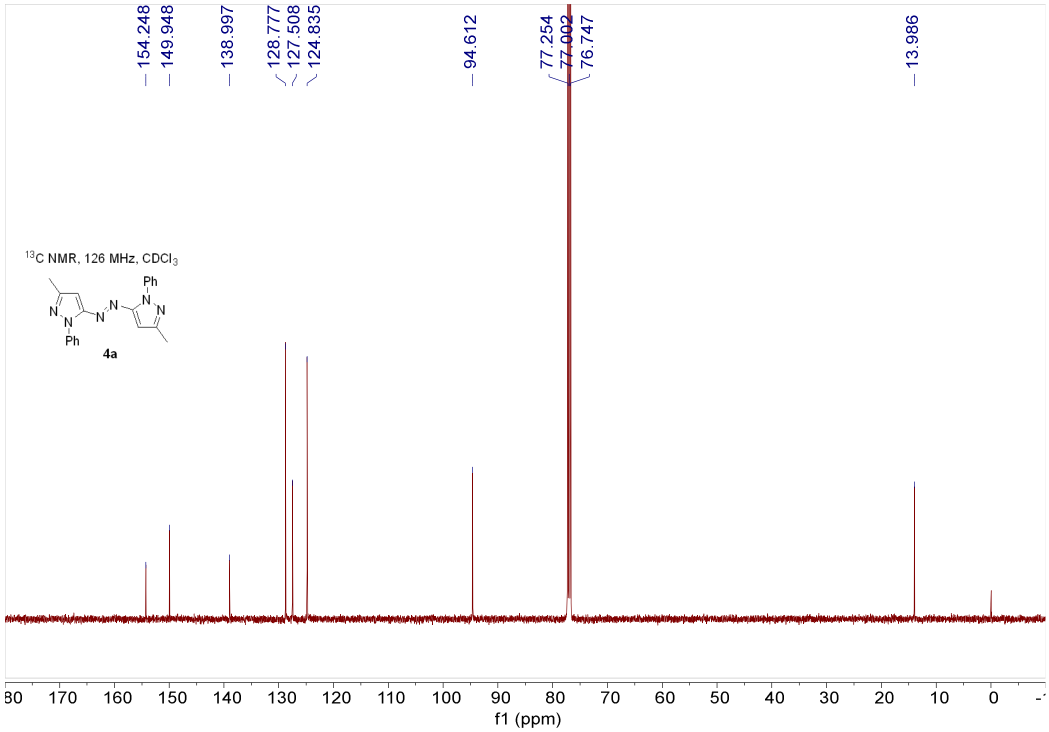
154.248
149.948
138.997
128.777
127.508
124.835
94.612
77.254
77.002
76.747
13.986
13C NMR, 126 MHz, CDCl3
Ph
N
N
N
N
N
N
Ph
4a
f1 (ppm)

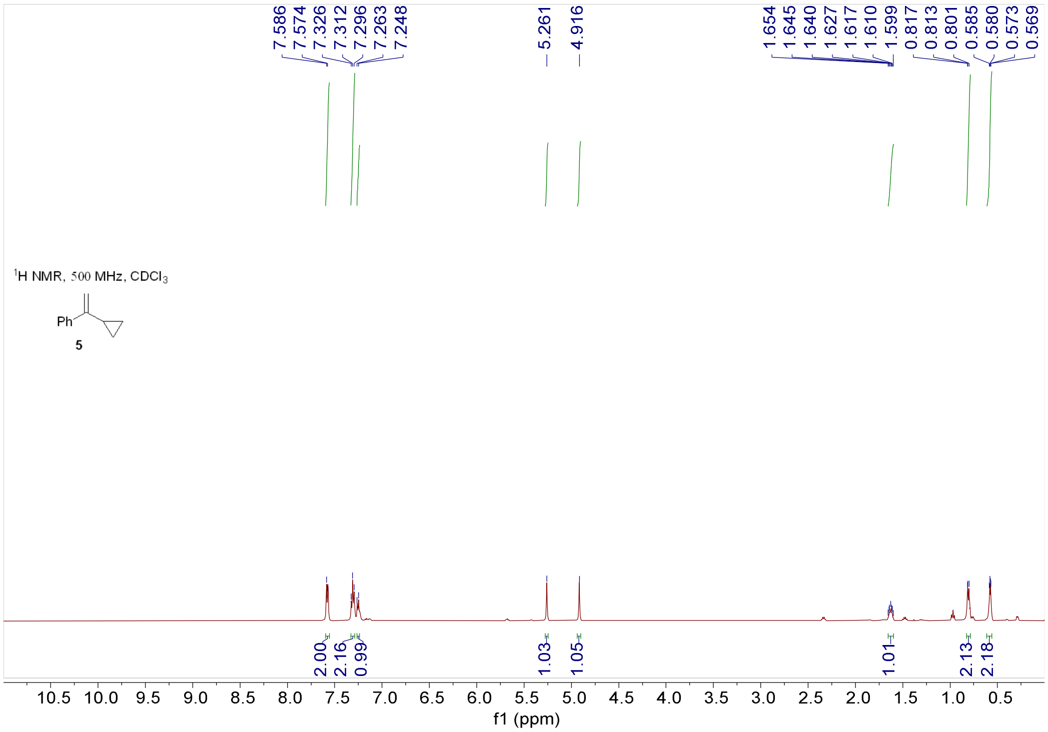
7.586
7.574
7.326
7.312
7.296
7.263
7.248
5.261
4.916
1.654
1.645
1.640
1.627
1.617
1.610
1.599
0.817
0.813
0.801
0.585
0.580
0.573
0.569
1H NMR, 500 MHz, CDCl3
Ph
5
2.00
2.16
0.99
1.03
1.05
1.01
2.13
2.18
f1 (ppm)

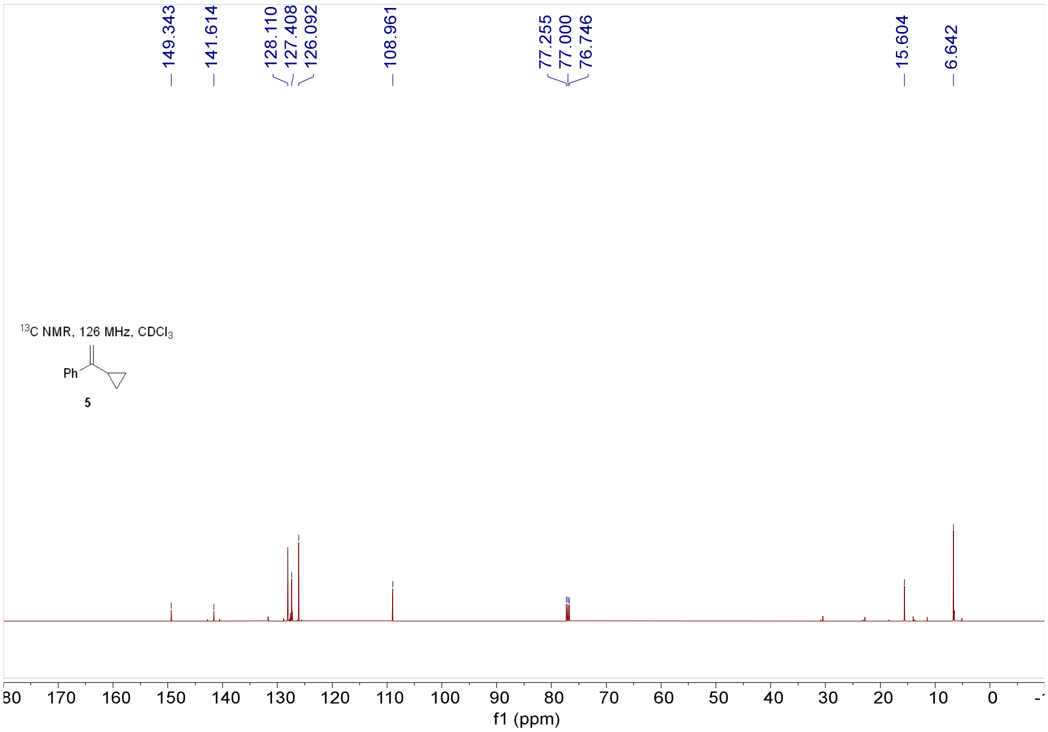
149.343
141.614
128.110
127.408
126.092
108.961
77.255
77.000
76.746
15.604
6.642
13C NMR, 126 MHz, CDCl3
Ph
5
f1 (ppm)
